# Zinc regulates ERp44-dependent protein quality control in the early secretory pathway

**DOI:** 10.1038/s41467-019-08429-1

**Published:** 2019-02-05

**Authors:** Satoshi Watanabe, Yuta Amagai, Sara Sannino, Tiziana Tempio, Tiziana Anelli, Manami Harayama, Shoji Masui, Ilaria Sorrentino, Momo Yamada, Roberto Sitia, Kenji Inaba

**Affiliations:** 1 Institute of Multidisciplinary Research for Advanced Materials, Tohoku University, Sendai, 980-8577 Japan; 2 Division of Genetics and Cell Biology, Vita-Salute University, IRCCS Ospedale San Raffaele, 20132 Milan, Italy; 3 Core Research for Evolutional Science and Technology (CREST), Japan Science and Technology Agency (JST), Kawaguchi, 332-0012 Japan; 4 Present Address: Department of Biological Sciences, University of Pittsburgh, A320 Langley Hall, 4249 Fifth Ave, Pittsburgh, PA 15260 USA

## Abstract

Zinc ions (Zn^2+^) are imported into the early secretory pathway by Golgi-resident transporters, but their handling and functions are not fully understood. Here, we show that Zn^2+^ binds with high affinity to the pH-sensitive chaperone ERp44, modulating its localization and ability to retrieve clients like Ero1α and ERAP1 to the endoplasmic reticulum (ER). Silencing the Zn^2+^ transporters that uptake Zn^2+^ into the Golgi led to ERp44 dysfunction and increased secretion of Ero1α and ERAP1. High-resolution crystal structures of Zn^2+^-bound ERp44 reveal that Zn^2+^ binds to a conserved histidine-cluster. The consequent large displacements of the regulatory C-terminal tail expose the substrate-binding surface and RDEL motif, ensuring client capture and retrieval. ERp44 also forms Zn^2+^-bridged homodimers, which dissociate upon client binding. Histidine mutations in the Zn^2+^-binding sites compromise ERp44 activity and localization. Our findings reveal a role of Zn^2+^ as a key regulator of protein quality control at the ER-Golgi interface.

## Abstract (web summary)

Zinc ions (Zn^2+^) are imported by Golgi-resident transporters but the function of zinc in the early secretory pathway has remained unknown. Here the authors find that Zn^2+^ regulates protein quality control in the early secretory pathway by demonstrating that the pH-sensitive chaperone ERp44 binds Zn^2+^ and solving the Zn^2+^-bound ERp44 structure.

## Introduction

Zinc ions (Zn^2+^) are essential cofactors for a variety of proteins^1,2^. The metal ions serve as enzyme catalysts or as cofactors stabilizing the three-dimensional structures of proteins^3–5^. Moreover, free Zn^2+^ can also act as a second messenger in signal transduction^6–8^. Two families of transporters, ZnT (zinc transporter, SLC30) and ZIP (Zrt/Irt-like protein, SLC39), mediate Zn^2+^ homeostasis in cells^9–12^. The human genome contains 9 ZnT and 14 ZIP proteins with different tissue and subcellular distribution^12^. ZIP members mediate Zn^2+^ import into the cytosol, whereas members of the ZnT family conduct its efflux from the cytosol into intracellular compartments or to the outside of the cell. In particular, ZnT5, 6, 7, and 10 are known to import Zn^2+^ into the Golgi^11^, where the metal can be incorporated into secretory metalloenzymes^13–19^. The abundance and localization of ZnTs and ZIPs in the early secretory pathway (ESP) are consistent with the fundamental role of Zn^2+^ in regulating the structure and function of many secretory proteins. However, how the metal is handled in ESP remains to be understood.

ERp44, a chaperone of the protein disulfide isomerase (PDI) family, cycles between the ER and *cis*-Golgi compartments to patrol proteins that pass through the BiP and Calnexin/Calreticulin checkpoints^20^. This chaperone controls the traffic and oligomeric assembly of various secretory proteins, including IgM and adiponectin^20,21^. ERp44’s clients also include several ER-resident enzymes that lack an ER-retention motif, such as Ero1α, Prx4, FGE/SUMF1, and ERAP1^22–26^. ERp44 traps its clients in the Golgi, retrieving them to the ER via KDEL receptors (KDELR)^27–31^. ERp44 is regulated by the ER–Golgi pH-gradient, which enables the protein to capture clients in the weakly acidic post-ER compartments and release them in the pH-neutral ER^32^.

The overall structure of ERp44 consists of three thioredoxin (Trx)-like domains (**a**, **b**, and **b**′) and a flexible C-terminal tail (C-tail) that terminates with a RDEL motif^33^. The high-resolution crystal structures of ERp44 at pH 6.5 and 7.2^34^ revealed that protonation of key histidine and cysteine residues induces pH-dependent conformational changes in ERp44, promoting the exposure of the positively charged client-binding site and allowing the essential cysteine (Cys29) to form intermolecular disulfide bonds with clients of thiol-mediated quality control^35,36^. Five conserved histidines located at the border between the **b**′ domain and the C-tail also have key roles in regulating the subcellular localization and client retention ability of ERp44^37^. Of note, the high-resolution structure of ERp44 revealed that the three most highly conserved histidines (His299, His328, His332) form a histidine-clustered site (His-cluster) seemingly suitable for metal binding^34^. Taking into account the localization of several Zn^2+^ transporters in the ESP and the putative metal-binding site of ERp44, we surmised that Zn^2+^ regulates the function of this chaperone.

Here, we demonstrate that indeed Zn^2+^ binds to ERp44 with submicromolar affinity, dynamically controlling its traffic and activity. The crystal structure of Zn^2+^-bound ERp44 has established the structural basis of the Zn^2+^-mediated functional regulation of ERp44. Our findings identify a Zn^2+^-dependent mechanism of protein quality control at the ER–Golgi interface, revealing yet another physiological role for this essential metal ion in the secretory pathway.

## Results

### Zn^2+^ binds to ERp44 to induce its conformational changes

**Fig. 1:**
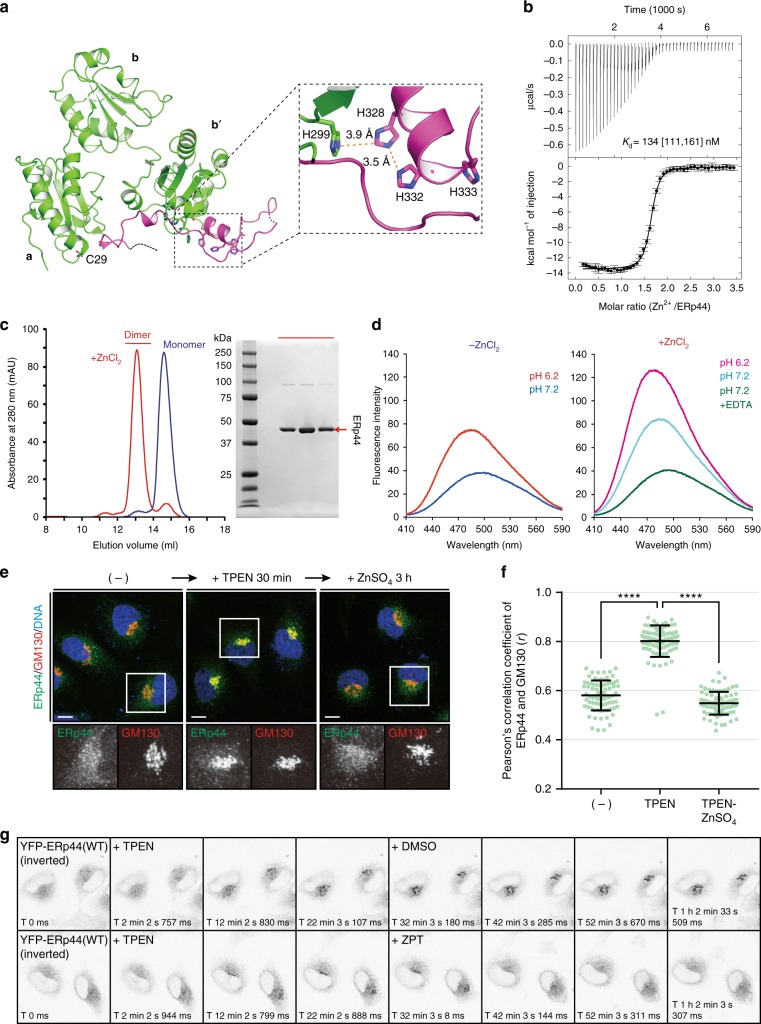
Zn^2+^ binds to ERp44, affecting its structure and localization. **a** Updated crystal structure of ERp44 at 2.0 Å resolution (PDB ID: 5GU6). The three Trx-like domains (**a**, **b**, and **b**′) and C-tail are shown in green and magenta, respectively. The inset shows a close-up view of the His-cluster composed of His299, His328, and His332. **b** ITC raw data (upper) and binding isotherm data (lower) for titration of ZnCl_2_ (500 µM) into ERp44 (30 µM) at pH 7.2. Bars represent the errors in the peak integration for each injection estimated by NITPIC^72^. The global analysis was performed to estimate the *K*_d_ and Δ*H* values with SEDPHAT^73^ assuming 1:1 binding. The apparent *K*_d_ is shown with 68.3% confidence interval in brackets. **c** SEC analysis of ERp44 (60 µM) in the presence (red line) or absence (blue line) of Zn^2+^ ions (120 µM). Peak fractions of the ERp44-ZnCl_2_ mixture eluted at 12.5–14.0 mL were analyzed by non-reducing SDS-PAGE (right), suggesting that major portion of ERp44 forms non-covalent homodimers in a Zn^2+^-dependent manner. **d** ANS fluorescence spectra were measured for ERp44 at pH 7.2 or 6.2 in the absence (left) or presence (right) of ZnCl_2_. The higher and blue-shifted fluorescence peaks observed at lower pH and those in the presence of Zn^2+^ suggest the exposure of hydrophobic surfaces in ERp44. Subsequent addition of EDTA to the ERp44-ZnCl_2_ mixture returned the fluorescence to the initial level. **e** Confocal immunofluorescence images showing the intercellular localization of endogenous ERp44 (in green) in HeLa cells. Cells were co-stained with an antibody to GM130 (in red) and with DAPI (in blue) to highlight the Golgi and nucleus, respectively. Note that ERp44 accumulates in the Golgi upon Zn^2+^ deprivation and returns to the ER upon Zn^2+^ replenishment. Scale bars, 10 µm. **f** Quantitative analyses of the Pearson’s correlation coefficients for the co-localization of endogenous ERp44 with GM130 based on the immunofluorescence images shown in **e**. Dots indicate individual data points (≥70 cells for each condition). Bars indicate the means ± SD. ****p < 0.0001. **g** Time-lapse fluorescence imaging of YFP-ERp44 in living cells. HeLa cells transfected with YFP-ERp44 were treated with TPEN for 30 min and then with ZPT or DMSO (vehicle) for 30 min. As described for endogenous ERp44, Zn^2+^ depletion and replenishment alters intracellular localization of YFP-ERp44 in a reversible manner

Considering that histidines can coordinate Zn^2+^, the presence of the highly conserved His-cluster at the regulatory C-tail (Fig. 1a) suggested that ERp44 binds Zn^2+^ via this cluster. To test this possibility, we first investigated whether and how ERp44 is able to bind Zn^2+^ by isothermal titration calorimetry (ITC) (Fig. 1b and Supplementary Fig. 1A). ITC analyses showed that ERp44 binds 1.5 molar equivalents of Zn^2+^ with apparent *K*_d_ values of 135–295 nM at pH 7.2–6.2. This stoichiometry suggests that two ERp44 molecules, each of which binds one Zn^2+^ at the His-cluster, coordinate another Zn^2+^ to form a homodimer. To confirm the Zn^2+^-dependent dimerization of ERp44, we performed size exclusion chromatography (SEC) analyses. When a mixture of ERp44 (60 µM) and ZnCl_2_ (120 µM) was loaded onto a SEC column, the majority of ERp44 was eluted as non-covalent homodimers (Fig. 1c) with a molecular mass of ~80 kDa, as determined by SEC combined with multi-angle light scattering (SEC-MALS) analyses (Supplementary Fig. 1C). The Zn^2+^-mediated dimerization was also observed within a pH-range of 6.2–7.2 (Supplementary Fig. 1B).

Next, we investigated the influence of Zn^2+^ binding on the structure of ERp44 by far-UV CD and fluorescence spectroscopy. The far-UV CD spectra were insensitive to Zn^2+^ addition (Supplementary Fig. 1C), indicating that the secondary structure was maintained in ERp44 regardless of Zn^2+^ availability. By contrast, fluorescence spectroscopy using 1-anilinonaphthalene-8-sulfonate (ANS) revealed that Zn^2+^ binding induced significant conformational changes in ERp44 (Fig. 1d). In fact, similarly to the pH change from 7.2 to 6.2^32^, addition of excess Zn^2+^ strikingly increased the fluorescence intensity of ANS at both pH 6.2 and pH 7.2 (Fig. 1d, compare right and left panels), implying increased exposure of hydrophobic surfaces upon Zn^2+^ binding to ERp44. Subsequent chelation of Zn^2+^ with EDTA fully abolished this effect, highlighting the reversibility of the Zn^2+^-dependent conformational changes of ERp44. These results unequivocally demonstrate that Zn^2+^ reversibly binds to ERp44 with submicromolar affinity, inducing significant conformational changes and dimerization.

### Zn^2+^ regulates the subcellular localization of ERp44

We previously showed that whereas wild-type ERp44 localizes in the ER–Golgi intermediate compartment (ERGIC) and *cis*-Golgi^28^, its mutants lacking one or all histidines of the conserved cluster can proceed further along the secretory pathway and be in part secreted out^37^. Thus, we surmised that Zn^2+^ binding to the His-cluster of ERp44 could influence its intracellular traffic. To test this hypothesis, we examined the effects of Zn^2+^ depletion and replenishment on the subcellular localization of ERp44 by immunofluorescence. In untreated HeLa cells, endogenous ERp44 co-localized mainly with markers of the ERGIC and *cis*-Golgi, but not of the early or late endosome, as observed previously^28^ (Fig. 1e, left panels and Supplementary Fig. 2). When cells were treated for 30 min with a membrane-permeable Zn^2+^ chelator, *N*,*N*,*N′*,*N′*-tetrakis (2-pyridylmethyl) ethylenediamine (TPEN), ERp44 accumulated in a perinuclear area, largely co-localizing with GM130, a *cis*-Golgi marker (Fig. 1e, middle panels, and 1 f). Subsequent addition of excess ZnSO_4_ was sufficient to re-localize endogenous ERp44 primarily to the ERGIC (Fig. 1e, right panels and 1f). Similar phenomena were observed for endogenous ERp44 in HepG2 cells (Supplementary Fig. 3A) and for YFP-tagged ERp44 (YFP-ERp44) overexpressed in HeLa cells (Supplementary Fig. 3B, C).

Time-lapse fluorescence imaging allowed to dynamically visualize the Zn^2+^-dependent movements of ERp44 in living cells (Fig. 1g, Supplementary Movies 1 and 2). After 30 min in the presence of TPEN, YFP-ERp44 accumulated strikingly in a perinuclear GM130-positive area, and returned to the ER within 15 min after addition of zinc pyrithione (ZPT; 20 µM), a Zn^2+^-specific ionophore (Fig. 1g, lower panel, Supplementary Movies 1 and 2). The overall structure of the Golgi was not significantly affected by short-time treatments with TPEN and ZPT (Supplementary Fig. 2B, lower panels), underlining the specificity of the phenomena observed for ERp44. Additionally, DMSO, a reagent used to dissolve TPEN and ZPT, hardly altered ERp44 localization (Fig. 1g, upper panel; Supplementary Movie 2). Taken together, the above results demonstrate that Zn^2+^ dynamically and reversibly controls the intracellular traffic of ERp44.

### Zn^2+^ at physiological levels controls ERp44 traffic and activity

**Fig. 2:**
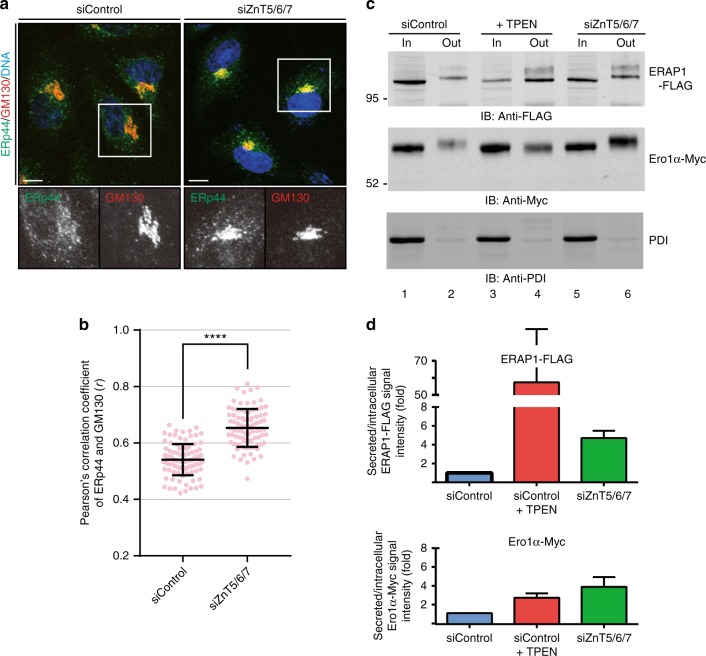
Knockdown of the Golgi-resident Zn^2+^ transporters alters ERp44 localization and function in cells. **a** Confocal immunofluorescence images showing the intracellular localization of ERp44 after ZnT5, 6 and 7 triple knockdown (siZnT5/6/7) or treatment with control siRNA (siControl). Cells were co-stained with antibodies specific for ERp44 (in green), GM130 (in red) and DAPI (in blue). Scale bar, 10 µm. The simultaneous knockdown of ZnT5/6/7 caused accumulation of ERp44 in the Golgi, similarly to TPEN (Fig. 1e). **b** Quantitative analysis of Pearson’s correlation coefficients of the co-localization of endogenous ERp44 with GM130 based on the immunofluorescence images shown in **a**. Dots indicate individual data points (≥80 cells for each condition, unpaired *t*-test). Bars indicate the means ± SD. ****p < 0.0001. **c**, **d** TPEN and ZnT5/6/7 triple knockdown promote secretion of two ERp44 clients, Ero1α and ERAP1, but not of PDI. Lysates of HeLa transfectants (In) and TCA-precipitated culture supernatants (Out) were resolved by SDS-PAGE under reducing conditions and blot membranes were decorated with anti-FLAG, anti-Myc, and anti-PDI antibodies to visualize ERAP1, Ero1α, and PDI, respectively. To quantify retention efficiency, the ratios between the band intensity of the secreted and intracellular ERAP1 and Ero1α were calculated and expressed as fold of induction relative to untreated cells (**d**). Bars indicate the means ± SEM of five independent experiments. Clearly, depleting Zn^2+^ from the ESP by TPEN or ZnT5/6/7 triple knockdown allowed secretion of ERAP1 and Ero1α. Note that secreted ERAP1 and Ero1α displayed slower gel electrophoretic mobility than intracellular ones, possibly due to further processing of *N*-glycans and/or *O*-glycosylation in the Golgi. No band shifts were observed for TPEN treatment, which may be explained by TPEN-triggered inactivation of Golgi-resident metal-dependent galactosyltransferases and/or glycosyltransferases

To further investigate the physiological role of Zn^2+^ in ERp44 regulation, we silenced the Golgi-resident transporters ZnT5, ZnT6, and ZnT7. Their knockdown was recently reported to cause secretion of inactive apo-Zn^2+^-enzymes, in line with their pivotal roles in Zn^2+^ uptake into the ESP^19^. Upon ZnT5/6/7 triple knockdown (Supplementary Fig. 4E), endogenous ERp44 co-localized with GM130 (Fig. 2a, b), as observed in TPEN-treated cells (Fig. 1e, Supplementary Fig. 2A, B). Similarly, endogenous ERp44 in HepG2 cells (Supplementary Fig. 4A, B and G) and overexpressed YFP-ERp44 in HeLa cells (Supplementary Fig. 4C and D) moved to the *cis*-Golgi upon ZnT5/6/7 silencing. Thus, ERp44 predominantly accumulates in the Golgi upon ZnT5/6/7 triple knockdown, indicating that physiological levels of Zn^2+^ in the ESP regulate its subcellular localization.

Next, we examined the effects of Zn^2+^ depletion on the ability of ERp44 to retain overexpressed ERAP1 and Ero1α, two enzymes that normally reside in the ER despite the lack of a KDEL-motif^20,25,27^. Compared to controls, TPEN treatment enhanced secretion of these two clients (Fig. 2c, lanes 1–4). Importantly, ZnT5/6/7 triple knockdown yielded similar results (Fig. 2c, lanes 5 and 6, Supplementary Fig. 4F), strongly suggesting that Zn^2+^ imported into post-ER compartments via one or more Zn^2+^ transporter(s) have an essential role in efficient client retention by ERp44. In contrast, the ER-resident chaperone PDI was not secreted, confirming the specificity of the effects of Zn^2+^ deprivation on ERp44 (Fig. 2c, bottom). The consequences of Zn^2+^ deprivation were more evident for ERAP1 than for Ero1α (Fig. 2d), probably because Ero1α is also retained in the ER by interactions with its redox partner PDI^22,24,38,39^. Accordingly, the subcellular localization of Ero1α molecules that were not secreted was unaltered by Zn^2+^ depletion and replenishment (Supplementary Fig. 4H). Moreover, we observed that Ero1α itself has no ability to bind Zn^2+^ (Supplementary Fig. 4I).

To confirm the specific binding of ERp44 to Zn^2+^, we tested the ability of ERp44 to bind copper (Cu^2+^) and manganese (Mn^2+^) ions, because copper transporters and manganese transporters are known to be localized in the secretory pathway^40,41^. The ITC analysis showed that ERp44 binds Cu^2+^ ions, but with micromolar affinity (*K*_d_ = 1.65 µM) (Supplementary Fig. 5A). No interactions were detected between ERp44 and Mn^2+^ (Supplementary Fig. 5B). Notably, copper chelation by tetraethylenepentamine little affected the subcellular localization of ERp44 (Supplementary Fig. 5C–E), suggesting that copper is not involved in ERp44 regulation. Altogether, we conclude that Zn^2+^ at physiological levels specifically controls the cellular traffic and client retention activity of ERp44.

### Structure of the Zn^2+^-bound form of ERp44

**Fig. 3:**
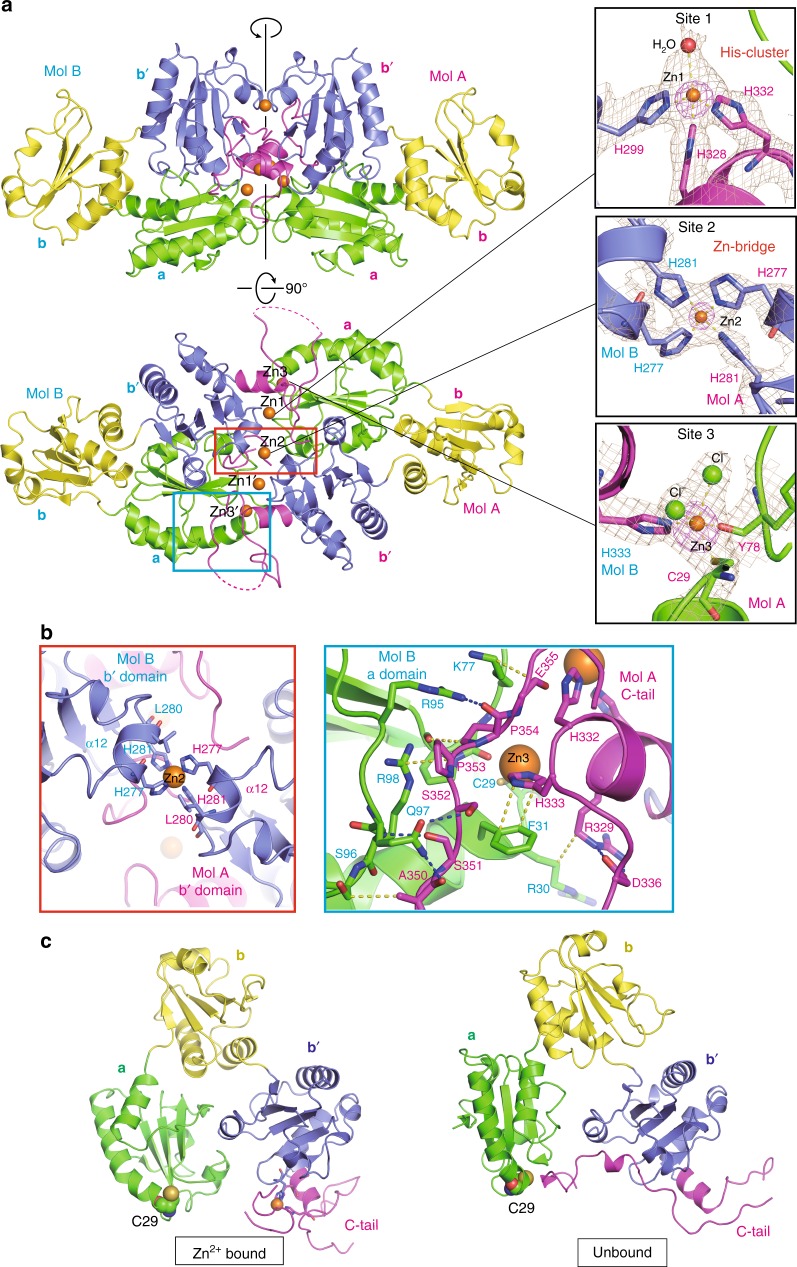
Structure of Zn^2+^-bound form of ERp44. **a** Top and side view of the overall structure of the Zn^2+^-bound dimer of ERp44. The **a**, **b**, **b**′ domains and C-tail of Mol A and Mol B are shown in green, yellow, blue and magenta, respectively. The Zn^2+^ ions are represented by orange spheres. A vertical black line represents a non-crystallographic twofold axis. The right insets display the close-up views of the three Zn^2+^ binding sites: site 1 (top), site 2 (middle) and site 3 (bottom). Simulated annealing 2Fo−Fc omit maps at 1–1.3*σ* and anomalous difference Fourier map at 15*σ* are shown in brown and magenta, respectively. **b** Close-up views of the dimer interfaces; (left): highlighted view of the red box in **a**, which illustrates interactions formed between the α12 helices of the **b**′ domains in ERp44 dimer; (right): highlighted view of the blue box in **a**, which illustrates interactions formed between the C-tail of Mol A and the **a** domain of Mol B. Hydrogen bonds and van der Waals contacts are shown by blue and yellow dashed lines, respectively. **c** Comparison of the overall structure of the Zn^2+^-bound (left) and unbound (right) forms of the ERp44 protomer. The essential cysteine (Cys29) is shown as spheres

To reveal how Zn^2+^ impacts ERp44 conformation at atomic resolution, we determined the crystal structure of the ERp44–Zn^2+^ complex at 2.45 Å resolution by the single anomalous dispersion (SAD) method using the anomalous signals of Zn^2+^ (Supplementary Fig. 6A, B, and Supplementary Tables 5 and 6). The crystallographic asymmetric unit contains four ERp44 molecules, which form two homodimers (dimer 1: Mol A and Mol B; dimer 2: Mol C and Mol D) (Supplementary Fig. 6A). The overall architecture of the Zn^2+^-bound ERp44 homodimer resembles two clover leafs aligned tail to tail (Fig. 3a). The two ERp44 monomers in the homodimer are nearly related by a twofold axis (Fig. 3a top, a vertical line). At the center of the dimer, the α12 helices in the **b**′ domains are bridged by a Zn^2+^ ion (Fig. 3b, left). The Zn^2+^-bridged non-covalent homodimer is consistent with the results of the ITC and SEC analyses (Fig. 1b, c). Within the homodimer, larger dimer interfaces are also formed between a part of the C-tail of one protomer and the **a** domain of the other protomer, including a *π*–*π* stacking interaction between His333 (Mol A) and Phe31 (Mol B), an arginine stacking interaction between Arg329 (Mol A) and Arg30 (Mol B) and several hydrogen bonds and van der Waals contacts between the C-tail segment (residues Ala350–Glu356) in Mol A and a part of the **a** domain (residues Lys77 and Arg95 to Arg98) in Mol B (Fig. 3b, right).

Unlike metal-free ERp44, the Zn^2+^-bound ERp44 monomer adopts an open conformation in which the C-tail is released from the **a** domain and the client-binding surface including Cys29 is exposed to the solvent (Fig. 3c). By contrast, the C-tail is closed to mask Cys29 and its neighboring region in metal-unbound ERp44 (Fig. 3c, right)^34^. The C-terminal region (residues 359–378) of each protomer in the Zn^2+^-bound homodimer shows very high B-factors, adopting different conformations (Supplementary Fig. 6C, D). Residues 366–377 of Mol A insert into the interior of the dimer interface (Supplementary Fig. 6E), whereas the residues 360–366 of Mol B extend toward outside the molecule (Supplementary Fig. 6F). The corresponding regions of Mol C and Mol D are more disordered (Supplementary Fig. 6D). Thus, the C-terminal region seems to partially stabilize the dimeric structure, but its contribution may vary in different protomers.

### Three Zn^2+^-binding sites in ERp44

The present structure reveals three types of Zn^2+^ binding sites in the ERp44 homodimer (sites 1, 2, and 3), with a total of five Zn^2+^ ions aligned close to the dimer interface (Fig. 3a, right panels). Site 1 is formed by the His-cluster in each protomer. Zn^2+^ (Zn1 and Zn1′) is coordinated by three histidines (His299, His328 and His332) and a water molecule in a tetrahedral geometry (Fig. 3a, right top) (Supplementary Table 8). This mode of Zn^2+^ coordination is widely found in other Zn^2+^-binding proteins such as carbonic anhydrase II, insulin hexamers, and the serine protease tonine^5,42,43^. Site 1 is also stabilized by van der Waals contacts with several surrounding residues from the adjacent protomer in the Zn^2+^-bound homodimer (Supplementary Fig. 6G).

Site 2 is formed at the center of the dimer interface, contributing directly to homodimerization of ERp44. Two antiparallel histidine pairs (His277 and His288) in the mutually adjacent **b**′ domains coordinate a Zn^2+^ (Zn2) in a tetrahedral geometry (Fig. 3a, right middle) (Supplementary Table 8). Such intermolecular Zn^2+^ coordination by four histidines is found in a couple of proteins including the heme-responsive zinc-finger transcription factor HAP1^44^ and the RNA-binding anti-termination protein HutP^45^.

Site 3 is formed at both the ends of the dimer interface (Fig. 3a, right bottom). Zn^2+^ at site 3 (Zn3) is coordinated by the Nδ of His333 of one protomer, the thiol group of Cys29 and the main-chain carbonyl oxygen of Tyr74 of the other protomer and two chloride ions (see also Methods) (Supplementary Table 8). To our knowledge, the trigonal bipyramid geometry of site 3 has not been found so far in any metalloproteins of known structure^46^. Indeed, carbonyl oxygen is a minor ligand for coordination of Zn^2+^^4,47^. The presence of site 3 was unexpected, since our ITC analyses did not detect a third Zn^2+^ binding (Fig. 1b). The observation that the one-to-two mixture of ERp44 and Zn^2+^ eluted mostly as a Zn^2+^-bridged homodimer in the SEC analysis (Fig. 1c) suggests that Zn^2+^ binding to site 3 is not necessary for dimerization (see also the last paragraph of the next section).

### Sequential Zn^2+^ binding to ERp44

To elucidate the molecular details of Zn^2+^-binding to ERp44, we constructed two kinds of His mutants in which either set of His277 and His281 (H277/281A), or His299, His328, and His332 (3HA, previously called ABC^37^) were replaced by Ala. Far-UV CD measurements indicated that both mutants maintained the overall secondary structure, independently of Zn^2+^ (Supplementary Fig. 7A, B). As expected, the H277/281A mutant, which lacks site 2, did not form Zn^2+^-dependent homodimers (Supplementary Fig. 7C, left). ITC analyses revealed that H277/281A bound one molar equivalent of Zn^2+^ (Supplementary Fig. 7D, left), which most likely reflects Zn^2+^ binding to site 1 only. In contrast, neither Zn^2+^-dependent dimerization nor large enthalpy changes were observed for the 3HA mutant, which retains site 2 but lacks the His-cluster (site 1) (Supplementary Fig. 7C, D, right). These results suggest that Zn^2+^ binding to site 1 is required for Zn^2+^ binding to site 2 and hence, for the formation of the Zn^2+^-bridged homodimer.

To further analyze Zn^2+^ binding to these two sites, we calculated the Scatchard plots based on the ITC data (Supplementary Fig. 8A). Due to the apparent single-phase ITC profiles (Fig. 1b and Supplementary Fig. 1A), we could not determine the thermodynamic parameters for Zn^2+^ binding to the individual sites separately. However, the calculated Scatchard plots displayed concave downward curves at all pH measured (Supplementary Fig. 8A), a feature characteristic of multiple ligand binding with ‘positive cooperativity’^48,49^. The Hill coefficients estimated from the saturation curve based on the ITC data were significantly higher than 1 (Supplementary Fig. 8B), supporting the positive cooperativity in sequential Zn^2+^ binding to ERp44. This means that the first Zn^2+^ binding to site 1 significantly increases the affinity for Zn^2+^ of site 2, facilitating the formation of the Zn^2+^-bridged dimer. In line with this, our SEC analyses demonstrated that significant amounts of Zn^2+^-mediated ERp44 dimers were generated even at ERp44/Zn^2+^ ratios of 1:0.5 to 1:1, before the saturation of site 1 with Zn^2+^ (Supplementary Fig. 8C). In this context, dimerization appears to stabilize the Zn^2+^-binding configuration of site 1 via interactions with Trp28 and Ser76 of the other protomer (Supplementary Fig. 4G). To estimate the dissociation constant for the Zn^2+^-bridged dimer, we also performed the dissociation titration experiments (Supplementary Fig. 8D). However, no significant heat changes were observed during the dilution titration. Thus, the Zn^2+^-mediated ERp44 dimer hardly disassembles at a concentration of 0.1 µM (100-fold dilution of titrant), implying that the dissociation constant for ERp44 dimerization is below 0.1 µM.

We also constructed a His333/Ala mutant (H333A) to investigate the role of site 3 (Supplementary Fig. 9). In the Zn^2+^-bridged dimer, His333 not only coordinates Zn3, but also forms a *π*–*π* stacking interaction with Phe31, seemingly contributing to stabilization of the dimer. In agreement with this, the SEC analysis showed that H333A was eluted only slightly faster in the presence of ZnCl_2_, suggesting that this mutant did not form a stable homodimer with Zn^2+^ (Supplementary Fig. 9A). ITC analysis showed that H333A bound one Zn^2+^ per monomer, with much lower affinity (*K*_d_ = 2.49 µM) than WT and H277/281/A (2HA) (Supplementary Fig. 9B). This may be explained by the likely perturbation of H333A mutation on the local conformation at site 1 involving His328 and His332. Thus, His333 seems to have auxiliary roles in Zn^2+^ binding to ERp44 and hence dimer formation.

### Zn^2+^ binding induces striking conformational changes of ERp44

**Fig. 4:**
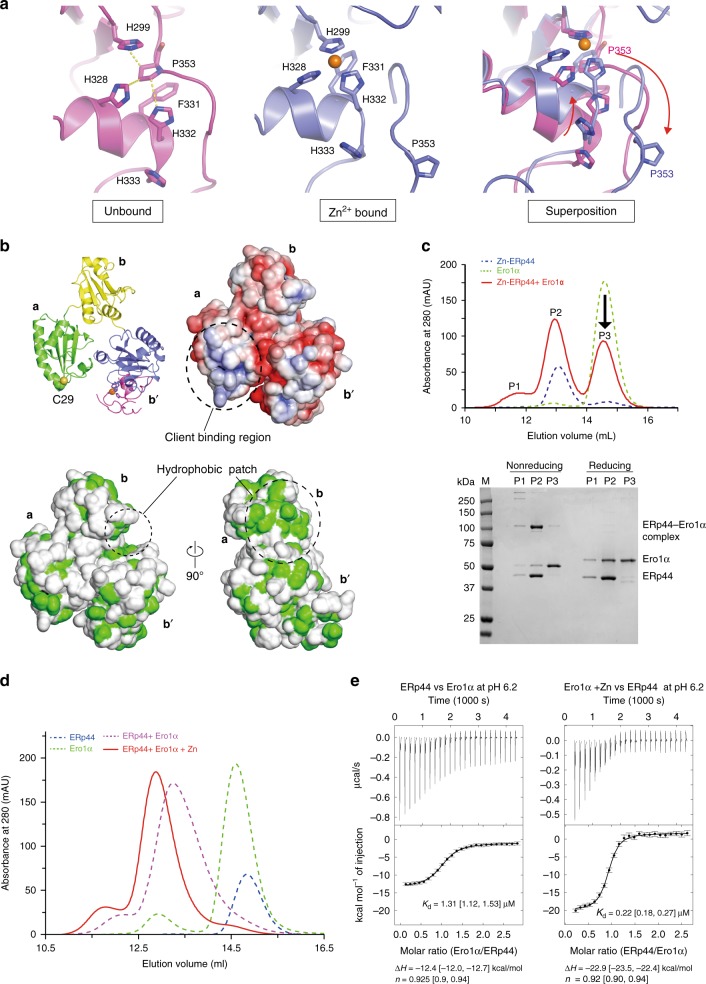
Zn^2+^-dependent conformational changes enhance the ERp44–Ero1α interaction. **a** Comparison of the His-cluster (site 1) conformation in the unbound (left), Zn^2+^-bound states (center), and their superposition (right). Yellow dashed lines in the right panel indicate van der Waals contact among the three histidine and Pro353. **b** Molecular surface properties of the Zn^2+^-bound ERp44. (Upper left) Ribbon representation as a reference; (upper right) Electrostatic surface potential, in which positively and negatively charged regions are shown in blue and red, respectively. (Lower) Hydrophobic regions viewed from two different angles, in which hydrophobic residues (except main-chain oxygen and nitrogen atoms) are shown in green. **c** SEC analysis for the mixture of ERp44 (40 µM) and ZnCl_2_ (80 µM) (dashed blue line), Ero1α (40 µM) alone (dashed green line), and the pre-mixture of ERp44 (40 µM) and ZnCl_2_ (80 µM) supplemented with Ero1α (40 µM) (red line) at pH 6.2. Peak fractions were analyzed by SDS-PAGE under non-reducing or reducing conditions. **d** SEC analysis of ERp44 (40 µM), Ero1α (40 µM), and equimolar mixtures of ERp44 and Ero1α (40 µM each) with or without Zn^2+^ ions (80 µM) at pH 6.2. **e** ITC raw data (upper) and binding isotherm data (lower) for titration of Ero1α into ERp44 at pH 6.2 (left) or titration of ERp44 into a mixture of Ero1α and Zn^2+^ at pH 6.2 (right). The calculated *K*_d_ is shown with 68.3% confidence interval in brackets

The above crystallographic studies further reveal that Zn^2+^ binding to site 1 induces striking movements of the C-tail, accompanied by significant rearrangements of the Trx-like domains (Supplementary Movie 3). In the metal-free state, Pro353 stacks with Phe331 and makes van der Waals contacts with His328, His299 and His332, intervening between these three histidines (Fig. 4a, left). Such separate location of the histidines involved does not seem optimal for Zn^2+^ binding. In the Zn^2+^-bound state, however, Pro353 is largely moved out and the α16 helix gets closer to His299 (Fig. 4a, middle and right). As a result, His299, His328, and His332 adopt a configuration suitable for Zn^2+^ binding (site 1). Noteworthy, the large movement of Pro353 accompanies a striking C-tail movement toward the **b**′ domain, disrupting the interactions between the C-tail and the **a** domain (Fig. 3c). In this state, Cys29 becomes fully exposed to the solvent (Figs. 3c, 4b), and the **a** and **b** domains are rotated by ~77˚ with respect to the **b**′ domain (Supplementary Fig. 10A).

The Zn^2+^-dependent conformational changes also influence the molecular surface properties of ERp44. In the **a** domain, the surroundings of Cys29 are found to be positively charged to a significant extent (Fig. 4b, upper). The surface features of Zn^2+^-bound ERp44 protomer are similar to those observed in ERp44 at weakly acidic pH^34^. Additionally, a large hydrophobic patch between the **b** and **b**′ domains becomes more exposed to the solvent in the Zn^2+^-bound state (Fig. 4b, lower and Supplementary Fig. 10B). This hydrophobic patch is not involved in the dimer interface, which accounts for the enhanced ANS fluorescence after Zn^2+^ addition (Fig. 1d). These structural insights suggest that the Zn^2+^-bound form of ERp44 recognizes its client proteins with higher affinity than the metal-free form.

### Zn^2+^ enhances the interactions between ERp44 monomers and Ero1α

To investigate how Zn^2+^ affects ERp44–client interactions, we performed SEC and ITC analyses for the ERp44–Ero1α association in the presence or absence of the metal ion. To this end, we first added Ero1α to the Zn^2+^-bound ERp44 homodimers and subsequently applied the mixture to a SEC column. Clearly, whereas a minor portion of the proteins formed higher disulfide-linked oligomers (P1 fraction), a major portion of Ero1α co-eluted with ERp44 as both disulfide-linked and non-covalent complexes (P2 fraction in Fig. 4c). The P2 fraction also contained the remainder of ERp44. The molecular mass of the P2 fraction was estimated to be ~90 kDa by SEC-MALS analysis (Supplementary Fig. 11), suggesting that this fraction contains both ERp44–Ero1α heterodimers and Zn^2+^-mediated ERp44 homodimers. Thus, in the presence of Ero1α, significant portion of the Zn^2+^-bound ERp44 homodimer dissociates to form the ERp44–Ero1α complexes.

When a pre-mixture of Ero1α (40 µM) and Zn^2+^ (80 µM) was incubated with ERp44 (40 µM) and then applied to the column, the ERp44–Ero1α binary complex was formed predominantly (Fig. 4d, red line), indicating that Zn^2+^-bound ERp44 monomers preferentially bind Ero1α rather than forming Zn^2+^-bridged homodimers. In the presence of Zn^2+^, the binary complex eluted as a narrower peak with a slightly smaller elution volume (Fig. 4d, red and purple broken lines), suggesting that the interactions between ERp44 and Ero1α are enhanced by Zn^2+^. In support of this, the ITC analysis showed that Zn^2+^-dependent formation of the Ero1α–ERp44 complex produced larger enthalpy changes with sixfold higher affinity (*K*_d_ = 0.22 µM), compared to the Zn^2+^-independent association (*K*_d_ = 1.31 µM) (Fig. 4e). Accordingly, TPEN treatment significantly reduced the interactions between exogenously expressed Ero1α-Myc and YFP-ERp44 in cells, and the subsequent ZPT treatment restored their complex formation (Supplementary Fig. 12A). Endogenous ERp44 also formed disulfide-linked binary and higher molecular weight complexes with Ero1α in cells, which were resolved by TPEN treatment (Supplementary Fig. 12B). Thus, Zn^2+^ significantly enhances the interaction between ERp44 and Ero1α.

To test whether ERp44 homodimerization competes with client binding also in living cells, we monitored the association of FLAG-ERp44 and YFP-ERp44 in HeLa transfectants expressing increasing amounts of Ero1α, with or without ZPT supplementation. The amount of FLAG-ERp44 co-precipitated with YFP-ERp44 was significantly decreased as the levels of Ero1α increased (Supplementary Fig. 12C). The effect of Ero1α co-expression was more striking when the cells were treated with ZPT. Concomitantly, ERp44–Ero1α complexes were generated to a greater extent. Altogether, the data support that—both in vitro and in vivo—ERp44 binds Ero1α with higher affinity than another ERp44 molecule and that Zn^2+^ further promotes the association between ERp44 and Ero1α.

### Occurrence of Zn^2+^-mediated ERp44 homodimers in cells

The formation of Zn^2+^-dependent ERp44 homodimers was further explored by exploiting HepG2 cells that express Halo-tagged ERp44 variants (Supplementary Fig. 13A). Lysates were precipitated with Halo ligand-beads, which covalently bind the Halo domain of the chimeric proteins. After careful washing, beads were sequentially eluted with TPEN and then with DTT, to dissect non-covalent/Zn^2+^-dependent and covalent interactions. TPEN treatments eluted both Halo-tagged and endogenous ERp44 molecules from beads covered with Halo-ERp44 WT, but not from beads covered with Halo-ERp44 3HA (Supplementary Fig. 13B). Similarly, when HepG2 cells coexpressed different tagged versions of WT ERp44 (Halo or HA), both were eluted from the beads by TPEN (Supplementary Fig. 13C). The association between HA-ERp44 WT and Halo-ERp44 WT was further enhanced by addition of ZnCl_2_ to cell lysates. (Supplementary Fig. 13C, lanes 8 and 17). Thus, Zn^2+^ binding is necessary to form and stabilize non-covalent homodimers also in vivo. In addition, a fraction of ERp44 WT and 3HA that formed covalent oligomers^27,50^ were eluted by treating the beads with DTT (Supplementary Fig. 13A, lanes 14 and 15). Altogether, the results indicate that ERp44 WT, but not 3HA, forms Zn^2+^-mediated non-covalent dimers (or higher oligomers) in cells.

The occurrence of the Zn^2+^-bridged ERp44 homodimers including endogenous ERp44 is further supported by SEC analyses of the microsomal fraction (Supplementary Fig. 13D). In addition to monomer fractions (eluted at ~14.5 mL), a significant portion of endogenous ERp44 was eluted at ~12.5 mL (Supplementary Fig. 13E, left). Of note, the elution peak at ~12.5 mL corresponds to that of the Zn^2+^-bridged ERp44 homodimers (Fig. 1c) and diminished upon TPEN treatment, but not upon tris(2-carboxyethyl)phosphine (TCEP) treatment (Supplementary Fig. 13E, middle and right). Meanwhile, TCEP markedly reduced most of the covalent complexes including ERp44 seen at 12.0–13.5 mL. Although we cannot exclude the possibility that the earlier peak at ~12.5 mL contains some ERp44 molecules non-covalently bound to client proteins of a molecular size similar to ERp44, these SEC analyses, taken together with the sequential elution assays shown above, indicate that ERp44 can certainly form Zn^2+^-dependent non-covalent and covalent homodimers in cells.

### Zn^2+^-regulated subcellular localization and function of ERp44

**Fig. 5:**
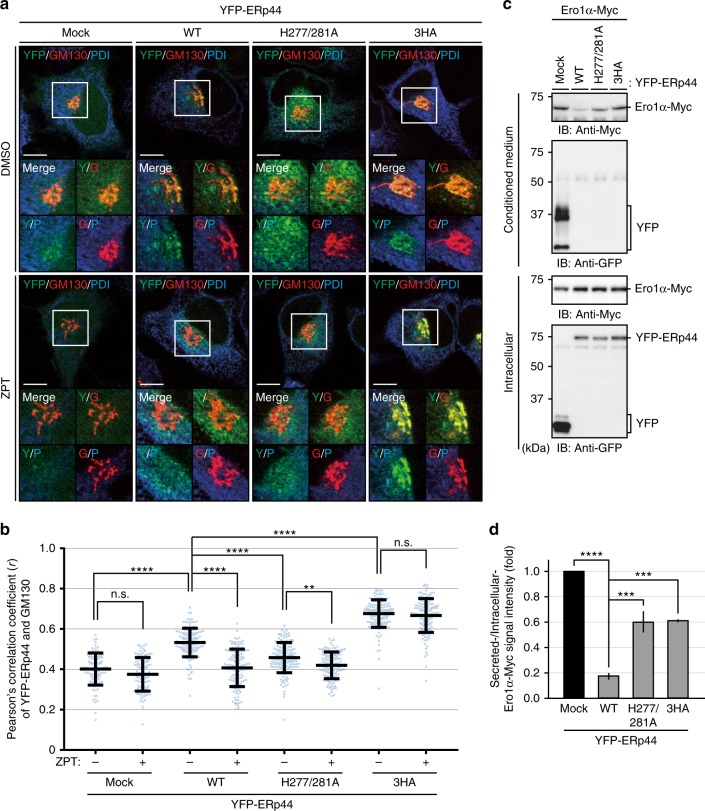
Client-retention activity of ERp44 is modulated by its subcellular localization. **a** Confocal immunofluorescence images showing the subcellular localization of YFP-ERp44 WT, H277/281A, and 3HA in HeLa cells. HeLa cells transfected with YFP-Mock or YFP-ERp44 (WT, H277/281A, or 3HA) were treated with DMSO or 2.5 µM ZPT for 15 min and immunostained for GM130 (*cis*-Golgi marker, in red) and PDI (ER marker, in blue). Note that YFP-ERp44 (H277/281A) and (3HA) preferentially localized to the ER and Golgi, respectively. Scale bars, 10 µm; insets, magnification. **b** Quantitative analyses of Pearson’s correlation coefficients of co-localization of YFP-signals with GM130 staining. Dots indicate individual data points (>100 cells from three independent experiments, one-way ANOVA followed by Tukey’s test). Bars indicate the means ± SD. ****p < 0.0001; **p < 0.01; n.s. not significant (*p* > 0.05). **c**, **d** Mutations on histidines involved in Zn^2+^-binding inhibit Ero1α retention activity of ERp44. HeLa cells transfected with Ero1α-Myc and YFP-Mock or YFP-ERp44 (WT, H277/281A, or 3HA) were incubated in serum-free Opti-MEM for 6 h. Conditioned media were precipitated with TCA and analyzed by immunoblotting with anti-GFP or anti-Myc antibodies, as indicated, under reducing conditions. The signal intensity of secreted Ero1α-Myc relative to that of intracellular Ero1α-Myc was quantified, and the data are shown in the graph (**d**). Data are the means ± SEM (*N* = 3, one-way ANOVA followed by Dunnett’s test). ****p < 0.0001; ***p < 0.001

To clarify the correlation between the Zn^2+^-binding ability and subcellular localization of ERp44, we performed cell imaging analyses for the YFP-ERp44 H277/281A (YFP-H277/281A) and 3HA (YFP-3HA) mutants. Due to the lack of site 1, 3HA mutant is predicted not to readily release the C-tail in the presence of Zn^2+^, leading to defective recognition of the C-terminal RDEL sequence by KDELRs and hence more distal localization with respect to WT (and likely partial secretion). In agreement with our previous results^35^, disruption of the His-cluster (site 1) led to extensive co-localization of the mutant YFP-3HA with the *cis*-Golgi marker GM130 (Fig. 5a, b), mimicking the phenotype observed with ERp44 WT in TPEN-treated cells (Fig. 1e and Supplementary Fig. 3). Moreover, as predicted for ERp44 mutants incapable of binding Zn^2+^, ZPT treatment did not affect the localization pattern of YFP-3HA (Fig. 5a, b). In line with these findings, the cells expressing YFP-3HA secreted a much larger amount of Ero1α-Myc than those expressing YFP-ERp44 WT (Fig. 5c, d). These results suggest that Zn^2+^ binding to the His-cluster (site 1) is critical for both recognition by KDELRs and efficient retrograde transport of client proteins.

In sharp contrast to YFP-3HA, YFP-H277/281A, which is able to bind equimolar Zn^2+^ at site 1 but unable to dimerize due to the lack of site 2, preferentially accumulated in the ER. Due to its inability to form Zn^2+^-bridged dimers, the Zn^2+^-bound form of YFP-H277/281A is predicted to expose its C-terminal RDEL motif to the solvent constitutively, without concealing it inside the dimer interface. However, this mutant accumulated in the Golgi upon TPEN treatment, as observed with YFP-WT (Supplementary Fig. 14), suggesting that it can potentially be exported from the ER to the post-ER compartments. Though still exhibiting Zn^2+^-dependent subcellular localization changes, H277/281 A was less efficient in retaining Ero1α-Myc than WT (Fig. 5c, d). These results suggest that proper cycling of ERp44 in post-ER stations is crucial for efficient Ero1α retention. In agreement with this notion, we previously reported that ERp44 mutants of which the C-tail is constitutively open reside predominantly in the ER and retain poorly their clients^20,32^.

## Discussion

The present studies reveal that Zn^2+^ regulates the structure, function and subcellular localization of ERp44, providing a Zn^2+^-dependent mechanism regulating protein quality control in the ESP. Zn^2+^ binding to the His-cluster (site 1) induces striking structural rearrangement in ERp44, increasing sixfold its affinity for the substrate, Ero1α (Fig. 4e). Upon the Zn^2+^-induced release of the C-tail, the C-terminal RDEL motif is fully exposed to the solvent. The  motif can hence be readily recognized by KDELR at the Golgi, and ERp44 is transported to the ER with high efficiency. Thus, excess Zn^2+^ localizes ERp44 predominantly in the ER. By contrast, Zn^2+^ chelation prevents the exposure of the RDEL motif by keeping the C-tail closed. ERp44 in this conformation will hardly be recognized by KDELR. As a consequence, ERp44 accumulates in the Golgi and is in part secreted. The observation that knockdown of the Golgi-resident Zn^2+^ transporters (ZnT5, ZnT6, and ZnT7) altered the ERp44 localization and compromised its client retention activity highlights the essential role of Zn^2+^ in the physiological regulation of ERp44. Like metallothioneins, ERp44 condenses diverse Zn^2+^-binding modes with different affinities and functional roles in a single protein. Although catalytic or structural Zn^2+^ binding sites in Zn^2+^ metalloproteins have picomolar to nanomolar affinity for Zn^2+^^5^, ERp44 binds Zn^2+^ with submicromolar affinity. Thus, Zn^2+^ likely has regulatory rather than structural roles for ERp44.

ERp44 has been found to possess three Zn^2+^-binding sites (sites 1, 2, and 3). Slightly lower Zn^2+^-binding affinity at lower pH can be explained by higher-degree protonation of the histidines at lower pH. Importantly, positive cooperativity exists in Zn^2+^-binding to sites 1 and 2 (Fig. 4a, b and Supplementary Fig. 6A); Zn^2+^ first binds to the conserved His-cluster (site 1), inducing large conformational changes, followed by facilitated Zn^2+^ binding to site 2 to form Zn^2+^-mediated homodimers. In line with this, superposition of metal-free ERp44 onto one protomer in the Zn^2+^-bound homodimer shows that the **a** domain in the metal-free state undergoes steric hindrance with the C-tail of another protomer in the Zn^2+^-bound state (Supplementary Fig. 15). To avoid this steric hindrance, significant domain rearrangements are induced by Zn^2+^ binding, leading to Zn^2+^-bridged dimerization. Site 3 has much lower Zn^2+^ affinity than the other two sites (Fig. 1b, c and Supplementary Fig. 8C). Nonetheless, mutating site-3 histidine (His333) weakened the Ero1α retention activity of ERp44^37^. The His333 mutation likely disrupts the dimer interface (Fig. 3b, right, Supplementary Fig. 7), thereby influencing the configurations of sites 1 and 2 and compromising the overall responsiveness of ERp44 to Zn^2+^. In the ESP, the potential binding of five Zn^2+^ atoms may enable ERp44 homodimers to function as Zn^2+^ buffer/storage devices, in analogy to cytoplasmic metallothioneins that bind up to seven metal ions^12,51^.

**Fig. 6:**
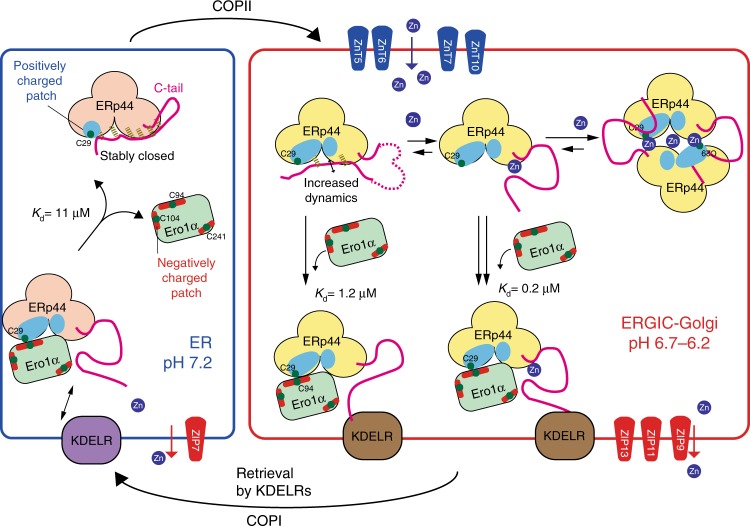
Zn^2+^-dependent and pH-dependent regulation of ERp44 for efficient client retention in the early secretory pathway. Proposed working model of the Zn^2+^-dependent and pH-dependent ERp44 cycle operating for protein quality control in the ESP. In the ERGIC and *cis*-Golgi, ERp44 is in equilibrium between monomeric metal-free, monomeric Zn^2+^-bound, and Zn^2+^-mediated homodimeric conformations. Upon the arrival of a client like Ero1α, the equilibrium is shifted in favor of the monomeric state with the C-tail opened, favoring the exposure of the RDEL motif. Thus, the ERp44–client complex is promptly bound by KDELR and therewith transported back to the ER. The lower Zn^2+^ concentration in the neutral ER likely promotes the dissociation of the ERp44–client complexes, allowing another ERp44 cycle through the ESP

The present findings allow us to propose a model of Zn^2+^-dependent protein quality control in the ESP (Fig. 6). At low Zn^2+^ concentration and neutral pH as in the ER, ERp44 likely assumes a closed conformation, with its client-binding surface masked by the C-tail^32,34^. At the weakly acidic pH of the *cis*-Golgi, ERp44 undergoes pH-dependent conformational changes that increase the C-tail dynamics and Cys29 reactivity and expose positively charged regions around the client-binding site^32,34^. Zn^2+^ binding further releases the C-tail, enhancing the interactions with clients and KDELRs. In the absence of clients, ERp44 is likely in equilibrium between a metal-free monomer, a Zn^2+^-bound monomer and a Zn^2+^-bridged homodimer in post-ER compartments (Fig. 6). Once the clients arrive at the Golgi, the Zn^2+^-bound ERp44 homodimers can dissociate to monomers to form binary complexes with clients. The ERp44–client complex is in turn retrieved to the ER in concert with KDELRs. In this scenario, the Zn^2+^-bridged ERp44 homodimer can be regarded as reservoirs that are ready to tightly bind clients. The dimer may also serve to store the metal in the ESP under Zn^2+^-limiting conditions.

How does Zn^2+^ participate in the ERp44 cycle under physiological settings? In mammalian cells, the total Zn^2+^ concentration including the protein-bound fraction is estimated to be 100–500 µM^15,52^. Recent X-ray fluorescence microscopy studies of single cells have shown that Zn^2+^ is transiently stored in the Golgi^53,54^, where the metal is imported by ZnT5, ZnT6, and ZnT7. Notably, ZnTs are proposed to act as Zn^2+^/H^+^ antiporters^10^. Thus, Zn^2+^ uptake into the Golgi is most likely coupled with H^+^ efflux into the cytosol. Accordingly, Zn^2+^ import activity is enhanced by Golgi acidification^55^, highlighting the crosstalk between pH and Zn^2+^ in the ESP. Conversely, the neutral pH^32^ and the paucity of ZnTs^11^ in the ER likely induce ERp44 to adopt the closed conformation, allowing client release. Previous studies reported that free Zn^2+^ concentration in the ER is kept within the range of ~1 pM or 1–5 nM in mammalian cells^56–59^. Such low Zn^2+^ concentrations and the neutral pH may synergize in facilitating client dissociation possibly with the help of unidentified reductases or other factors. Altogether, we propose that the pH- and the Zn^2+^-gradients in the ESP collaboratively modulate the structure and function of ERp44, enabling cycles of client binding, retrieval and release.

In this context, it is noteworthy that the expression of Zn^2+^ transporters (ZnT6, ZnT7, ZIP7, ZIP9, and ZIP13) in the Golgi is upregulated in conditions of Zn^2+^ deficiency or ER stress^60^. ER stress is also known to induce the expression of ZnT5^61^ as well as of ERp44 and ERGIC-53^50,62^. The co-regulation of ERp44 and ZnTs/ZIPs under stress conditions supports our notion that ERp44 is a Zn^2+^-dependent ESP chaperone.

Our findings suggest that the Zn^2+^-dependent subcellular localization of ERp44 is key to efficient client retention. Unlike PDI and other ER chaperones with KDEL-like motifs^63,64^, endogenous ERp44 localizes in ERGIC and *cis*-Golgi at steady state and is readily secreted upon RDEL deletion^27,28^. Thus, ERp44 rapidly cycles throughout the ESP. Mutations at site 1 or at site 2 lead to its predominant accumulation in the Golgi or the ER, respectively. Importantly, both mutations compromised ERp44 activity. We surmise that the rapid arrival of ERp44 in the ERGIC and *cis*-Golgi is beneficial to control the integrity of the secretome by surveying and capturing assembly intermediates or ER residents that gain access to those compartments. Rapid cycling—possibly involving ERGIC-53^28^—may enable sufficient ERp44 to accumulate in post-ER compartments in a conformation ready to bind clients and retrieve them to the ER^32^.

In conclusion, the metal-dependent allosteric activation of ERp44 reveals a regulatory role of Zn^2+^ for protein quality control in the ESP, besides its well-known functions in the metalloenzymes biogenesis^11^ and in transcriptional regulation^5,65^. Metal response element-binding transcription factor-1 senses cellular free Zn^2+^ through six zinc-finger motifs, controlling the expression of Zn^2+^ exporters and Zn^2+^ storage proteins, metallothioneins^66^. Transient Zn^2+^ binding is also known to inhibit several enzymes and transporters^67–69^. Thus, Zn^2+^ signaling is involved in a variety of regulatory pathways. Our work sheds light on yet another aspect of Zn^2+^ biology, with potential implications in surveilling the metallo-secretome and maintaining Zn^2+^ homeostasis.

## Methods

### Reagents and antibodies

4′,6-diamidino-2-phenylindol (DAPI; Polysciences, Warrington, PA), *N*-ethylmaleimide (NEM; Nacalai Tesque), *N*,*N*,*N*′,*N*′-Tetrakis(2-pyridylmethyl)ethylenediamine (TPEN; Dojindo Laboratories), Tetraethylenepentamine (TEPA; Sigma), and Zinc pyrithione (ZPT; Tokyo Chemical Industry) were commercially purchased. Antibodies were purchased as follows: mouse monoclonal antibodies against c-Myc (9E10; Santa Cruz) (1:10,000 for western blot (WB)), green fluorescent protein (GFP; 7.1 and 13.1; Roche) (1:1000 for WB), FLAG (M2; Sigma) (1:10,000 for WB), HA (12CA5; purified from 12CA5 hybridoma supernatant (ATCC)) (1:1000 for WB) and PDI (1D3; Enzo Life Sciences) (1:1000 for both WB and immunofluorescence (IF)), rabbit monoclonal antibodies against ERp44 (D17A6; Cell Signaling Technology) (1:1000 for WB), Rab7 (D95F2; Cell Signaling Technology) (1:200 for IF), and rabbit polyclonal antibodies against Calnexin (ADI-SPA-860; Enzo Life Sciences) (1:200 for IF), EEA1 (PM062; MBL) (1:200 for IF), ERGIC-53 (E1031; Sigma) (1:2000 for IF), GFP (A-6455; Molecular Probes) (1:5000 for WB), Halo (G9281; Promega) (1:1000 for WB) and GM130 (PM061; MBL; and a kind gift of Dr. A. De Matteis, Naples) (1:1000 for IF). The anti-Ero1α 2G4 monoclonal antibody was raised using the chimera GST-Ero1α as an antigen (ARETA International S.r.l.), as described previously^70^, and its specificity was validated by several assays, including ELISA, IF and immunoprecipitation. 2G4 antibodies were used at a 1:1000 dilution for WB. Polyclonal antibodies against human PDI (A66) were described previously^71^ and used at 1:500 for WB. Monoclonal anti-ERp44 antibodies (clones 36C9 and 2D5) (36C9; 1:1000 for WB, 2D5; 1:50 for IF) were previously described^28,50^. Zn^2+^ solution used for the experiments was prepared by diluting a stock solution of 2 M ZnCl_2_ in ~20 mM HCl with the indicated buffer. The stock solution and titrant solution were prepared just before measurements.

### Purification and characterization of ERp44 and Ero1α

A series of His to Ala mutations were introduced into bacterial expression vectors using a quick-change mutagenesis kit (Agilent) or a PCR-based method with appropriate sets of primers (Takara) (Supplementary Table 4). Designated mutations were verified by sequencing. Overexpression and purification of recombinant WT or mutant ERp44 variants (Supplementary Table 4) were performed as described previously^32^. Ero1α Cys104/Cys131Cys166Ala mutant was prepared as described previously^39^ and used in all the experiments. The histidine tag of all recombinant samples was cleaved by thrombin treatment.

ANS fluorescence spectra were recorded in 1 cm cuvettes on a Hitachi F-7000 spectrofluorometer as described previously^37^. Samples were prepared as follows: (a) recombinant ERp44 WT (5 µM) in buffer (20 mM BisTris–HCl pH 7.2 or 6.2, 150 mM NaCl); (b) ERp44 (5 µM) was mixed with 10 µM ZnCl_2_ and incubated at 293 K for 5 min; (c) subsequent addition of EDTA (1 mM) into sample B and further incubated at 293 K for 5 min. These samples were mixed with 40 µM ANS, and incubated at 293 K for 10 min before measurement.

The far-UV CD spectra were recorded with ERp44 WT and its mutants (5.7 µM) in 10 mM Bis–Tris pH 7.0 on a J-720 CD spectropolarimeter (JASCO) at room temperature using a cuvette of 1 mm path-length.

### Isothermal titration calorimetry analysis

Isothermal titration calorimetry (ITC) experiments were carried out using the MicroCal™ iTC200 calorimeter (GE Healthcare). Concentrated samples were diluted into 20 mM BisTris–HCl pH 6.2, 6.7, or 7.2 and 150 mM NaCl at 283 K. Typically, after a 0.4 µL initial injection, 0.8 or 1 µL of titrant solution was injected into protein solution at 150 s intervals with stirring 750 rpm. For Zn^2+^ binding analysis, 48 injections of 0.8 µL of 500 µM ZnCl_2_ were added into 30 µM ERp44. For the interaction between ERp44 and Ero1α at pH 6.2, 24 injections of 1.5 µL of 420 µM Ero1α were added into 30 µM ERp44 WT. For the interaction between ERp44 and Ero1α in the presence of Zn^2+^ at pH 6.2, 24 injections of 1.5 µL of 200 µM ERp44 were added into 15 µM Ero1α and 15 µM ZnCl_2._ For dilution titration, 20 injections of 2 µL of 10 or 100 µM ERp44 with 20 or 200 µM ZnCl_2_ in 20 mM BisTris pH 6.2, 150 mM NaCl were added into the same buffer. Each condition was repeated at least twice.

The integration of the observed injection peaks was performed with NITPIC^72^ and the global analysis was performed with SEDPHAT^73^. We thereby obtained stoichiometry (*n*), apparent binding affinity (*K*_a_) and enthalpy change (Δ*H*) for Zn^2+^ binding to ERp44 using a 1:1 binding model, as described in the previous literature^74^.

Scatchard plots of the observed ITC data for Zn^2+^-binding to ERp44 were calculated as described previously^48^. Briefly, the fraction of bound Zn^2+^ vs. total ERp44 (*R*(*i*) = *X*_bound_(*i*)/*M*_t_(*i*)) was plotted against *R*(*i*)/*X*_free_(*i*), where *X*_bound_(*i*) is the concentration of Zn^2+^ bound to ERp44 after the *i-*th injection, *M*_t_(*i*) is the total concentration of ERp44 after the *i-*th injection, which was automatically corrected by the Origin program considering the volume increase caused by every injection, and *X*_free_(*i*) is the concentration of free Zn^2+^ after the *i*-th injection. The *X*_bound_(*i*) value was calculated using the following equation:

1 $$X_{{\mathrm{bound}}}\left( i \right) = [Q\left( i \right)/((\Delta H + q_{{\mathrm{dilute}}})V_0)] + X_{{\mathrm{bound}}}\left( {i - 1} \right)$$

where *Q*(*i*) is the heat generated on the *i*-th injection measured by the ITC experiment, Δ*H* is the enthalpy change, *q*_dilute_ is estimated normalized heat of dilution, and *V*_0_ is the active cell volume. The *X*_free_(*i*) value was calculated using the following equation:

2 $$X_{{\mathrm{free}}}\left( i \right) = X_{\mathrm{t}}\left( i \right)-X_{{\mathrm{bound}}}\left( i \right)$$

where *X*_t_(*i*) is the total concentration of Zn^2+^ after the *i*-th injection.

In the saturation plots based on the observed ITC, *X*_free_(*i*) was plotted against the fractional saturation (*R*(*i*) = *X*_bound_(*i*) / *M*_t_(*i*)). The hill coefficients were estimated with Prism software version 7.0a (GraphPad Software).

### SEC and SEC-MALS analysis

SEC was performed with a superdex 200 increase 10/300 column (GE healthcare) using an ÄKTA explorer 10S system. 50 µL of samples containing 60 µM ERp44 WT or mutants and 120 µM ZnCl_2_ ions was incubated on ice for >30 min and then loaded onto the column equilibrated in 20 mM BisTris–HCl pH 7.0 (Fig. 1c), 6.2 (Fig. 4d, Supplementary Fig. 1B), 6.7 (Supplementary Figs. 1B and 8C), or 7.2 (Supplementary Figs. 1B) and 150 mM NaCl at 277 K. Fractions were treated with 20 mM NEM for >10 min and thereafter analyzed on 10% SDS-PAGE gels. For the complex between ERp44 and Ero1α in the presence of Zn^2+^, a mixture of 40 µM Ero1α and 80 µM ZnCl_2_ was mixed with equimolar ERp44 and was incubated on ice for >5 min and then loaded onto the column equilibrated in 20 mM BisTris–HCl pH 6.2 and 150 mM NaCl at 277 K. For the interaction between the Zn^2+^-bound ERp44 with Ero1α, a mixture of 40 µM ERp44 and 80 µM ZnCl_2_ was mixed with 40 µM Ero1α and was incubated on ice for >5 min and then loaded onto the column.

SEC-multi-angle light scattering (MALS) experiments were carried out with a superdex 200 10/300 column equilibrated in 20 mM BisTris pH 7.0 and 150 mM NaCl using an ÄKTA FPLC system. 50 µL of samples containing 60 µM ERp44 WT and three fold excess Zn ions was incubated on ice for >30 min and then loaded onto the column at room temperature. For the Zn^2+^-bound ERp44–Ero1α interaction, a mixture of 60 µM ERp44 and 120 µM ZnCl_2_ was mixed with 60 µM Ero1α and was incubated on ice for >30 min and then loaded onto the column. The column was connected to a miniDAWN TREOS detector, followed by a refractive-index detector RI-101 (Shodex). Molecular masses were calculated with ASTRA V (Wyatt Technology) with the d*n*/d*c* value of 0.185 mL/g.

### Crystallization

Crystals of the ERp44–Zn^2+^ complex grew for several days in the sitting drop vapor diffusion method at 277 K, by mixing 0.7 µL of the protein solution (10 mg/mL protein, 0.5–2 mM ZnCl_2_, 20 mM HEPES-NaOH pH 7.0, and 150 mM NaCl) with 0.7 µL of the precipitant solution (1.0 M Na malonate, 0.09 M HEPES-NaOH pH 7.0, 0.45% v/v Jaffamine^®^ED-2001 pH 7.0) or 39% Tascimate. Before data collection, a cryoprotectant solution (2.2 M Na malonate pH 7.0, 0.1 M HEPES-NaOH pH 7.0, 0.5–2 mM ZnCl_2_) was added into drops several times and then crystals were flash-cooled in a nitrogen stream at 100 K.

### Data collection and structure determination

The X-ray diffraction data were collected at a wavelength of 0.9 Å on beamline BL44XU at SPring-8 (Proposal No., 2014A/B6904, 2015A/B6558, 2016A/B6656) and at a wavelength of 1.1 Å on beamline BL1A at the Photon factory. The diffraction images were processed with XDS and XSCALE^75^. The structure of the ERp44–Zn^2+^ complex was determined at 2.45 Å resolution by the SAD method using anomalous signals of Zn^2+^ (Supplementary Table 5). We first sought to determine the structure of Zn^2+^-bound ERp44 by molecular replacement using previously published crystal structures of Zn-free ERp44 (PDB ID: 2R2J, 5GU6, and 5GU7) and a variety of their fragments containing one or two thioredoxin (Trx)-like domains as search models. However, we anyhow failed to reach the correct answer. We therefore decided to employ the SAD method using anomalous signals of bound Zn^2+^ for phase determination. As we could not collect diffraction datasets with high redundancy from a single crystal due to radiation damage, we merged and scaled anomalously multiple isomorphous datasets with a total rotation range of ~80˚ for each (Supplementary Table 6). Merging seven datasets with high CC values allowed the SAD phasing and improved resolution and statistics compared to each native dataset. Consequently, clear electron density maps were provided for the entire regions including the C-tail. Substructure determination, phasing and density modification were performed with SHELXD and SHELXE^76^, followed by auto-model building with Buccaneer^77^. Further manual model building was performed with COOT^78^, refined with PHENIX^79^ and validated with MolProbity^80^. A Fo–Fc map showed two additional positive peaks nearby Zn3, to which two Cl^−^ ions were assignable, since placing Cl^−^ ions at these positions, but not water molecules, resulted in no extra electron density in the Fo–Fc map and gave reasonable B-factors for these ions (Supplementary Table 7). Structural Figures were prepared with PyMOL (http://www.pymol.org) and Chimera^81^.

### Cell culture and transfection

HepG2 cells (purchased from ATCC) were grown in Dulbecco’s modified Eagle’s medium (DMEM; Nacalai Tesque) containing 10% fetal calf serum (FCS). Different HeLa sublines were utilized and kept at 5 or 10% FCS in DMEM. Small interfering RNAs (siRNAs) duplexes were transfected using Lipofectamine RNAiMAX reagent (Life Technologies). Plasmids were transfected using FuGENE-HD transfection reagent (Promega) (Figs. 1g, 5a, c and Supplementary Fig. 3B, 4C, 4H, 12A, 12C and 14) or as described previously in Vavassori et al.^32^ (Fig. 2c and Supplementary Fig. 13).

### Immunofluorescence

To analyze the localization of endogenous ERp44 in HeLa cells, cells plated onto poly-l-lysine coated coverslips were incubated in Opti-MEM containing 10 µM TPEN for 30 min and some of them were further incubated in Opti-MEM containing 20 µM ZnSO_4_ for 3 h. Since chloride ions are known to precipitate Fe^3+^, an essential metal ion, at the neutral pH, we used ZnSO_4_ instead of ZnCl_2_ to supplement Zn^2+^ to cultured cells. Cells were fixed with PBS containing 4% PFA, permeabilized with PBS containing 0.1% TritonX-100 and blocked with PBS containing 2% FCS for 1 h at room temperature. Cells were then incubated with antibodies against ERp44 (2G5) and GM130 for 90 min. CF488-conjugated anti-mouse IgG and CF568-conjugated anti-rabbit IgG antibodies (Biotium) were used as secondary antibodies. Nuclei were stained with DAPI. Fluorescent images were obtained using a confocal microscope (FV1000, Olympus) equipped with a UPLSAPO 60x silicon oil-immersion objective lens (NA 1.30). Pearson’s correlation coefficient values were calculated using ImageJ software.

For HepG2 cells, cells plated onto 13 mm coverslips were treated as described above. At the end of treatments, cells were fixed with PBS containing 4% paraformaldehyde, washed and incubated with 0.1% glycine in PBS for 15 min, permeabilized with PBS containing 0.1% TritonX-100, and blocked in PBS containing 5% FCS and 5% BSA for 30 min at RT. Cells were then incubated with or without monoclonal antibodies against ERp44 (2D5) for 1 h, then with rabbit anti-GM130 antibodies for 30 min at RT. After extensive washing, slides were incubated with Alexa Fluor 546 goat anti-rabbit and Alexa Fluor 647 goat anti-mouse (Thermo Fischer Scientific) secondary antibodies. Nuclei were stained with Hoechst (1:1000). Fluorescent images were obtained using a confocal laser scanning microscopy (Leica TCS SP8 SMD FLIM) equipped with an oil lens (HC PL APO CS 2 ×63 (NA 1.4)) and deconvoluted with Huygens Essential. Pearson’s correlation coefficient values were calculated using the Volocity software (PerkinElmer). The experiments with ZnT5/6/7 triple knockdown were processed similarly.

To analyze the intracellular localization of YFP-ERp44 and its mutants, HeLa cells plated on poly-l-lysine coated coverslips were transfected with plasmids and cultured for 36 h. Cells were washed with 1× HHBSS (5.36 mM KCl, 137 mM NaCl, 16.65 mM d-Glucose, and 30 mM HEPES, pH 7.4)^82^ three times, preincubated in Opti-MEM for 30 min, and then stimulated with TPEN (10 µM) for 30 min and subsequent ZPT (final 20 µM) for 15 min. DMSO was used as a vehicle control for each steps. Cells were then fixed and immunostained for GM130 and PDI as described above. CF633-labeled anti-mouse IgG, and CF568-labeled anti-rabbit IgG antibodies (Biotium) were used as the secondary antibodies. Nuclei were stained with DAPI. Fluorescent images were obtained using a confocal microscope (FV1000, Olympus) equipped with a UPLSAPO ×60 oil-immersion objective lens (NA 1.35) or UPLSAPO ×60 silicon oil-immersion objective lens (NA 1.30). Pearson’s correlation coefficient values were calculated using ImageJ software.

### Time-lapse analysis

HeLa cells (1.0 × 10^5^ cells) were plated on poly-l-lysine coated glass bottomed dish and transfected with plasmids. After incubation for 36 h, cells were further cultured in Opti-MEM for 1 h and then subjected to time-lapse analysis using a confocal microscope (FV1000, Olympus) equipped with a UPLSAPO ×60 oil-immersion objective lens (NA 1.35). Cells were treated with TPEN (final 10 µM) at *T* = 2 min and subsequently ZPT (final 20 µM) at *T* = 32 min. We used ZPT to increase intracellular Zn^2+^ concentration in a short time frame in the live-cell imaging analysis.

### ZnT knockdown

The Silencer Select siRNAs targeting human ZnT5, ZnT6, and ZnT7 were purchased from ThermoFisher Scientific (Supplementary Table 1). The relative amount of mRNA remaining 48 h after transfection was determined by qRT-PCR analysis using THUNDERBIRD SYBR qPCR Mix (Toyobo) or Sybr Green RT PCR Master Mix (ThermoFisher). The siRNA sequences and the primers used for qRT-PCR are listed in Supplementary Table 2 and Supplementary Table 3. To observe the effects of ZnT knockdown on the localization of ERp44, HeLa cells were transfected with 5 nM each siRNA mixture for 24 h and transfected with YFP-ERp44. After additional incubation for 24 h, cells were fixed and counterstained for GM130.

### Secretion assay for ERp44 and its clients

To monitor client retention by ERp44 variants, HeLa cells (3.0 × 10^5^ cells) were plated on 60-mm dishes and transfected with 1 µg of Ero1α-Myc and 100–200 ng of YFP-Mock or YFP-ERp44 expressing plasmids. After incubation for 24 h, cells were washed with 37 °C pre-warmed Opti-MEM twice and incubated in Opti-MEM for 6 h. To concentrate the secreted proteins, conditioned media were collected and clarified by centrifugation. Then, aliquots of supernatants were precipitated with ice-cold 10% TCA. Precipitants were washed with acetone and dissolved in 1× SDS-loading buffer. To analyze intracellular proteins, cells remaining on plates were lysed in 1× SDS-loading buffer and homogenized by sonication. Samples were denatured at 97 °C for 5 min and subjected to SDS-PAGE. Protein bands were visualized by immunoblotting analyses. The signal intensity of relevant bands was measured by ImageJ software (NIH) or Image Lab software (Bio-Rad).

To analyze the effect of specific and targeted Zn^2+^ deprivation, HeLa cells (3.0 × 10^5^ cells) were silenced using siRNAs targeting human ZnT5, ZnT6, and ZnT7 (20 µM each). After 24 h, cells were then transfected with plasmids driving the expression of Ero1α-Myc or ERAP1-FLAG (2 µg). After further 48 h, cells were washed twice with Opti-MEM preheated at 37 °C and incubated in the same medium for 4 h. The spent media were analyzed as above. Cells were detached with trypsin, washed and lysed in 1% NP-40, 0.1% SDS, 150 mM NaCl, 10 mM Tris–HCl, pH 7.4, 20 mM NEM and protease inhibitors. Nuclei and cell debris were discarded by centrifugation. Aliquots of the cell lysates and supernatants were denatured at 97 °C for 5 min, resolved by 4–12% gradient precast gels (Invitrogen) under reduced conditions and analyzed by immunoblotting.

### Sequential elution assay

HepG2 cells (1.5 × 10^6^ cells) were plated on 10 cm dishes and transfected with plasmids driving the expression of Halo-RDEL, Halo-ERp44 WT or Halo-ERp44 3HA, using FuGENE-HD. In some experiments cells were co-transfected also with HA-tagged ERp44 WT or 3HA to better detect ‘homo’-oligomerization. After 48 h, cells were lysed in 1% NP-40, 150 mM NaCl, 10 mM Tris–HCl, pH 7.4 for 20 min on ice and detached by gentle scraping of the culture dishes. Post-nuclear supernatants (PNS) were collected and 1 mg of total protein incubated overnight with immobilized Halo-ligands (Halo-Tagged protein purification resins, Promega). Beads were then washed twice with 150 mM NaCl, 10 mM Tris–HCl, pH 7.5, 0.0005% NP-40, and then sequentially eluted with TPEN (100 µM) and, after further washes in the above buffer, eluted with dithiothreitol (DTT, 200 mM) and 1% SDS. In case of Zn^2+^-supplemented conditions, ZnCl_2_ (50 µM) was added to the lysis and wash buffers. Samples were concentrated by TCA precipitation as described above, resuspended in loading buffer, boiled for 5 min and resolved by 4–12% gradient precast gels under reducing conditions. Proteins of interest were visualized by immunoblotting using antibodies specific for ERp44 (36C9 monoclonal), Halo or HA, as described in figure legends.

### GST-GFP-nanobody pull-down assay

GST-GFP-Nanobody proteins were bacterially expressed using BL21 strain and conjugated to glutathione-Sepharose beads. HeLa cells (3.0 × 10^5^ cells) plated on 60-mm plate were transfected with plasmids and cultured for 36 h. Cells were washed with 1× HHBSS three times and culturing media were exchanged to Opti-MEM containing 2.5 µM ZPT or DMSO and incubated for 15 min. Cells were then lysed in lysis buffer (50 mM HEPES-NaOH, pH 7.0, 150 mM NaCl, 10% glycerol, 1% NP-40, 0.1% SDS, 20 mM NEM and Protease inhibitor cocktail (Nacalai Tesque)). Lysates were homogenized by sonication and clarified by centrifugation and resultant supernatant were precleared by incubation with Protein G Sepharose beads at 4 °C for 1 h with rotation. After incubation, beads were precipitated by centrifugation and supernatants were incubated with GST-GFP-Nanobody-conjugated beads at 4 °C for 3.5 h with rotation. Then, beads were washed with lysis buffer three times and dissolved by 1× SDS-loading buffer. Precipitated proteins were denatured at 70 °C for 10 min and subjected to SDS-PAGE and immunoblotting analyses.

### Gel filtration analysis for microsomal lysates

HEK293T cells from four 15 cm dishes were cultured to confluence, harvested and homogenized in a buffer containing 10 mM HEPES-NaOH, pH 7.5, 250 mM sucrose, 1 mM EGTA, 0.1 mg/mL DNaseI, and protease inhibitor cocktail (Nacalai Tesque) by Dounce-type homogenizer until cells were sufficiently disrupted. Unbroken cells and nuclei were removed by three times of centrifugation at 2000×*g* for 10 min. The resultant supernatants (i.e., PNS) were subjected to ultracentrifugation (100,000 × *g*, 1 h) to precipitate microsomes. Microsomes were then resuspended in lysis buffer B (20 mM BisTris–HCl, pH 7.2, 150 mM NaCl and protease inhibitor cocktail) by passing through 25 G needle and lysed by brief sonication on ice. Lysates were clarified by sequential centrifugation at 21,000 × *g* (30 min) and 100,000 × *g* (30 min) and filtration through 0.22 µm filter. Clarified lysate was loaded onto Superdex 200 Increase 10/300 column equilibrated in 20 mM BisTris–HCl, pH 7.2 and 150 mM NaCl. The fractions were mixed with NEM (final 1 mM) and 5× Laemmli sample buffer and analyzed by immunoblotting. To test the effect of Zn^2+^ and disulfide bonds, TPEN (final 100 µM) and TCEP (final 1 mM) was added into PNS and lysis buffer B, respectively.

### Statistical analyses

Statistical analyses were performed with Prism software version 7.0a (GraphPad Software), using a two-tailed unpaired *t*-test or one-way ANOVA followed by Dunnett’s test or Tukey’s test for comparison of multiple datasets. Data represent the means of the indicated number of independent experiments. Error bars indicate the standard error of the mean (SEM) or the standard deviation (SD). *p* < 0.05 was considered to be significant.

## Supplementary information

Supplementary Information

Peer Review File

Description of Additional Supplementary Files

Supplementary Movie 1

Supplementary Movie 2

Supplementary Movie 3

Source Data

Reporting Summary

## Data Availability

Coordinates and structure factors have been deposited in the Protein Data Bank with accession code 5XWM. The source data underlying Figs. 1c, 2b, 4c, and 5c and Supplementary Figs. 12A–C, 13B, C, E are provided as a Source Data file. Other data are available from the corresponding authors upon reasonable request.
