# Supplementary material for: Zinc regulates ERp44-dependent protein quality control in the early secretory pathway
Source: Nat Commun. 2019 Feb 5;10:603. doi: 10.1038/s41467-019-08429-1 (PMC6363758; doi:10.1038/s41467-019-08429-1)
Supplement: Supplementary file 1 — Supplementary Information [file 41467_2019_8429_MOESM1_ESM.pdf]

Zinc regulates ERp44-dependent protein quality control in the early secretory pathway

S. Watanabe, Y. Amagai, S. Sannino et al.

Supplementary information

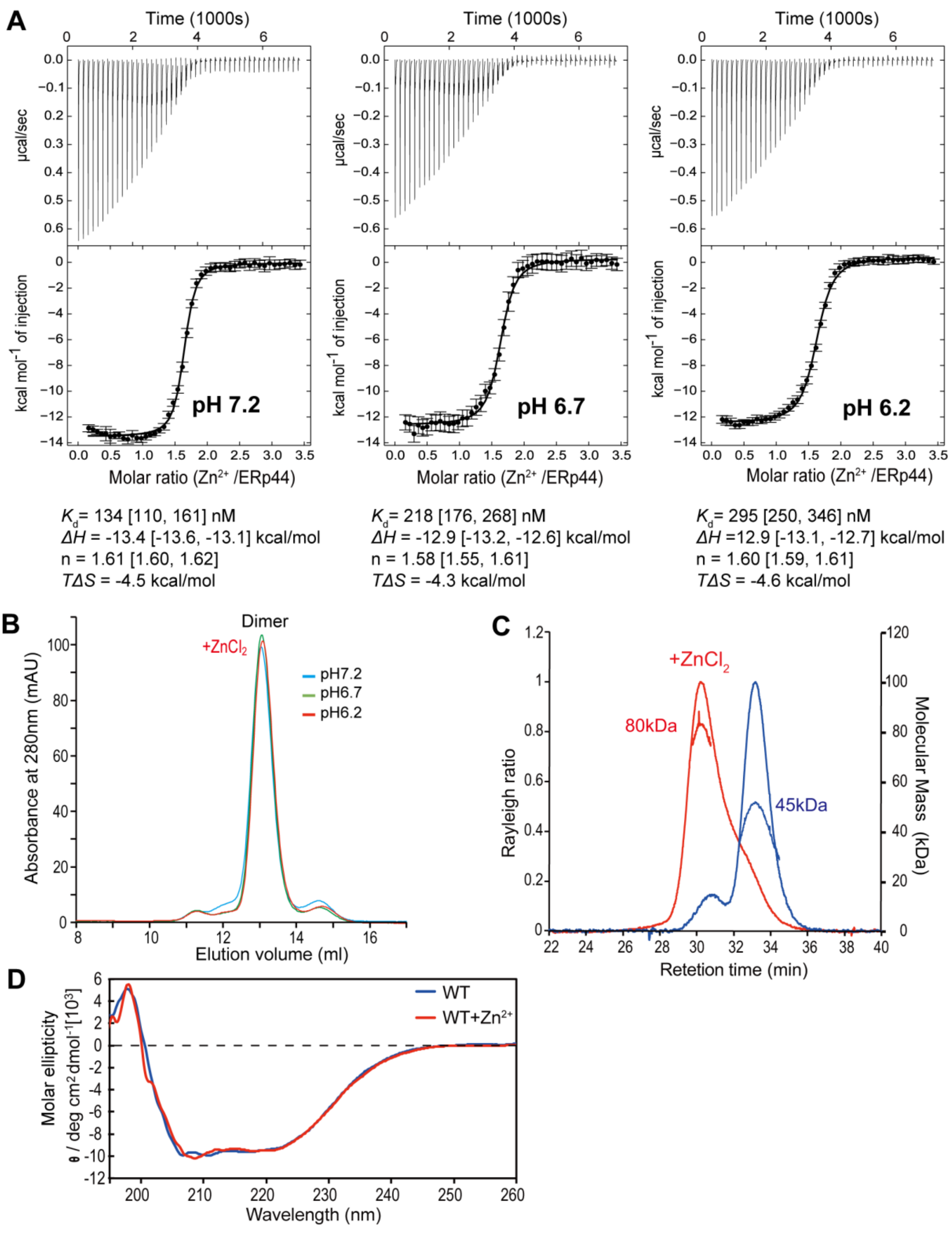

Supplementary Figure 1. Biophysical characterization of the ERp44-Zn^{2+} interaction,

(A) ITC raw data (upper) and binding isotherm data (lower) for titration of $ZnCl_2$ (500 µM) into ERp44 (30 µM) at pH 7.2 (left), 6.7 (middle) and 6.2 (right). Bars represent the errors in the peak integration for each injection estimated by NITPIC. The global analysis was performed to estimate the K_d and ΔH values with SEDPHAT using a 1:1 binding model. The calculated thermodynamic parameters are shown with 68.3 % confidence interval in brackets.

(B) SEC analysis of ERp44 (60µM) in the presence of $ZnCl_2$ (120µM) at pH 7.2, 6,7 and 6.2, respectively.

(C) SEC-MALS analysis of ERp44 (60 µM) in the presence or absence of Zn^{2+}. Averaged molecular

masses calculated by MALS are indicated for the main peaks. Compared to the elution profile in the SEC analysis performed at 277K (Fig. 1C), the Zn^{2+}-bound homodimer was eluted as a broader peak in the SEC-MALS analysis performed at room temperature, suggesting that the Zn^{2+}-bound ERp44 dimer partially disassembles at room temperature. To stabilize the dimer, we added higher concentration (180 µM) of $ZnCl_2$ in the SEC-MALS experiment than in the normal SEC experiment. (D) Far-UV circular dichroism (CD) spectra of ERp44 WT (5.7µM) in the presence (12 µM) or absence of Zn^{2+}.

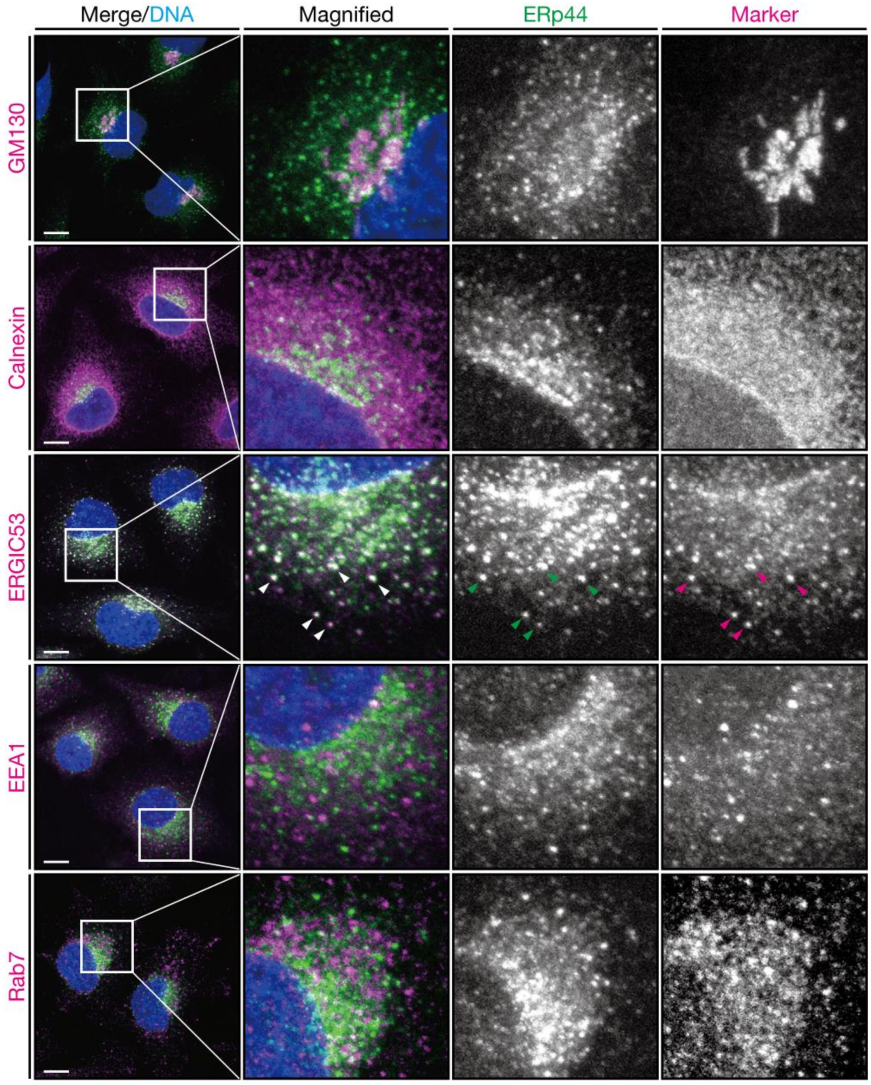

Supplementary Figure 2. Vesicle-like structure of ERp44 nicely colocalizes with ERGIC53

HeLa cells were immunostained for endogenous ERp44 and several organelle markers, such as GM130 (*cis*-Golgi), Calnexin (ER), ERGIC53 (ERGIC), EEA1 (early endosome), and Rab7 (late endosome). Arrowheads indicate the colocalization of the vesicles of ERp44 with the ERGIC53. Scale bars, 10 µm.

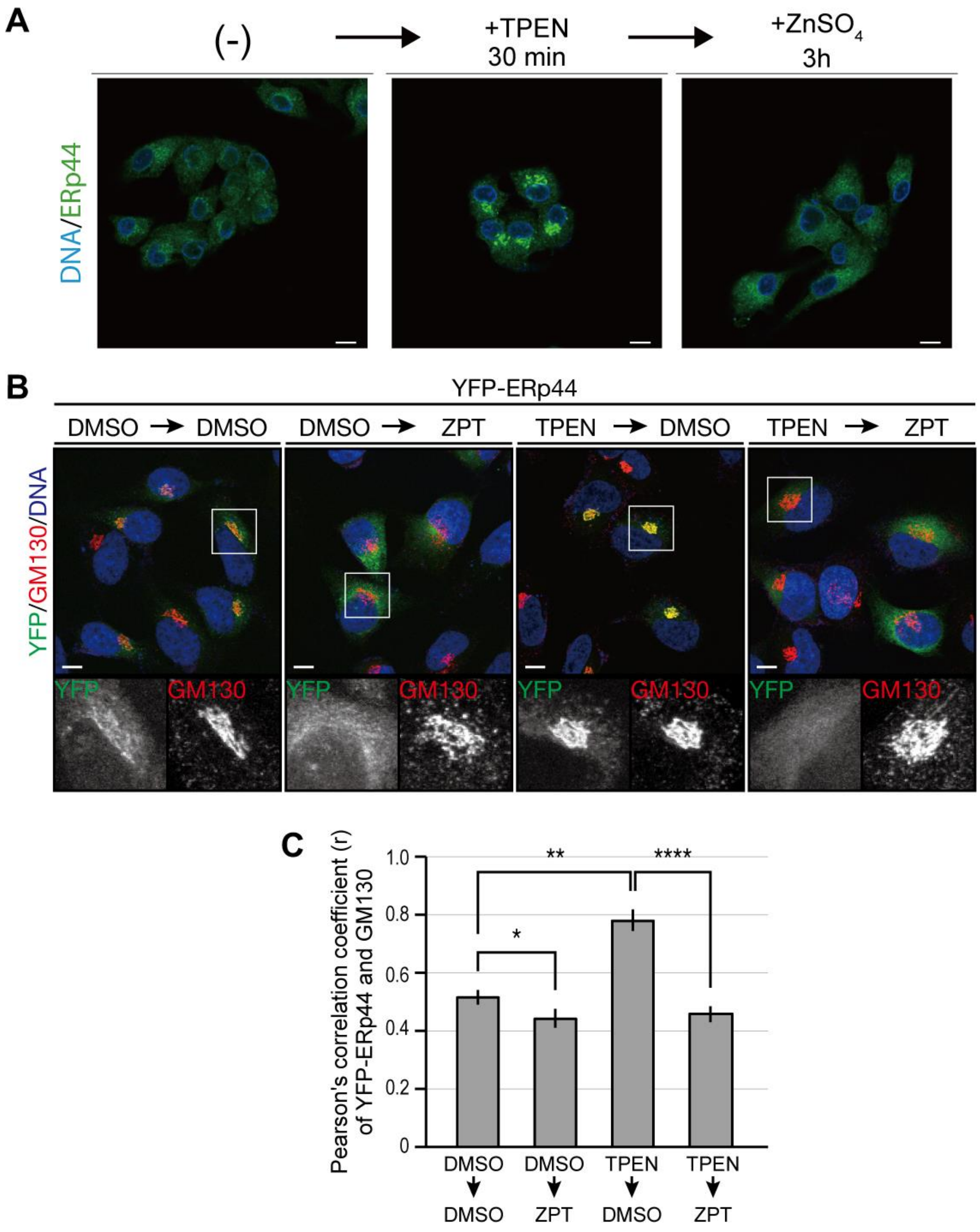

Supplementary Figure 3. Zn^{2+}-dependent subcellular localization of ERp44

(A) Zn^{2+}-dependent subcellular localization of endogenous ERp44 in HepG2 cells. Confocal immunofluorescence images show movement of endogenous ERp44 to a more perinuclear area (likely corresponding to the Golgi compartment) after 30 minutes of exposure to 10 μM TPEN. Following incubation with excess $ZnCl_2$ (20 μM) for 3 hours causes its re-localization in the ER. Scale bar, 10 μm.

(B) Zn^{2+}-dependent subcellular localization of YFP-tagged ERp44 in HeLa cells. HeLa cells were plated on coverslips, transfected with YFP-ERp44 (WT), and cultured for 36 h. Cells were treated with TPEN (10 μM) for 30 min and subsequently with ZPT (20 μM) for 15 min and then fixed. Fixed cells were stained for GM130. DMSO was used as a vehicle control in each condition. The right panels show quantitative analyses of Pearson's correlation coefficients for the co-localization of YFP-ERp44 with GM130. Data are the means ± SEM (N=3, > 30 cells per experiment, one-way ANOVA followed by Tukey's test). ****, $p < 0.0001$; **, $p < 0.01$; *, $p < 0.05$. Scale bar, 10 μm.

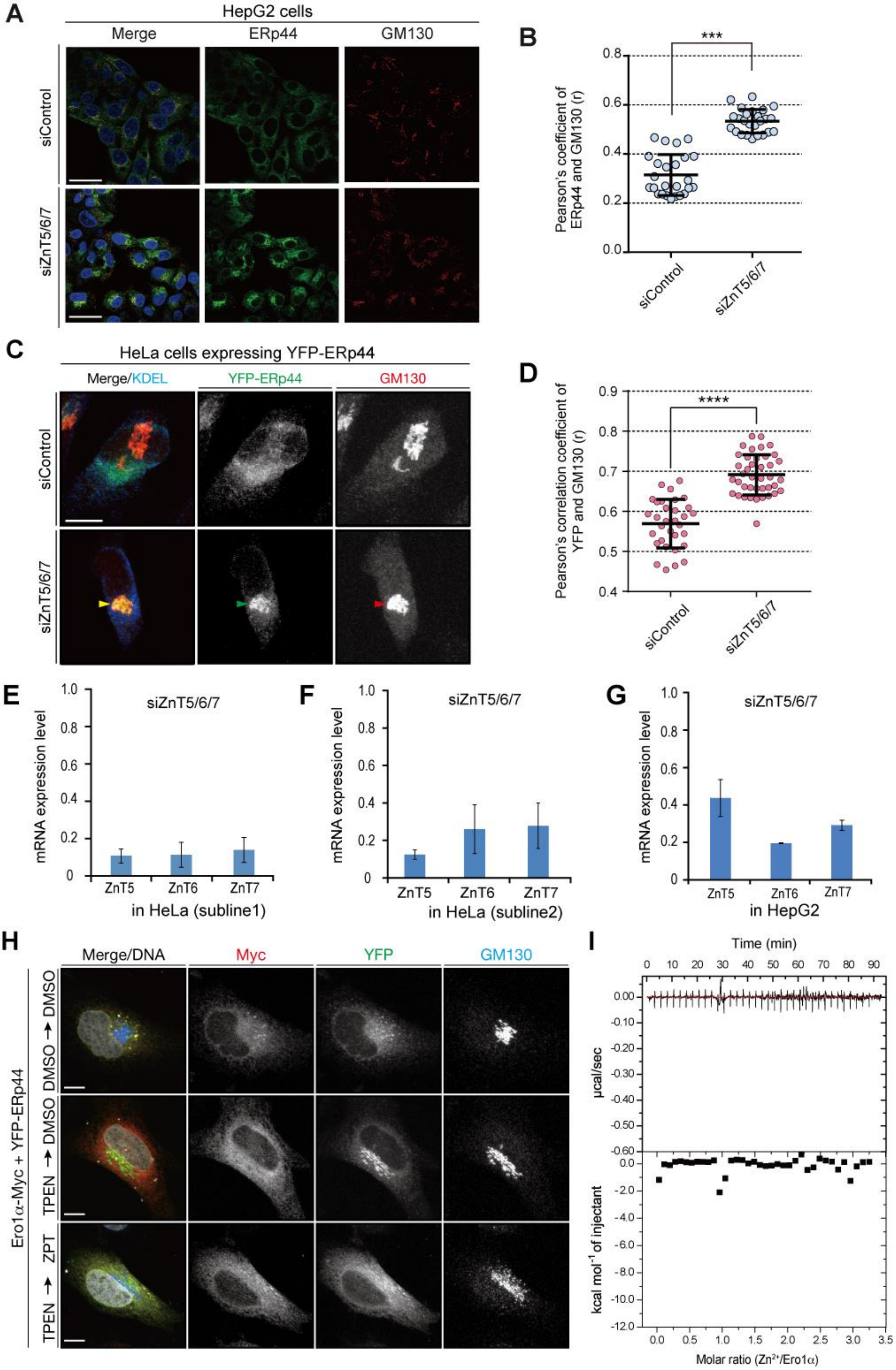
A
HepG2 cells
Merge
ERp44
GM130
siControl
siZnT5/6/7
B
Pearson's coefficient of ERp44 and GM130 (r)
***
0.8
0.6
0.4
0.2
0.0
siControl
siZnT5/6/7
C
HeLa cells expressing YFP-ERp44
Merge/KDEL
YFP-ERp44
GM130
siControl
siZnT5/6/7
D
Pearson's correlation coefficient of YFP and GM130 (r)
****
0.9
0.8
0.7
0.6
0.5
0.4
siControl
siZnT5/6/7
E
siZnT5/6/7
mRNA expression level
ZnT5
ZnT6
ZnT7
in HeLa (subline1)
F
siZnT5/6/7
mRNA expression level
ZnT5
ZnT6
ZnT7
in HeLa (subline2)
G
siZnT5/6/7
mRNA expression level
ZnT5
ZnT6
ZnT7
in HepG2
H
Merge/DNA
Myc
YFP
GM130
Ero1α-Myc + YFP-ERp44
DMSO → DMSO
TPEN → DMSO
TPEN → ZPT
I
Time (min)
μcal/sec
kcal mol⁻¹ of injectant
Molar ratio (Zn²⁺/Ero1α)

Supplementary Figure 4. ZnT5, 6 and 7 knockdown altered subcellular localization of ERp44, but not Ero1α

(A) Confocal immunofluorescence images showing the intercellular localization of endogenous ERp44 (in green) in HepG2 cells with ZnT5/6/7 triple knockdown, with respect to untreated cells (siControl). Cells were co-stained for GM130 (in red) and DAPI (in blue). Note that ERp44 accumulates in the Golgi upon ZnT5/6/7 triple knockdown. Scale bar, 10 µm.

(B) Quantitative analyses of Pearson's correlation coefficients for the co-localization of endogenous ERp44 with GM130 based on the immunofluorescence images shown in A. Dots indicate individual data points (> 30 cells for each conditions). Bars indicate the means ± SD. ***, $p < 0.0001$

(C) Subcellular localization of YFP-ERp44 in ZnT5/6/7 triple knockdown HeLa cells. Scale bar, 10 µm.

(D) Quantitative analyses of Pearson's correlation coefficients for the co-localization of YFP-ERp44 with GM130 based on the immunofluorescence images shown in C. Dots indicate individual data points (> 30 cells for each conditions). Bars indicate the means ± SD. ****, $p < 0.00001$

(E-G) RT-PCR analyses confirm the efficiency of ZnT5, ZnT6 and ZnT7 silencing in HeLa cells subline1 (E), subline2 (F) and HepG2 cells (G). Average of 3 independent experiments ± SD.

(H) Cells expressing Ero1α-Myc and YFP-ERp44 were treated with or without TPEN and ZPT and analyzed by immunofluorescence. Note that most Ero1α-Myc remains in the ER whilst YFP-ERp44 accumulates in the Golgi upon Zn^{2+} depletion. This is likely due to retention of Ero1α-Myc by PDI (Otsu et al., 2006) as well as rapid secretion of Ero1α-Myc molecules from the Golgi. Scale bar, 10 µm.

(I) ITC raw data (upper) and binding isotherm data (lower) for titration of $ZnCl_2$ (500 µM) into Ero1α (30 µM) at pH 7.0

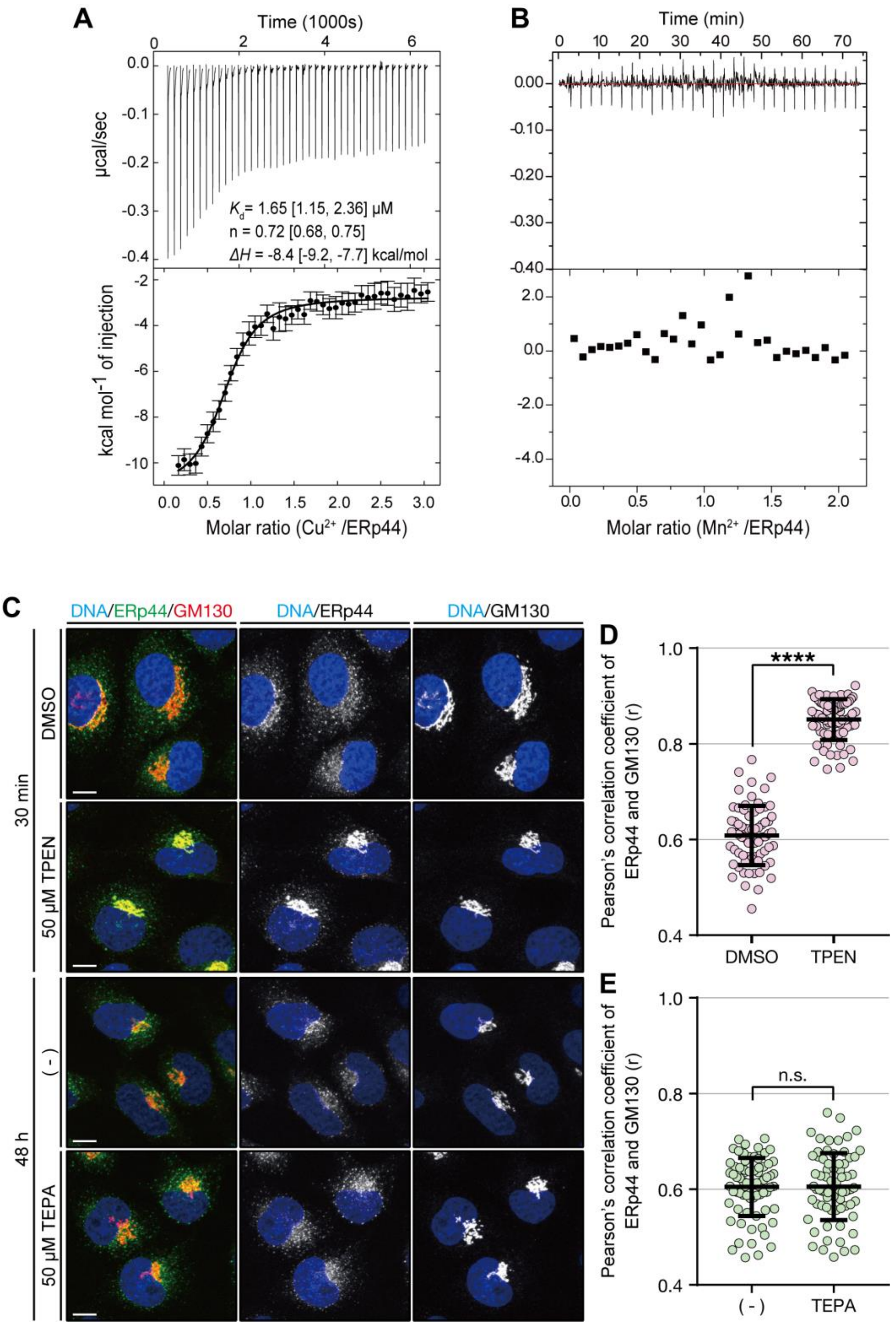

Supplementary Figure 5. Chelation of copper ions hardly affects the subcellular localization of ERp44

(A) ITC raw data (upper) and binding isotherm data (lower) for titration of $CuCl_2$ (500 µM) into ERp44 (30 µM) at pH 7.2

(B) ITC raw data (upper) and binding isotherm data (lower) for titration of $MnCl_2$ (500 µM) into ERp44 (30 µM) at pH 6.7

(C) Immunofluorscence analysis of cells treated with Zn^{2+}-chelator TPEN or Cu^{2+}-chelator TEPA. HeLa cells were treated with 50 µM TPEN or DMSO in complete medium for 30 min or with 50

µM TEPA in complete medium or not for 48 h. Cells were fixed with PFA and immunostained for ERp44 and GM130. Scale bars, 10 µm.
(D, E) Quantitative analyses of Pearson's correlation coefficients for the co-localization of ERp44 with GM130. Data are the mean ± SD (≥ 70 cells from two independent experiments). Unpaired t-test, ****, $p < 0.0001$, n.s., not significant ($p > 0.05$).

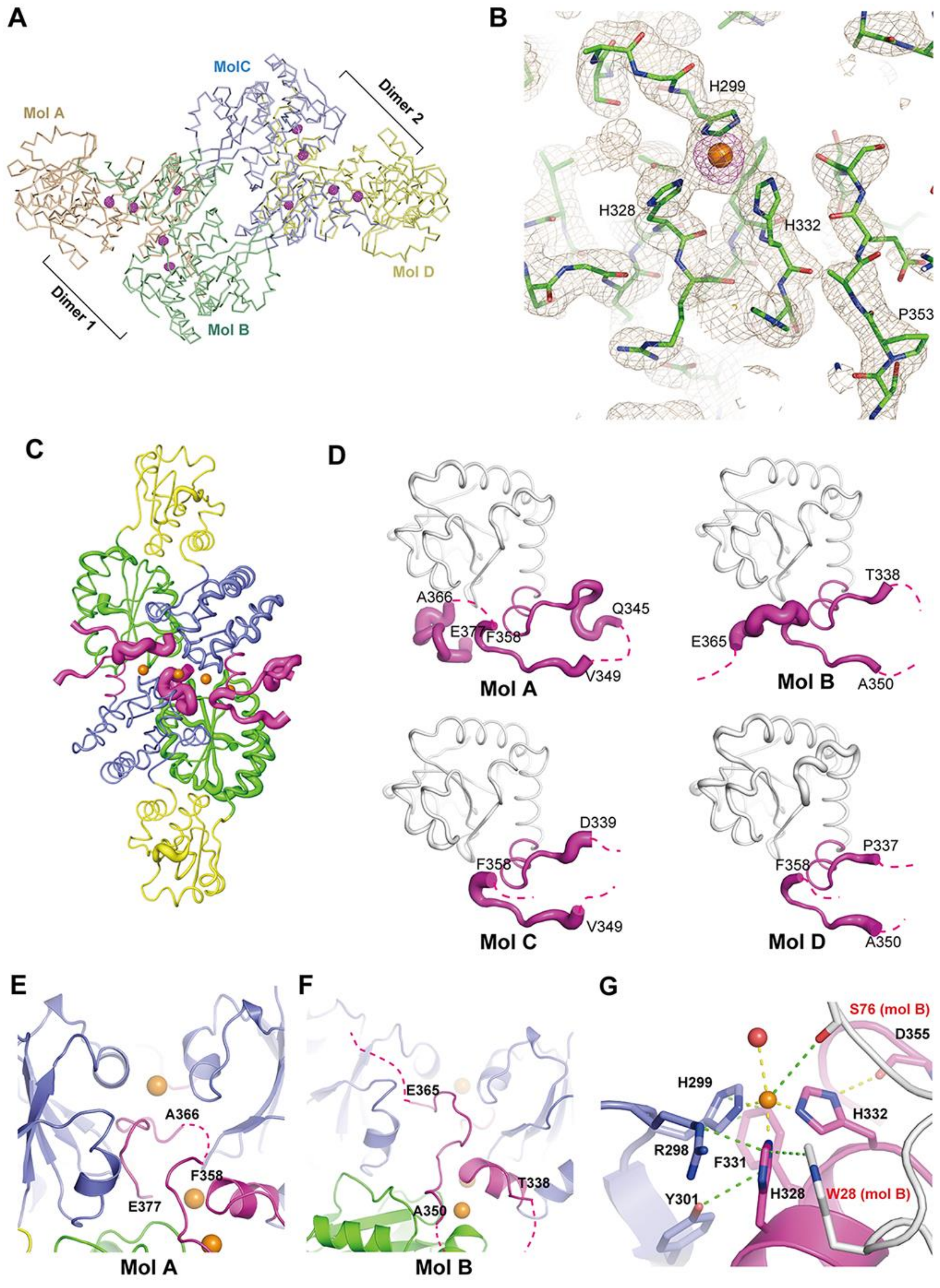

Supplementary Figure 6. Structure determination of Zn^{2+}-bound ERp44

(A) An asymmetric unit contains four ERp44 molecules and a total of ten strong anomalous peaks from the bound Zn^{2+} ions. Anomalous difference Fourier map at 10 σ in the crystallographic asymmetric units are shown in magenta.

(B) Initial SAD-phased electron density map in the neighborhood of the His-cluster at 1 σ. Anomalous difference Fourier map at 15 σ is also shown in magenta.

(C) The overall structure of the Zn^{2+}-bound ERp44 homodimer is shown in a putty representation,

with the tube radius increasing from low to high B-factor.
(D) Comparison of the C-tail conformation. The **b'** domain (white) and C-tail (magenta) of the four independent ERp44 molecules in the asymmetric unit are shown in a putty representation, with the tube radius increasing from low to high B-factor.
(E, F) Close-up views of the conformation of the C-terminus of Mol A (E) and Mol B (F). Dashed lines represent disordered residues.
(G) Close-up view of the interactions formed at the Zn^{2+}-bound His-cluster (site 1). Coordination to Zn^{2+} and van der Waals contacts are represented by yellow and green dashed lines, respectively.

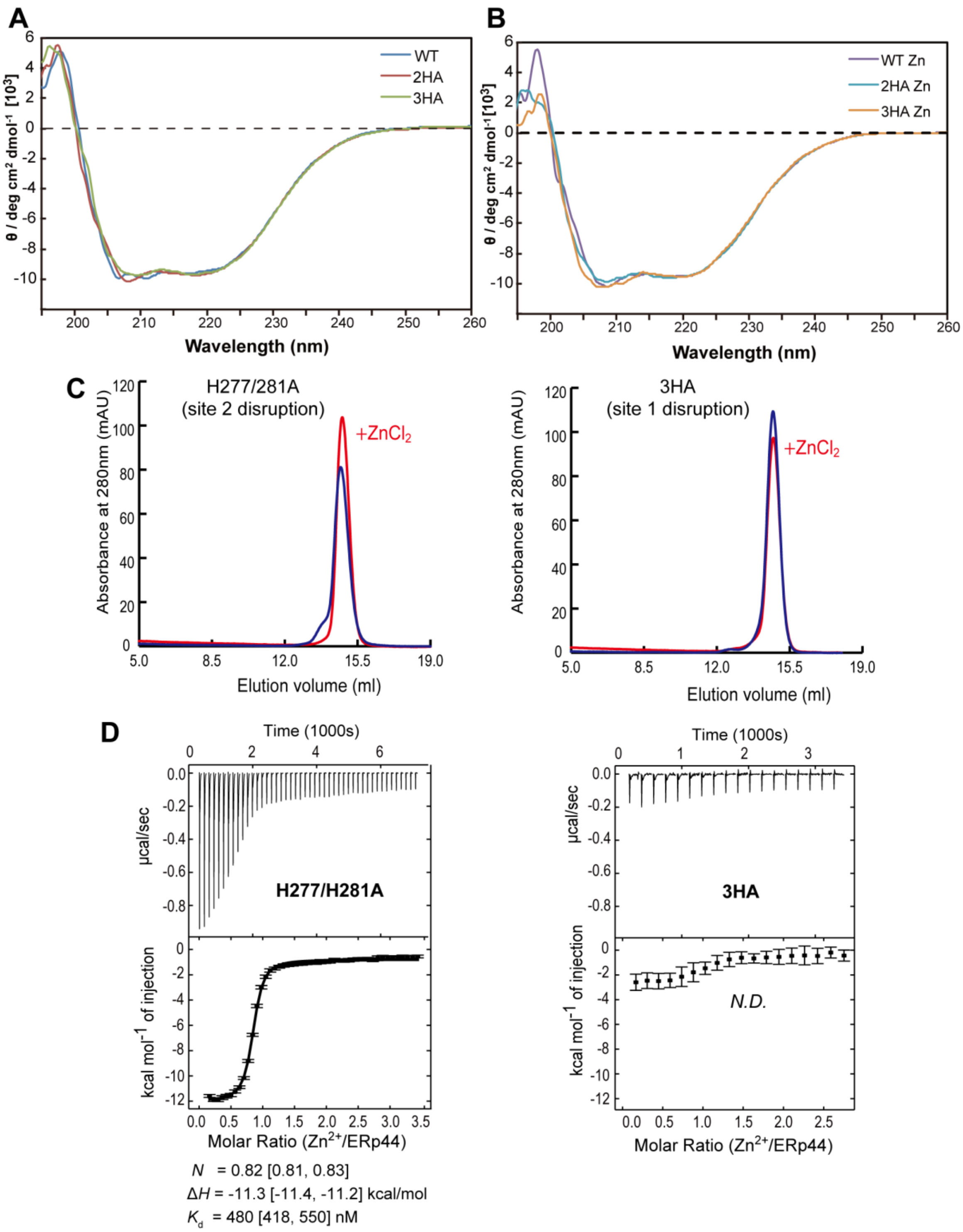

Supplementary Figure 7. Biophysical characterization of Zn^{2+}-binding deficient mutants of ERp44

(A, B) Far-UV CD spectra of ERp44 WT, 2HA (His277/281Ala) and 3HA (His299/328/332Ala) mutants in the absence (A) or presence of Zn^{2+} (B).

(C) SEC analysis for ERp44 H277/281A and 3HA (H299/328/332A) in the presence (red trace) or absence (blue trace) of Zn2+.

(D) ITC raw data (upper) and binding isotherm data (lower) for titration of $ZnCl_2$ into ERp44 H277/281A (60µM) and 3HA (H299/328/332A) (30µM)

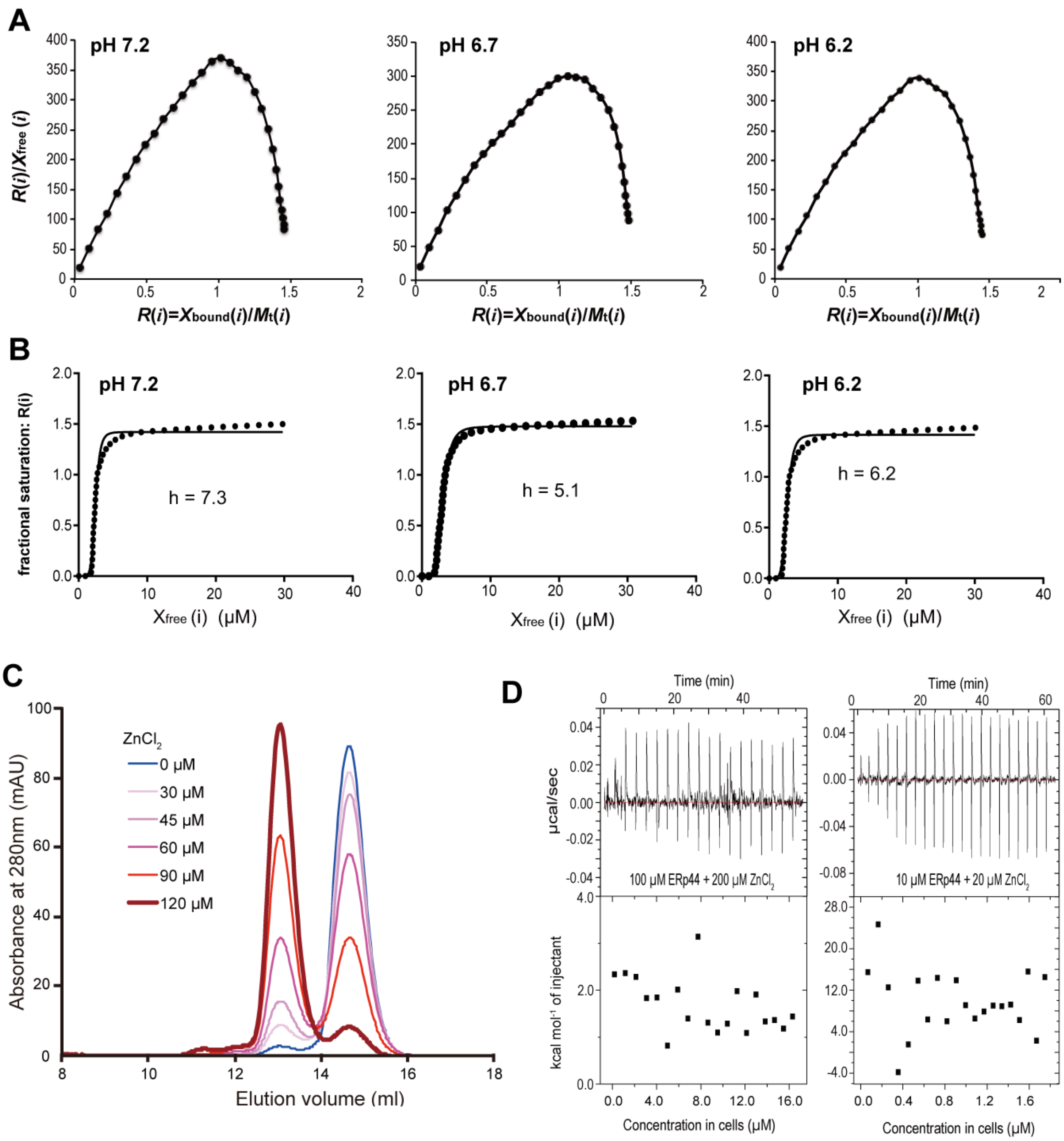

Supplementary Figure 8. Positive cooperativity in Zn^{2+} binding to site 1 and site 2.

(A). Scatchard plots calculated based on the observed ITC data shown in Fig. S1A. In the plots, the fraction of bound Zn^{2+} versus total ERp44 ($R(i) = X_{\mathrm{bound}}(i) / M_t(i)$) is plotted against $R(i) / X_{\mathrm{free}}(i)$, where $X_{\mathrm{bound}}(i)$ is the concentration of Zn^{2+} bound to ERp44 after the i-th injection, $M_t(i)$ is the total concentration of ERp44 after the i-th injection, and $X_{\mathrm{free}}(i)$ is the concentration of free Zn^{2+} after the i-th injection. Note that the observed concave downward curves indicate positive cooperativity in Zn^{2+} binding to sites 1 and 2.

(B) Saturation analysis based on the observed ITC data, where X_{free} is plotted against fractional saturation ($R(i) = X_{\mathrm{bound}}(i) / M_t(i)$). Calculated saturation curves with Hill coefficients are represented by solid lines.

(C) SEC analysis of the Zn^{2+}-dependent dimerization of ERp44 (60 μM) in the presence of indicated concentrations of Zn^{2+} at pH 6.7.

(D) ITC raw data (upper) and binding isotherm data (lower) for 100-fold dilution of the mixture of (left) 100μM ERp44 and 200 μM $ZnCl_2$ or (right) 10 μM ERp44 and 20 μM $ZnCl_2$ into buffer (20 mM BisTris pH6.2, 150 mM NaCl).

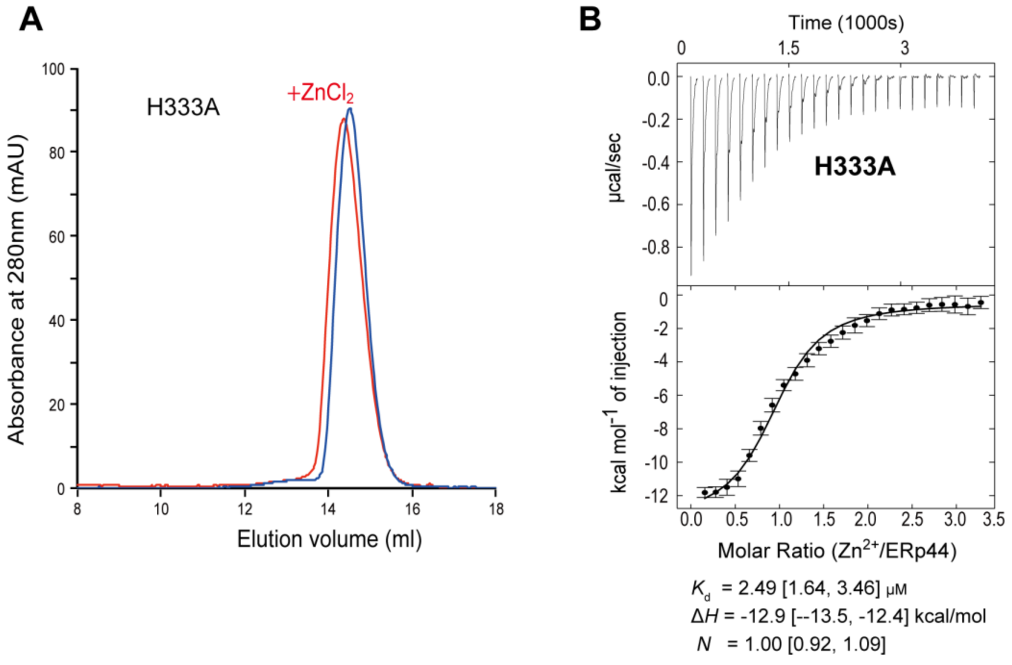

Supplementary Figure 9. Biophysical characterization of site 3 mutant of ERp44

(A) SEC analysis for ERp44 H333A in the presence (red trace) or absence (blue trace) of Zn^{2+}.

(B) ITC raw data (upper) and binding isotherm data (lower) for titration of $ZnCl^{2+}$ (0.5 mM) into ERp44 H333A (30µM).

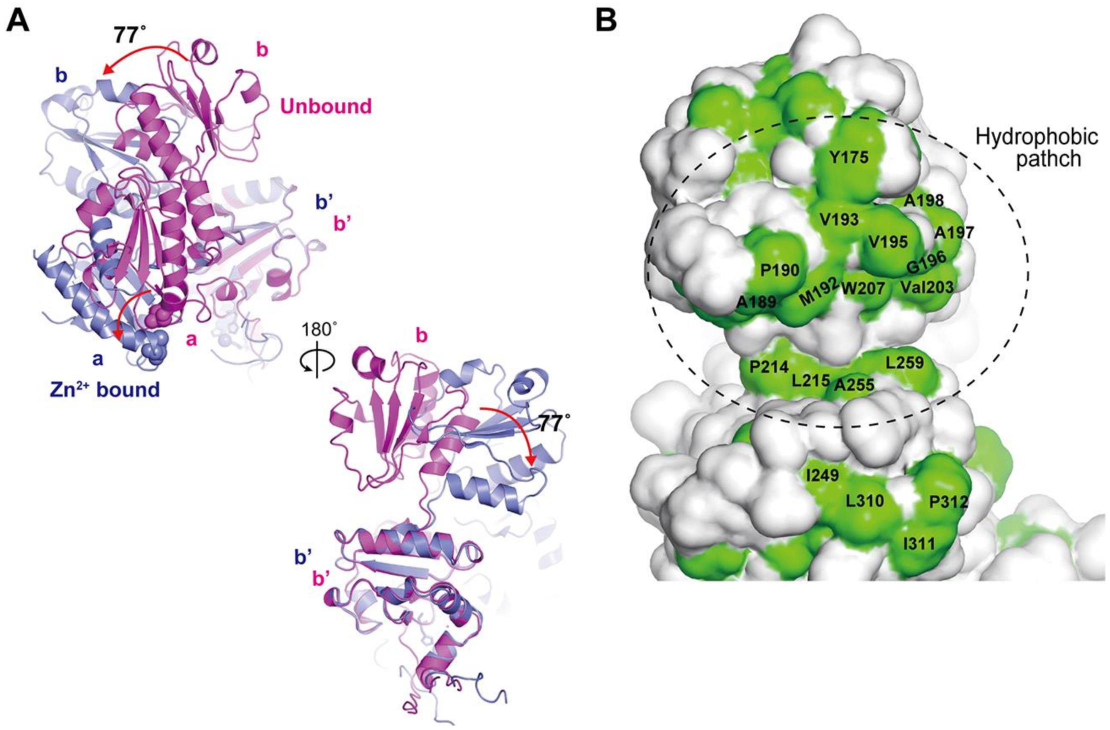

Supplementary Figure 10. Zn^{2+}-dependent conformational changes of ERp44

(A) Superposition of the unbound (magenta) and Zn^{2+} bound forms (blue) of ERp44. The **b**' domains of the two forms are superimposed on each other.

(B) Close-up view of the hydrophobic patch of Zn^{2+}-bound ERP44 (Fig. 4B). Residues involved in the hydrophobic patch are indicated in green.

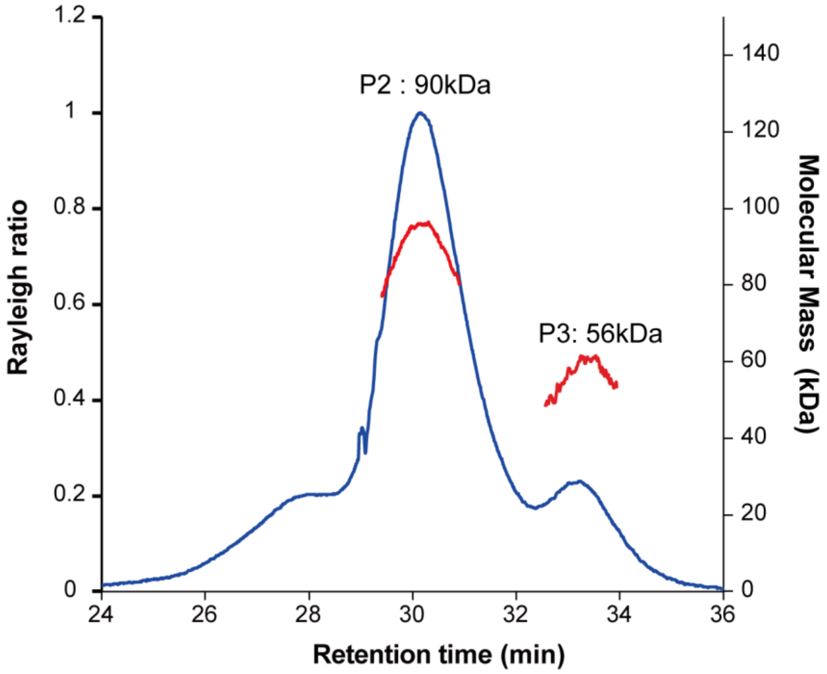

Supplementary Figure 11. Zn^{+2}-bound dimer of ERp44 can dissociate to form the heterodimer with Ero1α

SEC-MALS analysis for the mixture of ERp44 (60 µM), $ZnCl_2$ (120 µM) and lastly Ero1α (60 µM) was performed at room temperature. A SEC profile of the mixture is shown by a blue line. Averaged molecular masses calculated by MALS are indicated by red lines for the peak fractions, P2 and P3.

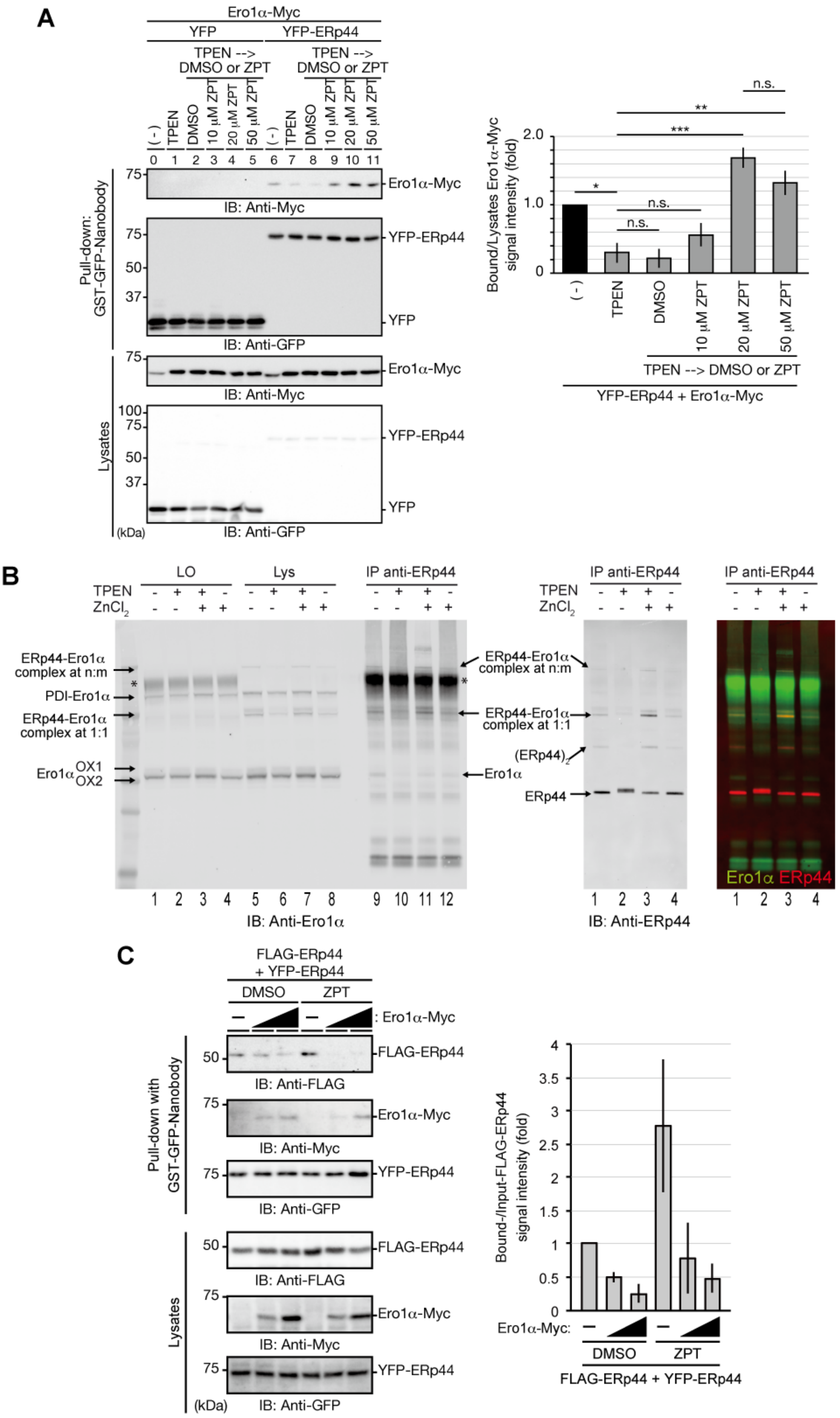
A
Ero1α-Myc
YFP
YFP-ERp44
TPEN --> DMSO or ZPT
(-)
TPEN
DMSO
10 μM ZPT
20 μM ZPT
50 μM ZPT
0 1 2 3 4 5 6 7 8 9 10 11
Pull-down: GST-GFP-Nanobody
Lysates
IB: Anti-Myc
IB: Anti-GFP
YFP-ERp44
YFP
(kDa)
Bound/Lysates Ero1α-Myc signal intensity (fold)
n.s.
*
**
***
YFP-ERp44 + Ero1α-Myc
B
LO
Lys
IP anti-ERp44
TPEN
ZnCl2
ERp44-Ero1α complex at n:m
PDI-Ero1α
ERp44-Ero1α complex at 1:1
Ero1α OX1 OX2
(ERp44)2
Ero1α
ERp44
IB: Anti-Ero1α
IB: Anti-ERp44
Ero1α ERp44
C
FLAG-ERp44 + YFP-ERp44
DMSO
ZPT
: Ero1α-Myc
Pull-down with GST-GFP-Nanobody
FLAG-ERp44
IB: Anti-FLAG
Bound-/Input-FLAG-ERp44 signal intensity (fold)
Ero1α-Myc:

Supplementary Figure 12. Zn^{2+} affects the formation of ERp44-Ero1α complexes in cells

(A) Pull-down assay for Zn^{2+}-dependent complex formation between overexpressed ERp44 and Ero1α. HeLa cells co-transfected with Ero1α-Myc and YFP-Mock or YFP-ERp44 were treated with 10 µM TPEN for 30 min and then subsequently with 10-50 µM ZPT for 15 min. Cell lysates were subjected to immunoprecipitation with GST-GFP-Nanobody. The right panel shows the level of ERp44-bound Ero1α-Myc normalized against the level of input Ero1α-Myc. Data are the means ± SEM (N=3, one-way ANOVA followed by Tukey's test). ***, $p < 0.001$; **, $p < 0.01$; *, $p < 0.05$; n.s., not significant ($p > 0.05$).

(B) Co-immunoprecipitation of endogenous ERp44-Ero1α complexes. Lysates from HeLa cells were subjected to immunoprecipitation with anti-ERp44 (36C9), and the immunoprecipitates (IP), leftovers (LO) and input lysates (Lys) analyzed by immunoblotting under non-reducing conditions on 4-12% polyacrylamyde gradient gels. After transfer, the nitrocellulose filter was sequentially decorated with a rabbit anti-ERp44 polyclonal antibody followed by and anti-rabbit Alexa 700 secondary antibody, and subsequently with a mouse monoclonal anti-Ero1α antibody (2G4) followed by anti-mouse Alexa 488 antibodies. Clearly, Ero1α is associated with ERp44 non-covalently (see the ~65 kDa band) as well as covalently (see the higher molecular weight bands, stabilized by addition of $ZnCl_2$). The panel on the right shows an overlay of the two signals, that highlights the ERp44-Ero1α covalent complexes (yellow bands). Note that Zn^{2+} depletion by TPEN treatment impaired the formation of both covalent and non-covalent ERp44-Ero1α complexes. Note also the slower mobility of intracellular ERp44 in TPEN treated cells (lanes 2 in the right panels), reflecting O-glycosylation in the Golgi.

(C) Pull-down assay for detection of the ERp44 homodimer or the ERp44-Ero1α binary complex formed in cells. HeLa cells co-expressing FLAG-tagged ERp44, YFP-ERp44 and Ero1α-Myc were treated with DMSO or 2.5 µM ZPT for 15 min. Cell lysates were subjected to pull-down assay using GST-GFP-Nanobody and immunoblotted with indicated antibodies. The signal intensity of co-precipitated FLAG-ERp44 relative to that of whole FLAG-ERp44 in cell lysates was quantified, and the data are shown in the right graph. Data are the means ± SEM (N=3).

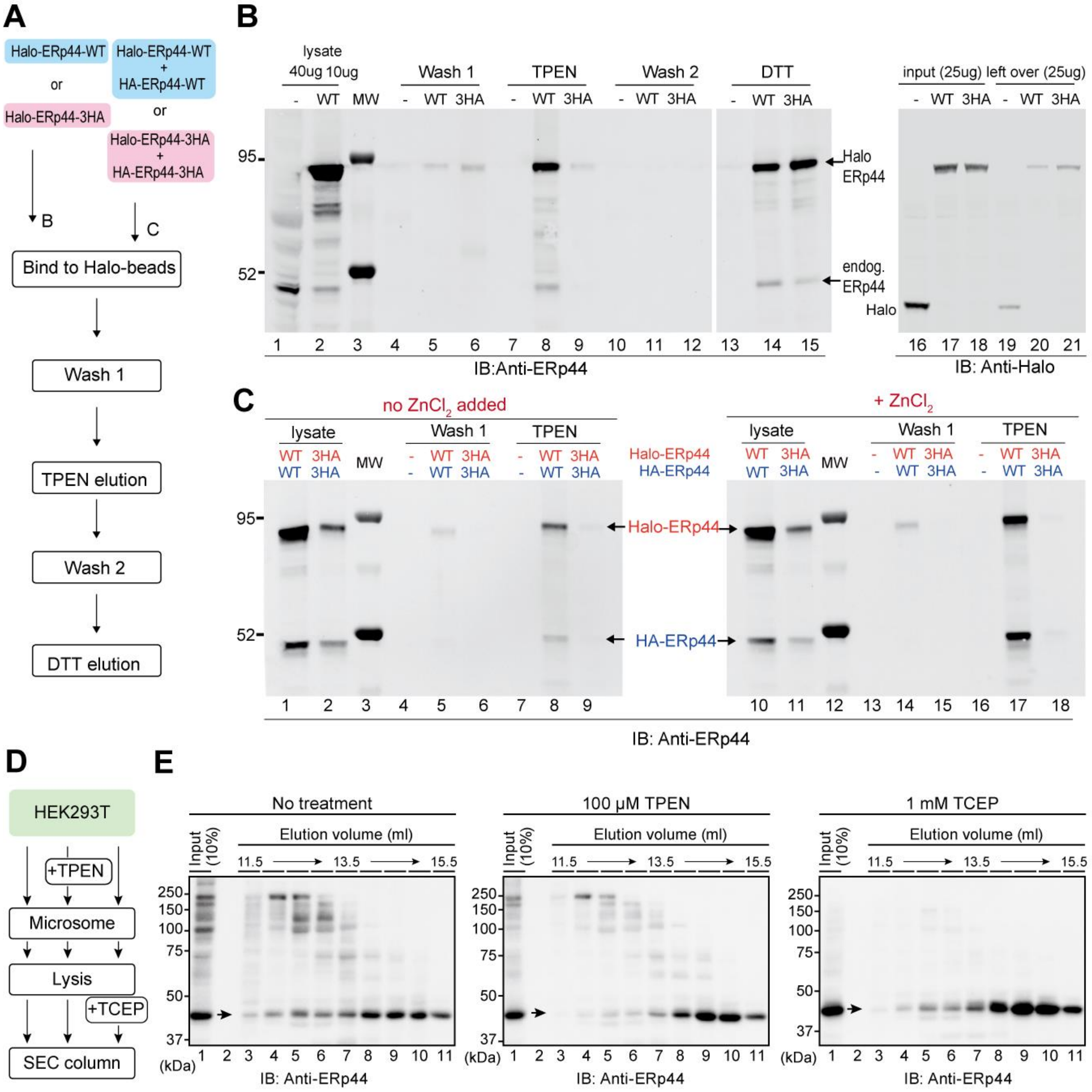

Supplementary Figure 13. Existence of Zn^{2+}-bridged homodimers of ERp44 in cells

(A) Flowcharts of sequential elution assays shown in panels B and C.

(B) Presence of Zn^{2+}-dependent and covalent homodimers (oligomers) in living cells. HepG2 cells were transfected with Halo-ERp44 WT or 3HA, or an ER resident Halo alone (-) as a control, and lysed and handled in solutions supplemented with $ZnCl_2$. Aliquots of post-nuclear supernatants corresponding to 1 mg of total protein were incubated with immobilized Halo ligands. Beads were washed several times before the first elution with TPEN, washed again and then eluted with DTT and SDS (lane 4-15). Aliquots of total lysates from Halo or WT Halo-ERp44 expressing cells (40 or 10 μg) were analyzed to obtain an indicative quantification of endogenous ERp44 (lane 1-2). Aliquots (25 μg) of the lysates before and after precipitation with the Halo-ligand beads were decorated with anti-Halo to validate precipitation (lanes 16-21).

(C) HepG2 cells co-transfected with Halo-ERp44 and HA-ERp44 (both WT or both 3HA) were handled as above, using buffers without (left panel) or with (right panel) added $ZnCl_2$. Clearly, the presence of Zn^{2+} stabilizes the non-covalent complexes involving Halo-ERp44 WT. Importantly, however, significant portion of Halo- and HA-ERp44 was co-precipitated also even without added $ZnCl_2$. In contrast, little if any Halo- or HA-ERp44 3HA is eluted by TPEN from beads covered with the 3HA mutants.

(D) Flowchart of the SEC assay shown in panel E.

(E) SEC analysis for the microsomal lysate of HEK293T cells. Each fraction was analyzed by non-reducing immunoblotting with D17A6 anti-ERp44 antibodies. Arrows indicate a possible non-covalent homodimer of endogenous ERp44. The lysate was treated with TPEN (middle) and TCEP (right), respectively.

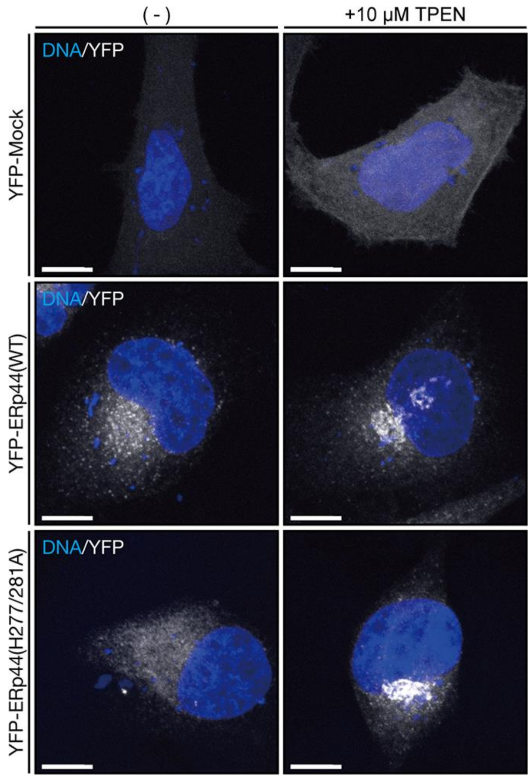

Supplementary Figure 14. ERp44 dimerization is not essential for exit from the ER to the Golgi. HeLa cells transfected with YFP-ERp44 (WT or H277/281A) or YFP-Mock were treated with 10 μM TPEN/Opti-MEM for 2 h and fixed with 4% PFA/PBS. Nuclei were stained with DAPI. Fluorescent images were obtained with laser scanning confocal microscopy. Scale bars, 10 μm. Like ERp44 (WT), the dimerization-defective mutant (H277/281A) accumulates in the Golgi area after TPEN treatment.

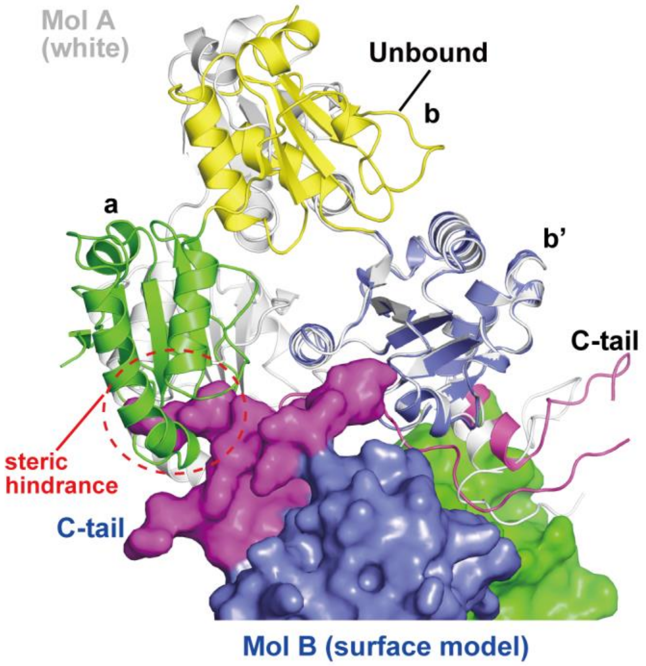

Supplementary Figure 15. Structural rationalization for the sequential Zn^{2+} binding to ERp44. Superposition of metal-free monomer and Zn^{2+}-bound dimer of ERp44. The **b**' domains of these two states are superimposed to each other. The structure of Zn^{2+}-free ERp44 monomer is represented by a ribbon diagram and colored in green (**a** domain), yellow (**b** domain), blue (**b'** domain) and magenta (C-tail). For the Zn^{2+}-bound dimer, one protomer is shown in white, while the other is represented by surface model and colored in the same way as indicated above.

Supplementary Table 1 Sequences of siRNAs used for ZnTs knockdown

Name	Target Gene	siRNA ID	Sense sequence (5'-3')	Antisense sequence (5'-3')
siZnT5 #1	SLC30A5	S219	CGAAUCAUAUGAUCUCCUATT	UAGGAGAUCAUAUGAUUCGAA
siZnT5 #2	SLC30A5	S220	GCUCGAUUAACAAAAUAUATT	UAUAUUUUGUUAAUCGAGCGC
siZnT6 #1	SLC30A6	S31227	CUUUUCAGAUCAUCACGUATT	UACGUGAUGAUCUGAAAAGTT
siZnT6 #2	SLC30A6	S31229	CGAUGCUUUCUAUUCGGAATT	UUCCGAAUAGAAAGCAUCGTG
siZnT7 #1	SLC30A7	S45226	GCUUAGGCUUGAUUUCCGATT	UCGGAAAUCAAGCCUAAGCAG
siZnT7 #2	SLC30A7	S45225	CAGAUACACUUGGAAGUAUTT	AUACUUCCAAGUGUAUCUGCT

Supplementary Table 2. Primers used for qRT-PCR experiments shown in Supplementary Fig. 4F and G

Gene	Name	Sequence (5'-3')
SLC30A5	ZnT5 forw	GGTTCTACTCCTGAGATTGC
SLC30A5	ZnT5 rev	AATGAGGGTCTCGGTATGAT
SLC30A6	ZnT6 forw	CTGAACTGCCGTGTCCTGAG
SLC30A6	ZnT6 rev	ACCTTTGGTGGGACAGATGC
SLC30A7	ZnT7 forw	CATGGTGCTGCACATAGCCA
SLC30A7	ZnT7 rev	AGGACGGGCCATCATGAGAA
GAPDH	GAPDH fow	TGAAGGTCGGAGTCAACGGATTT
GAPDH	GAPDH rev	CATGTAAACCATGTAGTTGAGGT

Supplementary Table 3. Primers used for qRT-PCR experiments shown in Supplementary Fig. 4E

Gene	Name	Sequence (5'-3')
SLC30A5	hZNT5 129Fw RT-PCR	GGCTGTGGGACTTTTCGAATC
SLC30A5	hZNT5 307Rv RT-PCR	ACCCAGCAACTGCATGTTTA
SLC30A6	hZNT6 81Fw RT-PCR	TGACCGAAGGTCCTGGAAGA
SLC30A6	hZNT6 160Rv RT-PCR	AACTGCACCACATAAGCAGGA
SLC30A7	hZNT7 5Fw RT-PCR	TGCCCCTGTCCATCAAAGAC
SLC30A7	hZNT7 189Rv RT-PCR	GCCTAAGCAGTTGCTCCAGA
ACTB	hACTB 224Fw RT-PCR	TCGTCACCAACTGGGACGAC
ACTB	hACTB 405Rv RT-PCR	AGCAACGTACATGGCTGGGG

Supplementary Table 4. Primers used to construct the ERp44 mutants in this work

Name	Sequence (5'-3')
ERp44H299A1	ATTGACAGCTTTAGGGCCATGTATGTGTTTGGA
ERp44H299A2	TCCAAACACATACATGGCCCTAAAGCTGTCAAT
hERp44(H328,332A) fw	CTGGAAAACTGGCCAGAGAATTCGCTCATGGACCTG
hERp44(H328,332A) rv	CAGGTCCATGAGCGAATTCTCTGGCCAGTTTTCCAG
hERp44_H277/281A_fw PrimeSTAR	GCTCCTCTTCTGGCCATACAGAAAACTCCAGCA
hERp44_H277/281A_rv PrimeSTAR	GGCCAGAAGAGGAGCTCTAAATTTGTCACAATC

Supplementary Table 5. Data collection and phasing and refinement statistics for the merged data

**Data collection**	Merged data set
Space group	$P6_122$
Cell dimensions	
$a = b$ (Å)	175.15
c (Å)	407.81
Resolution (Å)[a]	44– 2.45 (2.51-2.45)
Refs. total/unique	8766656 / 135470
Number of dataset	7
Completeness (%)[a]	100.0 (100.0)
Redundancy	64.7
$I/\sigma(I)$[a]	33.1 (5.2)
R_{meas} (%)	15.7 (110)
$CC_{1/2}$	1.000 (0.582)
**Phasing**	
CC_{all}/CC_{weak} (%)	41.48/21.81
FOM after SHELXE	0.652
CC for auto-tracing in SHELXE (%)	29.3
**Refinement**	
R_{work}/R_{free} (%)	18.4/21.4
No. atoms	
Protein	11404
Ion (Zn^{2+}, Cl^-)	18
Water	527
Average B-factor	
Protein	61.53
Water	52.58
R.m.s. deviations	
bond length (Å)	0.009
bond angles (˚)	0.927
Ramachandran plot	
favored (%)	97.5
allowed (%)	2.5
outliers (%)	0.0

[a]Values in parentheses are for the high-resolution shell.

[b]$R_{meas} = \Sigma_{hkl} \{n/[n-1]\}^{1/2} \Sigma_i|I_i(hkl) - \langle I(hkl)\rangle| / \Sigma_{hkl}\Sigma I_i(hkl)$, where $I_i(hkl)$ is the ith observed intensity and $\langle I(hkl)\rangle$ is the average intensity from multiple observations

Supplementary Table 6. Data collection statistics of each data set for merging

Data set	1	2	3	4	5	6	7	1 to 7
Wavelength (Å)	1.1000	1.1000	1.1000	1.1000	1.1000	0.9000	0.9000	
Cell dimensions								
a = *b* (Å)	174.99	175.148	174.83	175.70	175.26	175.10	174.88	175.15
c (Å)	407.34	407.81	407.37	408.77	407.95	407.88	407.36	407.81
Oscillation rage (˚)	80	80	80	80	80	80	80	
Resolution (Å)	49-2.45	49-2.45	49-2.45	49-2.45	49-2.45	49-2.45	49-2.45	49-2.45
(outer shell)	(2.51-2.45)	(2.51-2.45)	(2.51-2.45)	(2.51-2.45)	(2.51-2.45)	(2.51-2.45)	(2.51-2.45)	(2.51-2.45)
Refs. total	1244203	1243315	1242069	1254917	1256589	1184144	1342953	8766656
Refs. unique	129494	135193	128735	136496	135642	100952	98120	135470
Completeness (%)[a]	95.86	99.79	95.47	99.92	99.96	74.55	72.72	100.00
(outer shell)	(97.61)	(99.0)	(97.31)	(100.00)	(99.99)	(73.93)	76.03	99.98
Redundancy	9.6	9.2	9.7	9.2	9.3	11.7	13.7	64.7
$I/\sigma(I)$[a]	15.8	14.2	11.1	14.3	13.3	19.7	12.2	31.0
(outer shell)	(2.0)	(1.8)	(1.3)	(2.3)	(1.8)	(2.1)	(2.5)	(5.1)
R_{meas} (%)	12.2	15.1	17.2	14.3	15.8	8.5	15.0	15.7
(outer shell)	(105.1)	(119)	(148.8)	(95.3)	(1.23)	(76.7)	(103.7)	(110.5)
$CC_{1/2}$	0.997	0.998	0.995	0.999	0.998	0.999	0.996	1.000
(outer shell)	(0.198)	(0.156)	(0.148)	(0.106)	(0.144)	(0.228)	(0.327)	(0.582)
CC with data set 1		0.991	0.991	0.986	0.989	0.991	0.989	
(at 3.0-2.9Å resolution)		(0.870)	(0.871)	(0.846)	(0.871)	(0.919)	(0.9197)	

Supplementary Table 7. List of B-factors (Å^2) of Zn^{2+} and Cl^{-1} ions

	Zn1	Zn2	Zn3	Cl1	C12
Mol A	45.77		46.00	53.39	57.13
(bridge)		72.39			
Mol B	43.58		48.89	56.59	62.19
Mol C	50.98		65.39	75.59	78.32
(bridge)		80.78			
Mol D	53.71		50.57	58.16	60.06

Supplementary Table 8. Zn^{2+} binding geometries in ERp44

**Site 1**	MolA	MolB	MolC	Mol D
Zn1-His299 (Å)	2.04	2.07	2.07	2.05
Zn1-His328 (Å)	2.09	2.04	2.09	2.09
Zn1-His332(Å)	2.00	2.09	2.00	2.00
Zn1-HOH (Å)	2.9	2.66	2.28	2.69
H299-Zn1-H328 (˚)	89.8	95.6	88.3	93.4
H299-Zn1-H332 (˚)	89.2	94.6	92.5	95.7
H328-Zn1-H332 (˚)	83.2	94.6	93.3	87.5
**Site 2**				
Zn2-His277 (Å)	1.94	2.07	2.05	2.07
Zn-His281 (Å)	2.00	2.05	2.04	2.07
His277-Zn2-His281(intra) (˚)	101.6	130.8	113.6	121.8
His281-Zn2-His281 (inter) (˚)	114.2		117.3	
His277-Zn2-His277 (inter) (˚)	93.3		73.4	
His277-Zn2-His281(inter) (˚)	108.6		104.6	
His277-Zn2-His281(inter) (˚)	103.1		115.9	
**Site 3**				
Zn3-His333 (Å)	1.92	1.88	1.93	2.02
Zn3-Cys29 (Å)	2.22	2.18	2.1	2.23
Zn3-Tyr78 (Å)	2.5	2.29	2.35	2.37
Zn3-Cl1 (Å)	2.97	2.9	2.97	2.91
Zn3-Cl2 (Å)	3.11	2.82	2.98	3.06
Tyr78-Zn3-His333 (˚)	82.6	98.0	90.9	76.8
Cys29-Zn3-His333 (˚)	117.9	124.4	131.05	107.6
Cys29-Zn3-Tyr78 (˚)	85	75.2	80.7	77.7
