# Supplementary material for: Zinc regulates ERp44-dependent protein quality control in the early secretory pathway
Source: Nat Commun. 2019 Feb 5;10:603. doi: 10.1038/s41467-019-08429-1 (PMC6363758; doi:10.1038/s41467-019-08429-1)
Supplement: Supplementary file 3 — Description of Additional Supplementary Files [file 41467_2019_8429_MOESM3_ESM.docx]

**Description of Additional Supplementary Files**

File Name: Supplementary movie 1

Description: Fluorescent time-lapse images of HeLa cells expressing YFP-ERp44. Cells were treated with 10 µM TPEN and subsequently DMSO at T = 2 min and 32 min, respectively, related to Fig. 1g (upper panel).

File Name: Supplementary movie 2

Description: Fluorescent time-lapse images of HeLa cells expressing YFP-ERp44. Cells were treated with 10 µM TPEN and subsequently 20 µM ZPT at T = 2 min and 32 min, respectively, related to Fig. 1g (lower panel).

File Name: Supplementary movie 3

Description: Zn2+-induced conformational changes of the His-cluster and striking domain rearrangements of ERp44.
