# Supplementary material for: Zinc regulates ERp44-dependent protein quality control in the early secretory pathway
Source: Nat Commun. 2019 Feb 5;10:603. doi: 10.1038/s41467-019-08429-1 (PMC6363758; doi:10.1038/s41467-019-08429-1)
Supplement: Supplementary file 8 — Reporting Summary [file 41467_2019_8429_MOESM8_ESM.pdf]

nature research

Corresponding author(s): Kenji Inaba, Roberto Sitia

Reporting Summary

Nature Research wishes to improve the reproducibility of the work that we publish. This form provides structure for consistency and transparency in reporting. For further information on Nature Research policies, see Authors & Referees and the Editorial Policy Checklist.

Statistical parameters

When statistical analyses are reported, confirm that the following items are present in the relevant location (e.g. figure legend, table legend, main text, or Methods section).

n/a	Confirmed	
☐	☒	The exact sample size (*n*) for each experimental group/condition, given as a discrete number and unit of measurement
☐	☒	An indication of whether measurements were taken from distinct samples or whether the same sample was measured repeatedly
☐	☒	The statistical test(s) used AND whether they are one- or two-sided<br>*Only common tests should be described solely by name; describe more complex techniques in the Methods section.*
☒	☐	A description of all covariates tested
☐	☒	A description of any assumptions or corrections, such as tests of normality and adjustment for multiple comparisons
☐	☒	A full description of the statistics including central tendency (e.g. means) or other basic estimates (e.g. regression coefficient) AND variation (e.g. standard deviation) or associated estimates of uncertainty (e.g. confidence intervals)
☐	☒	For null hypothesis testing, the test statistic (e.g. *F*, *t*, *r*) with confidence intervals, effect sizes, degrees of freedom and *P* value noted<br>*Give P values as exact values whenever suitable.*
☒	☐	For Bayesian analysis, information on the choice of priors and Markov chain Monte Carlo settings
☒	☐	For hierarchical and complex designs, identification of the appropriate level for tests and full reporting of outcomes
☐	☒	Estimates of effect sizes (e.g. Cohen's *d*, Pearson's *r*), indicating how they were calculated
☐	☒	Clearly defined error bars<br>*State explicitly what error bars represent (e.g. SD, SE, CI)*

Our web collection on statistics for biologists may be useful.

Software and code

Policy information about availability of computer code

Data collection	BSS (Beamline Scheduling Software) and UGUI (Unified Graphical User Interface) are used for X-ray data collection in SPring-8 and Photon factory, respectively.
Data analysis	We used XDS and XSCALE for the processing of X-ray diffraction data, SHELXCDE for SAD phasing, Buccaneer for auto-model building, COOT for manual model building, PHENIX for structure refinement, MolProbity for structure validation, PyMOL and Chimera for structural figures. We also used NITPIC for integration of the ITC peaks and SEDPHAT for global analysis of the ITC data.<br>The value of Pearson's corelation coefficient were obtained by ImageJ Version 1.49K or FIJI 2.0 using JACoP Plugin (https://imagej.nih.gov/ij/plugins/track/jacop.html). Statistical analyses including two-tailed unpaired t-test and one-way ANOVA followed by Dunnett's test or Tukey's test were performed with GraphPad Prism version 7.0a.

For manuscripts utilizing custom algorithms or software that are central to the research but not yet described in published literature, software must be made available to editors/reviewers upon request. We strongly encourage code deposition in a community repository (e.g. GitHub). See the Nature Research guidelines for submitting code & software for further information.

Data

Policy information about availability of data

All manuscripts must include a data availability statement. This statement should provide the following information, where applicable:

- Accession codes, unique identifiers, or web links for publicly available datasets
- A list of figures that have associated raw data
- A description of any restrictions on data availability

All data generated or analysed during this study are included in this article and its supplementary information files. Coordinates and structure factors of crystal structure of Zn2+-bound ERp44 (shown in Fig. 3) have been deposited in the Protein Data Bank with accession code 5XWM.

Field-specific reporting

Please select the best fit for your research. If you are not sure, read the appropriate sections before making your selection.

☒ Life sciences ☐ Behavioural & social sciences ☐ Ecological, evolutionary & environmental sciences

For a reference copy of the document with all sections, see nature.com/authors/policies/ReportingSummary-flat.pdf

Life sciences study design

All studies must disclose on these points even when the disclosure is negative.

Sample size	No statistical methods were used to predetermine sample size. The sample size was chosen based on previous experience for each experiment to yield high power to detect specific effects.
Data exclusions	No data were excluded in all statistical analyses performed in this study.
Replication	We confirmed that all data shown in this paper were reproducible.
Randomization	Randomization is not relevant to this work since we have not allocated any samples into experimental groups.
Blinding	Blinding was not relevant to this work since we have not done group allocation in any experiments.

Reporting for specific materials, systems and methods

Materials & experimental systems

n/a	Involved in the study	
☐	☒	Unique biological materials
☐	☒	Antibodies
☐	☒	Eukaryotic cell lines
☒	☐	Palaeontology
☒	☐	Animals and other organisms
☒	☐	Human research participants

Methods

n/a	Involved in the study	
☒	☐	ChIP-seq
☒	☐	Flow cytometry
☒	☐	MRI-based neuroimaging

Unique biological materials

Policy information about availability of materials

Obtaining unique materials	purified human ERp44 protein, purified human Ero1a protein,

Antibodies

Antibodies used	Mouse monoclonal antibodies; Anti-c-Myc-HRP conjugated (Western blotting, Santa Cruz, sc40 HRP, clone 9E10, lot# F1114), Anti-c-Myc (Immunofluorescence, Santa Cruz, sc40, clone 9E10, lot# I2217), Anti-FLAG-Peroxidase (Western blotting, SIGMA, A8592, clone M2), Anti-PDI (Immunofluorescence, Enzo, ADI-SPA-891, clone 1D3), Anti-ERp44 (Western blotting, clone 36C9), Anti-ERp44 (Immunofluorescence, clone 2D5), Anti-FLAG (Western blotting, SIGMA, clone M2 lot# SLBT765) Anti-HA (Western blotting, clone 12CA5, in house), Anti-Myc (Western blotting, clone 9E10, in house), Anti-ERO1alpha (Western blotting, clone

2G4 in house)
Rabbit monoclonal antibodies; Anti-ERp44 (Western blotting, Cell Signaling Technology, #3798, clone D17A6, lot# 2), Anti-Rab7 (Immunofluorescence, Cell Signaling Technology, #9367, clone D95F2, lot# 1)
Rabbit polyclonal antibodies; Anti-Calnexin (Immunofluorescence, Enzo, ADI-SPA-860, lot# 05031014), Anti-EEA1 (Immunofluorescence, MBL, PM062, Lot# 003), Anti-ERGIC53 (Immunofluorescence, SIGMA, E1031, lot# 024M4845), Anti-GFP (Western blotting, Molecular Probes, A-6455, lot# 1964399), Anti-GM130 (Immunofluorescence, MBL, PM061, lot# 003), Anti-PDI (Western blotting, clone A66 kind gift of Dr. Ineke Braakman, Utrecht, NL), Anti-HALO (Western blotting, Promega, ref G928A, lot#0000196948) Anti-GM130 (Immunofluorescence clone N309, kind gift of A. De Matteis, Naples, I), Anti-ERp44 (Western blotting, proteintech cat.n 16016-I-AP)

Validation

Mouse monoclonal antibodies:
Anti-c-Myc; supplier (https://www.scbt.com/scbt/ja/product/c-myc-antibody-9e10)
Anti-FLAG; supplier (https://www.sigmaaldrich.com/catalog/product/sigma/f1804?lang=ja&region=JP)
Anti-PDI; supplier (http://www.enzolifesciences.com/ADI-SPA-891/pdi-monoclonal-antibody-1d3/)
Anti-ERp44 (clone 36C9, 2D5); Anelli T. et al., EMBO J., 26, 4177-4188, 2007
Anti flag; supplier (https://www.sigmaaldrich.com/catalog/product/sigma/f1804?lang=it&region=IT)
Anti-ERO1alpha (2G4) Ronzoni et al., traffic 11:947–957, 2010
Anti-Myc (9E10, in house purified from 9E10.2 clone ATCC CRL-1729)
Anti-HA (12CA5 in house purified from 12CA5 hybridoma supernatant ATCC)
Rabbit monoclonal antibodies:
Anti-ERp44; supplier (https://www.cellsignal.com/products/primary-antibodies/erp44-d17a6-xp-rabbit-mab/3798)
Anti-Rab7; supplier (https://www.cellsignal.jp/products/primary-antibodies/rab7-d95f2-xp-rabbit-mab/9367)
Rabbit polyclonal antibodies:
Anti-Calnexin; supplier (http://www.enzolifesciences.com/ADI-SPA-860/calnexin-polyclonal-antibody/)
Anti-EEA1; supplier (http://ruo.mbl.co.jp/bio/dtl/A/index.html?pcd=PM062)
Anti-ERGIC53; supplier (https://www.sigmaaldrich.com/catalog/product/sigma/e1031?lang=ja&region=JP)
Anti-GFP; supplier (https://www.thermofisher.com/antibody/product/GFP-Antibody-Polyclonal/A-6455)
Anti-GM130 (clone N309) Marra P et al., MBoC , 18, 1595–1608, May 2007
Anti-ERp44; supplier (https://www.ptglab.com/products/ERP44-Antibody-16016-1-AP.htm)
Anti Halo; supplier (https://ita.promega.com/Products/Imaging-and-Immunological-Detection/ELISAs-and-Antibodies/Anti_HaloTag-pAb/?fq=anti%20halo%20G928A&catNum=G9281)
Anti-PDI: (clone A66) Lambert N et al., Biochem J. Jul 1; 213(1): 225–234, 1983

Eukaryotic cell lines

Policy information about cell lines

Cell line source(s)	HeLa (subline1, RIKEN BRC, RCB0007), HeLa (subline2, ATCC-CCL2), HEK293T (ATCC, CRL-3216), HepG2 (ATCC, HB-8065)
Authentication	None of the cell lines used have been authenticated.
Mycoplasma contamination	The cell lines were not tested for mycoplasma contamination.
Commonly misidentified lines (See ICLAC register)	No commonly misidentified cell lines were used in this study.
