# Supplementary material for: Zinc regulates ERp44-dependent protein quality control in the early secretory pathway
Source: Nat Commun. 2019 Feb 5;10:603. doi: 10.1038/s41467-019-08429-1 (PMC6363758; doi:10.1038/s41467-019-08429-1)
Supplement: Supplementary file 2 — Peer Review File [file 41467_2019_8429_MOESM2_ESM.pdf]

Reviewers' comments:

Reviewer #1 (Remarks to the Author):

This manuscript describes the role of zinc in the localization and function of ERp44, a PDI family member that patrols the ER-Golgi boundary and retrieves proteins that have not yet completed oxidative folding/assembly and certain proteins that should be localized to the ER. While the pH gradient along the secretory pathway is well known, a zinc gradient is less widely appreciated. The authors convincingly demonstrate that zinc has an important role to play in regulation of the function of ERp44, on top of the role of pH that they had previously demonstrated. Their conclusions are supported by a wide variety of experiments including subcellular localization of ERp44 upon depletion of Golgi zinc by chelators and knockdown of zinc importers, zinc-bound ERp44 crystal structures, size exclusion chromatography, ITC, and secretion assays of client proteins. This comprehensive study, building upon previous insightful work, is summarized in an elegant mechanistic model.
The data are presented exceedingly clearly and aesthetically. The text is elegant and a pleasure to read.

This manuscript makes an important advance to the field of ER quality control and provides important lessons for others studying protein assembly, interactions, and the regulation of protein activity in the ER and Golgi. It should therefore be of broad interest and appeal.

Reviewer #2 (Remarks to the Author):

The paper by Watanabe et al., examines the role of Zn^{2+} binding in the structure and particularly function of the ERp44 protein. I have been asked by the editor to comment on the structure biology aspects of the paper. What follows below concentrates on that aspect, but also the Zn^{2+} binding analyses, given that is an additional area of my expertise. Overall, in assessing those aspects, I believe the paper will be suitable for publication in Nature Communications once the following issues are addressed:

The study found, by ITC that Zn^{2+} bound the protein at a molar ratio of 1.5:1 (Zn^{2+}:protein) and that metal binding also promoted its dimerization (non-covalent). I am convinced by this analysis. However, I feel the cooperativity between binding sites 1 and 2 is less convincing and should be revisited before the manuscript is suitable for publication. The authors should consider that ITC is not perhaps the ideal technique for distinguishing two binding sites with potentially different binding constants and coorperativity between them. A competition assay with a fluorescent Zn^{2+} complex or other spectrophotometrically-active ligand with a binding constant in the appropriate range may be able to distinguish the two binding events: the first to site 1, followed by site 2 and dimerization. FluoZin-3 is one such ligand, but a simple survey of the literature will reveal others. I think at the moment the coorperativity of binding is not underpinned appropriately by the experimental data presented.

The crystal structure observed three Zn^{2+} sites per protein – site 1 (3 x His + water), site 2 (at the interface; 4 x His) and site 3 (His, Cys, Tyr + 2xCl^-). The authors describe the binding geometries as tetrahedral. I would like to see coordination distances and angles included in the Supplementary Information for all three sites. This would give confidence in the quality in the refinement - that chemically-sensible sites have been modeled (His - Zn^{2+} coordination distances for example should be ~2.0-2.1 Å). This would be far more convincing than the Table of B factors that is currently included (although that should remain).

Sites 1 and 2 are well investigated by mutagenesis, but site 3 is dismissed out of hand "due to the

unlikely binding of Zn3 under physiological conditions". I don't necessarily disagree that this site is an 'adventitious' site - due to the crystals being soaked in relative high concentrations of Zn^{2+} (0.5 – 2 mM) prior to data collection (why a range of concentrations is given is puzzling – the authors should be more specific). However, this statement is unsubstantiated. The simplest approach would be for the authors to mutate those residues (as they did for sites 1 and 2) and then establish whether the mutant protein has altered Zn^{2+} binding properties. If not, then they are well justified in making this statement. This needs to be addressed before acceptance.

I also have some comments on the crystallography that should be addressed:
1. The authors state the data was collected at wavelengths of 0.9 and 1.1 Å – why? The Zn-edge is at 1.2837 Å! The authors need to explain their approach and also annotate the data sets that are referred to in Tables S5 and S6 with the wavelength at which they were collected.
2. The authors have truncated their data for refinement at I/sigI = 5.2. This is presumably because of the statistics (CC1/2 0.582). These poor statistics may be because of the high redundancy of the data and the merging of data between multiple crystals. I understand this was done in order to access SAD phasing (which would have been facilitated by collecting at a more appropriate wavelength!), however a 'native' data set (perhaps from a single crystal) should be used for refinement and this would potentially yield a higher resolution structure.
3. I would have liked to have seen an anomalous difference map from a data set collected low energy of the Zn-edge. This would unequivocally identify all three metal atoms as Zn.
4. The identity of the postulated Cl^- ligands at site 3 need to be better justified. At a wavelength of 1.1 Å, the anomalous scattering coefficients of S and Cl are approximately equal (f" ~0.3 e-). Therefore, given the redundancy of the data, anomalous difference Fourier maps should show peaks at approximately the same sigma level for S (Cys, Met) and Cl^- positions. This needs to be done to justify the current modelling of site 3 (and to unequivocally establish that the ligands are not water).

Reviewer #3 (Remarks to the Author):

Overall I think this is a great work. Dr. Sitia, Dr. Inaba and their colleagues illustrated that intracellular zinc can cause the conformational change of the PDI chaperon ERp44, resulting in the changes of both the subcellular localization and activity of ERp44. This work identified a novel discovery that zinc can regulate protein quality through ERp44 at the ER-Golgi interface. However, several issues should be addressed and clarified as detailed below:

Major issues:

1. Please explain clearly how to measure the zinc binding affinity of ERp44 by ITC analysis. Will the binding affinity depend on the protein concentration? How do you determine the labile zinc concentrations in your buffer ?
2. The results (Supplementary Fig.1A) demonstrated that the zinc binding affinity was reduced at lower pH, which suggests that zinc binding would be different at different pH. However, all the SEC, SEC-MALS and structural work were conducted at pH7.0. Will reducing pH affect zinc binding and dimerization of ERp44 ?
3. The authors clearly demonstrated that changes in cellular zinc concentrations can shift the subcellular localization of ERp44. But the ER localization lacks co-localization marker.
The subcellular distributions of ERp44 look more like vesicular compartments rather than ER (Fig.1E). Please utilize subcellular marker for ER, early endosomes and late endosomes to verify the subcellular localization of ERp44.
4. The authors showed that zinc binding can expose the positive charged residues of the ERp44, inducing increased affinity for the client protein with a negative patch of residues. But how can excess zinc retain ERp44 in ER and how can zinc chelation cause the Golgi translocation for ERp44

?
5. What is the metal selectivity of ERp44? Will ERp44 bind other trace element such as Cu^+?

Minor issues:
1. Abstract, line 3: “bind” should be changed to “binds”.
2. In Fig. 1C, majority of ERp44 with presence of 120 μM on the gel were monomer, not homodimer, which contradicted with what was stated in the text.
3. Please write out the acronym when they were first appeared in the text such as PDI, CNX/CRT, COPII, etc.
4. Zinc treatment is not consistent. The $ZnSO_4$, $ZnCl_2$, zinc/pyrithione have been used in seperate experiments. Can you explain why?
5. Supplementary Figure 1B legend, “in the presence or absence of Zn^{2+} ions (180 μM)” The text stated that 120 μM $ZnCl_2$ were used here instead. Please clarify this.
6. Supplementary Fig 1C: 0.25 mg/ml ERp44 WT were used.
Please change the unit to μM to make sure it is consistent with other results.
7. Page 6. “Wild-type ERp44 localize in ERGIC and cis-Golgi” cited the reference 30 and 36. But these reference did not demonstrate the localization of ERp44. Please correct the references.
8. Please specify the error bars (SEM or SD?) for each graph.
9. Page 9, line 2: 3B should be changed to Figure. 3b.
Line 4: supplementary Fig.3A should be changed to supplementary Fig. 4A.
10. Fig 10C right panel should be labeled with $ZnCl_2$ for clarification.
11. Page 19, line 3: the first “sites” should be changed to “site”.
12. Page 19: “metallothioneins that bind up to eight metal ions”.
Metallothioneins bind up to 7 Zn^{2+} ions not 8.
13. Page 20: Zn-bound should be Zn^{2+}-bound.

Reviewer #4 (Remarks to the Author):

Here, Watanabe et al. Identify a new role of zinc in regulating the ER-Golgi secretory pathway via ERp44 control. The authors showed that Erp44 binds zinc via three bindings site (site 1,2 and 3) that regulate the sub-cellular localization of Erp44. Even though the authors suggest a molecular mechanism that offers a valuable insight into the protein quality control in the ER-Golgi pathway, some major revision should be addressed before publication in Nature Communications.

Major comments:
1. Figure 1 B and Supplementary Figure 1 A. The raw ITC data show a linear increase but the data analysis presents a quite sigmoidal trace. Could the use of NITPIC and SEDPHAT introduce some kind of artifact? Maybe it would be better use Origin software. Are the error bars of the lower panel SD of the two tritations or the error bars from SEDPHAT fitting?
2. The authors determined the number of zinc binding sites using a 1:1 model for the fitting in SEDPHAT, etc. Authors should explain in details the fitting of the ITC data and how the determined the number of binding sites. Moreover, the authors showed that Zn promotes oligomerization of Erp44. How can authors distinguish in the ITC the heat of the binding from the heat of the oligomerization?
3. The same could be applied to the ANS assay. The authors associate the increase in fluorescence to an increase in hydrophobicity due to a conformational change. That could be applied to the case of the experiments at different pHs, but in the presence of Zn, the authors should have into account the oligomerization.
4. Authors affirmed, “The Zn^{2+}-bridged non-covalent homodimer is consistent with the results of the ITC and SEC analyses”. From ITC data the authors can't deduce the oligomerization. Typical ITC oligomerization experiments should be done (injecting high concentration protein to buffer). These experiments will provide with the Kd of dimerization, lacking in the model.

5. Some SEC experiments were conducted at pH 7, they should have repeated at pH 6.2 in order to compare oligomerization in both conditions.
6. The authors affirmed that Zn binding is sequential. Zn-binding ITC data analysis should be re-analyzed using this assumption.
7. Erp44-Ero1a interaction in the presence of Zn was conducted in Zn concentration not saturating for Erp44. Furthermore, authors should have in mind the heat of oligomerization after injecting Erp44 into Ero1a and ZnCl2 cell. Additionally, experiments at pH=7.2 will help to strengthen the proposed model.

Minor comments:
1. Authors should add the standard deviation of the ITC parameter values and some symbols are missing in the scale of supplementary figure 1 A.
2. In fig.2 the size of Ero1α looks higher in OUT. Authors should explain it.
3. In Scatchard plot R(i)= X_{bound}(i) (number of bound ligand)/M_t(i) for multivalent ligand, as in this case.

In summary, the authors presented a model for the regulation of Erp44 by Zn. Taking into account that the model relies heavily on ITC results, I think that authors should address some of the above comments before publishing the manuscript in Nature Communications.

Response to reviewers' comments

Reviewer #1 (Remarks to the Author):

This manuscript describes the role of zinc in the localization and function of ERp44, a PDI family member that patrols the ER-Golgi boundary and retrieves proteins that have not yet completed oxidative folding/assembly and certain proteins that should be localized to the ER. While the pH gradient along the secretory pathway is well known, a zinc gradient is less widely appreciated. The authors convincingly demonstrate that zinc has an important role to play in regulation of the function of ERp44, on top of the role of pH that they had previously demonstrated. Their conclusions are supported by a wide variety of experiments including subcellular localization of ERp44 upon depletion of Golgi zinc by chelators and knockdown of zinc importers, zinc-bound ERp44 crystal structures, size exclusion chromatography, ITC, and secretion assays of client proteins. This comprehensive study, building upon previous insightful work, is summarized in an elegant mechanistic model.

The data are presented exceedingly clearly and aesthetically. The text is elegant and a pleasure to read.

This manuscript makes an important advance to the field of ER quality control and provides important lessons for others studying protein assembly, interactions, and the regulation of protein activity in the ER and Golgi. It should therefore be of broad interest and appeal.

Response: We are grateful to Reviewer 1 for this positive overall evaluation.

Reviewer #2 (Remarks to the Author):

The paper by Watanabe et al., examines the role of Zn2+ binding in the structure and particularly function of the ERp44 protein. I have been asked by the editor to comment on the structure biology aspects of the paper. What follows below concentrates on that aspect, but also the Zn2+ binding analyses, given that is an additional area of my expertise. Overall, in assessing those aspects, I believe the paper will be suitable for

publication in Nature Communications once the following issues are addressed:

Response:

We are grateful to the Reviewer 2 for this positive overall evaluation.

Comment 1

The study found, by ITC that Zn2+ bound the protein at a molar ratio of 1.5:1 (Zn2+:protein) and that metal binding also promoted its dimerization (non-covalent). I am convinced by this analysis. However, I feel the cooperativity between binding sites 1 and 2 is less convincing and should be revisited before the manuscript is suitable for publication. The authors should consider that ITC is not perhaps the ideal technique for distinguishing two binding sites with potentially different binding constants and coorperativity between them. A competition assay with a fluorescent Zn2+ complex or other spectrophotometrically-active ligand with a binding constant in the appropriate range may be able to distinguish the two binding events: the first to site 1, followed by site 2 and dimerization. FluoZin-3 is one such ligand, but a simple survey of the literature will reveal others. I think at the moment the coorperativity of binding is not underpinned appropriately by the experimental data presented.

Response: According to this suggestion, we performed a competition assay using the fluorimetric dye Mag-fura-2 (MF-2) (see attached figure 1). This dye is reported to have a Zn^{2+}-binding affinity of K_d = 30~85 nM, nearly comparable to ERp44. The absorption spectral change during the titration of MF-2 with Zn^{2+} were measured and analyzed with the program Dynafit (attached figure 1A & 1D blue). In our hands, the K_d for Zn^{2+} binding to MF-2 was determined to be 89 ± 27 nM, similar to the literature value (Seny et al, JBC, 2001). As expected, the co-presence of 5 μM ERp44 significantly prevented the Zn^{2+}-dependent decrease of absorbance at 362 nm and displayed a titration curve with multiple phases (attached figure 1B), suggesting that ERp44 competes with MF-2 for binding Zn^{2+}. However, the absorbance change did not reach the plateau until a total Zn^{2+} concentration of >25 μM, much over the calculated saturation point (8.3 μM MF2+1.5 x 5.3 μM ERp44= 16.3 μM). One possible interpretation is that ERp44 itself interferes with MF-2 in Zn^{2+} binding, resulting in the apparently lower Zn^{2+} binding affinity of MF-2. To test this possibility, we performed the same assay using ERp44 3HA mutant that is unable to bind Zn^{2+}. ERp44 3HA also prevented the Zn^{2+}-dependent

decrease of absorbance at 362 nm, though to a lesser extent than ERp44 WT (attached figure 1C & 1D). Thus, ERp44 itself appears to interfere with MF-2 in binding Zn^{2+} possibly through direct interactions with this dye. In the presence of ERp44 3HA, the apparent K_d for Zn^{2+} binding to MF-2 (K_{dL}) was estimated to be 2.1 ± 0.16 μM (attached figure 1D red). Using this value, the observed titration curve was fitted by a model represented by the following Equations 1-3 (attached figure 1D green). Eventually, K_1 and K_2 were estimated to be 0.58 ± 0.32 μM and 0.46 ± 0.46 μM^2, respectively.

Eq. 1

$$\text{MF-2} + \text{Zn} \Longleftrightarrow \text{MF-2-Zn} \qquad K_{dL} = \frac{[\text{MF-2}]\,[\text{Zn}]}{[\text{MF-2-Zn}]}$$

Eq. 2

$$\text{E} + \text{Zn} \Longleftrightarrow \text{E-Zn} \qquad K_1 = \frac{[\text{E}]\,[\text{Zn}]}{[\text{E-Zn}]}$$

Eq. 3

$$\text{Zn} + 2\,\text{E-Zn} \Longleftrightarrow \text{E}_2\text{Zn}_3 \qquad K_2 = \frac{[\text{Zn}]\,[\text{E-Zn}]^2}{[\text{E}_2\text{Zn}_3]}$$

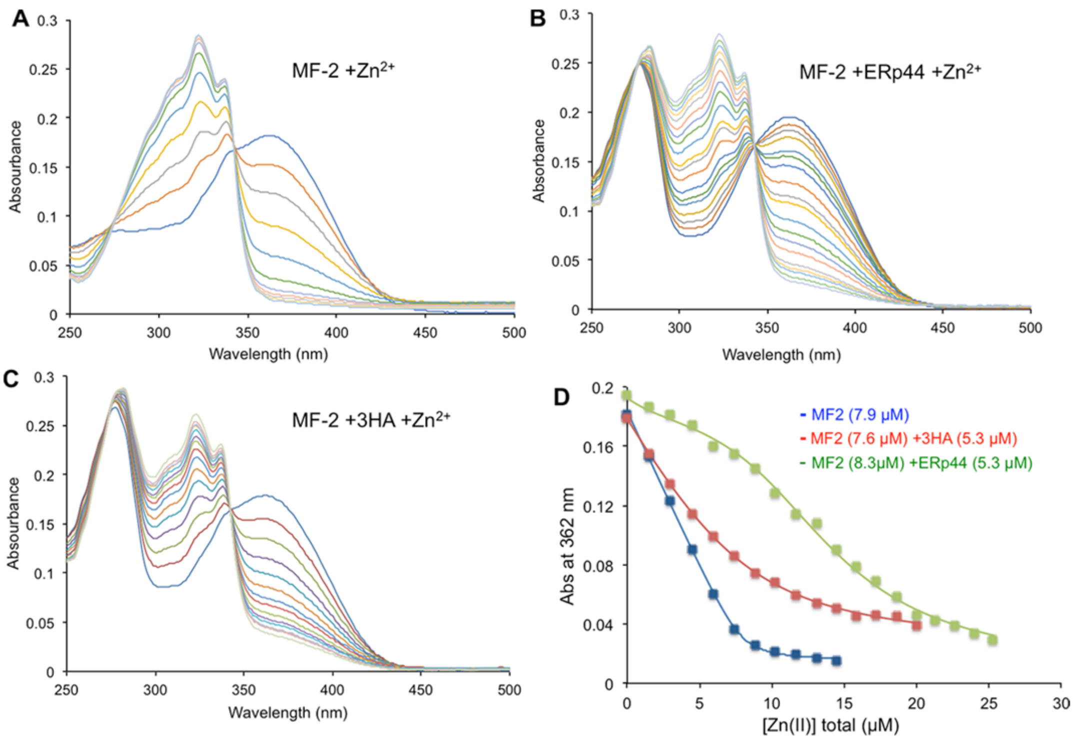

Attached Figure 1 Competition titration analysis for Zn^{2+} binding to MF-2 in presence of ERp44 (A-C) Absorbance spectral changes during the titration of (A) MF-2 (8μM), (B) MF-2 (8 μM) and ERp44 (5.3 μM), (C) MF-2 (8μM) and 3HA mutant (5.3 μM) with $ZnCl_2$, in 20 mM HEPES pH7.0, 150 mM NaCl. The spectra were recorded with Hitachi U-3900 spectrophotometer at room temperature (D) Absorbance at 362 nm is plotted as a function of

total Zn^{2+} concentration and fitted with Dynafit. The fitting curves are represented in blue (MF-2 only; K_{dL}= 0.089 μM), red (MF-2 + 3HA; K_{dL}' = 2.1 μM), and green (MF-2 + ERp44; K_1 = 0.58 μM, K_2 = 0.46 μM^2 with fixed K_{dL} = 2.1μM) lines, respectively.

The estimated K_1 and K_2 values provide important information on Zn^{2+} binding affinity presumably at site 1 and site 2, respectively. However, it seems hard to conclude the cooperativity in sequential Zn^{2+} binding to ERp44 based on this result, because K_2 (μM^2), an equilibrium constant for one Zn^{2+} binding to two Zn^{2+}-bound ERp44 molecules, has a different dimension from K_1 (μM), that for one Zn^{2+} binding to one metal-free ERp44 molecule. In addition, K_d for Zn^{2+}-binding of MF-2 in the presence of ERp44 WT or Zn^{2+}-bound ERp44 may not necessarily be the same as that in the presence of 3HA mutant. Given such situation, we decided not to include these results in the revised manuscript.

Instead, to gain further insights into the cooperative Zn^{2+} binding to ERp44, we reanalyzed the Scatchard plots based on the observed ITC data by considering the heat of dilution ($q_{ditluion}$) in calculation of the $X_{\text{bound}}(i)$ values as below.

$$X_{\text{bound}}(i) = [Q(i)/(\Delta H V_0)] + X_{\text{bound}}(i\text{-}1)$$

Herein, we replaced ΔH *to* $(\Delta H + q_{ditluion})$ and thereby recalculated Scatchard plots. The resultant plots represent more typical concave downward curves than the original ones (new Supplementary Figure 8A). Moreover, we calculated saturation plots based on the ITC data to estimate the Hill coefficients. We thus found that the Hill coefficients were significantly higher than 1 (new Supplementary Figure 8B), further supporting the positive cooperativity in sequential Zn^{2+} binding to ERp44. Multiple ligand binding with positive cooperativity frequently displays an apparently single-phase ITC curve (Tochtrop et al., ***PNAS*** 99, 1847-1852, 2002; Sequential binging sites model in Origin user manual, etc), as observed with Zn^{2+}-binding to ERp44 (Fig. 1B). In line with this, significant amounts of Zn^{2+}-mediated ERp44 dimers were generated even at ERp44/Zn^{2+} ratios of 1:0.5 to 1:1, that is, before the saturation of site 1 with Zn^{2+} (new Supplementary Figure 8C). Taken all together, we conclude that the positive cooperativity exists in Zn^{2+}-binding to sites 1 and 2.

Comment 2

The crystal structure observed three Zn2+ sites per protein – site 1 (3 x His + water),

site 2 (at the interface; 4 x His) and site 3 (His, Cys, Tyr + 2xCl-). The authors describe the binding geometries as tetrahedral. I would like to see coordination distances and angles included in the Supplementary Information for all three sites. This would give confidence in the quality in the refinement - that chemically-sensible sites have been modeled (His - Zn2+ coordination distances for example should be ~2.0-2.1 Å). This would be far more convincing than the Table of B factors that is currently included (although that should remain).

Response: According to this suggestion, we added Table showing the coordination distances and angles at the three Zn^{2+}-binding sites (Supplementary Table 8) in the revised manuscript.

Comment 3

Sites 1 and 2 are well investigated by mutagenesis, but site 3 is dismissed out of hand "due to the unlikely binding of Zn3 under physiological conditions". I don't necessarily disagree that this site is an 'adventitious' site - due to the crystals being soaked in relative high concentrations of Zn2+ (0.5 – 2 mM) prior to data collection (why a range of concentrations is given is puzzling – the authors should be more specific). However, this statement is unsubstantiated. The simplest approach would be for the authors to mutate those residues (as they did for sites 1 and 2) and then establish whether the mutant protein has altered Zn2+ binding properties. If not, then they are well justified in making this statement. This needs to be addressed before acceptance.

Response: According to this suggestion, we constructed a His333/Ala mutant (H333A) and examined its Zn^{2+}-binding properties (new supplementary Fig. 9). SEC analysis showed that H333A was eluted only slightly faster in the presence of $ZnCl_2$ (new supplementary Fig. 9A), suggesting that this mutant did not form a stable homodimer with Zn^{2+}. Consistent with this, His333 forms a π-π stacking interaction with Phe31 at the interface of ERp44 homodimer (Fig. 3B, right panel), contributing to stabilization of the dimer conformation.

Of note, ITC analysis showed that H333A mutant bound one Zn^{2+} per monomer, with much lower affinity (K_d =2.49 μM) than WT and H277/281/A (2HA) mutant (new supplementary Fig. 9B). This may be explained by the likely perturbation of H333A mutation on the local conformation at site 1 involving His328 and His332.

Thus, His333 seems to have auxiliary roles in Zn^{2+} binding to ERp44 and hence dimer formation. These additional insights have been incorporated in the revised manuscript (page 13, lines 16- page 14, line 4). These results are consistent with our previous *in vivo* observation that H333A mutation allowed some ERp44 secretion (Sannino et al., ***J. Cell Sci.*** 2014).

As for Zn^{2+} concentration in crystallization, crystals of Zn^{2+}-bound form of ERp44 were indeed obtained at a range of 0.5 mM to 2 mM $ZnCl_2$.

Comment 4-1

I also have some comments on the crystallography that should be addressed:
The authors state the data was collected at wavelengths of 0.9 and 1.1 Å – why? The Zn-edge is at 1.2837 Å! The authors need to explain their approach and also annotate the data sets that are referred to in Tables S5 and S6 with the wavelength at which they were collected.

Response: According to this suggestion, we added wavelength information in Supplementary Tables 6. Also, we added supplemental explanations regarding the process of the structure determination in Methods section of the revised manuscript (page 42-43).

Previously, we reported higher-resolution crystal structure of metal-free ERp44 using the mutant that lacks the C-terminal RDEL motif (ERp44 ΔRDEL) (Watanabe, et al., ***PNAS*** 2017). Thus, we initially employed ERp44 ΔRDEL (residues 1-373) for co-crystallization with Zn^{2+} and determined its structure by the SAD method using anomalous signals of Zn^{2+}. The X-ray diffraction data were collected at the Zn^{2+} peak wavelength (1.258Å) on beamline BL44XU at SPring-8 (Attached Table 1), allowing us to observe ten strong anomalous peaks (>15σ) in the crystallographic asymmetric unit containing two ERp44 dimers (attached Figure 2A and 2C).

We thus found that ERp44 ΔRDEL forms a Zn^{2+}-mediated homodimer with five Zn^{2+} binding sites at the interface like ERp44 WT (attached Figure 2C). The coordination patterns of site 2 and site 3 in ΔRDEL are almost identical to those in WT. Site 1 in Mol A of ΔRDEL is constituted by the three histidine residues (His-cluster) like that of WT. However, at site 1 in Mol B, Glu residue (Glu365) was found to coordinate Zn^{2+} instead of a water molecule (attached Figure 2C). We also found that

deletion of the RDEL motif significantly altered local conformation of the C-tail at the dimer interface (attached Figure S3A). In this connection, the Zn^{2+} binding affinity of ERp44 ΔRDEL was significantly compromised (attached Figure S3B), Thus, the results of ΔRDEL contain some artificial aspects and might rather confuse readers. We therefore decided not to incorporate the structural and biophysical data of ERp44 ΔRDEL in this paper. However, we are ready to insert them if requested by the editor and/or Reviewers.

As for Zn^{2+}-bound ERp44 WT, we initially determined its structure by molecular replacement using the pre-determined crystal structure of Zn^{2+}-bound ERp44 ΔRDEL. Although its overall structure was clearly resolved, the electron density map of the C-tail region was missing or too poor to build up the model. We collected >10 "native dataset", but could not obtain a single dataset of high enough resolution to visualize the C-tail region clearly. However, we found that merging these "native dataset" allowed the SAD phasing and improved resolution and statistics. In addition, merging the dataset of high CC values improved local electron density map of the C-tail and allowed its model building. In order to remove the model bias, we finally determined the crystal structure of Zn^{2+}-bound ERp44 by Zn^{2+}-SAD using the merged native dataset with high CC values (Supplementary Table S6). Although our approach is not a conventional method, it is theoretically possible and feasible. We added supplemental explanations regarding our process of the structure determination in Methods section of the revised manuscript (page 42-43).

Attached Table I: Data collection and refinement statistics of crystal structure of Zn2+-bound ERp44 ΔRDEL

	Zn2+-bound ERp44 ΔRDEL	
**Data collection**	**peak**	**remote**
Wavelength (Å)	1.2815	1.3000
Space group	$P6_122$	$P6_122$
Cell dimensions		
$a = b$ (Å)	175.30	175.15
c (Å)	407.19	407.81
Resolution (Å)[a]	49– 2.50 (2.56-2.50)	48.5– 3.00 (3.1-3.00)
Refs. total/unique	2719250 / 127535	1658293 / 75435
Completeness (%)[a]	99.8 (97.9)	100.0 (99.8)
Redundancy	21.3	22.0
$I/\sigma(I)$[a]	21.5 (1.5)	19.2 (2.2)
R_{meas} (%)	11.8 (170)	31.2 (194)
$CC_{1/2}$	0.999 (0.56)	1.00 (0.75)
**Phasing**		
CC_{all}/CC_{weak} (%)	48.62/30.39	
FOM after SHELXE	0.67	
CC for auto-tracing in SHELXE (%)	34.5	
**Refinement**		
R_{work}/R_{free} (%)	20.2/23.1	
R.m.s. deviations		
bond length (Å)	0.01	
bond angles (˚)	0.98	
Ramachandran plot		
favored (%)	96.7	
allowed (%)	3.3	
outliers (%)	0.0	

[a]Values in parentheses are for the high-resolution shell.

[b]$R_{meas} = \Sigma_{hkl} \{n/[n-1]\}^{1/2} \Sigma_i|I_i(hkl) - \langle I(hkl)\rangle|/\Sigma_{hkl}\Sigma I_i(hkl)$, where $I_i(hkl)$ is the *i*th observed intensity and $\langle I(hkl)\rangle$ is the average intensity from multiple observations

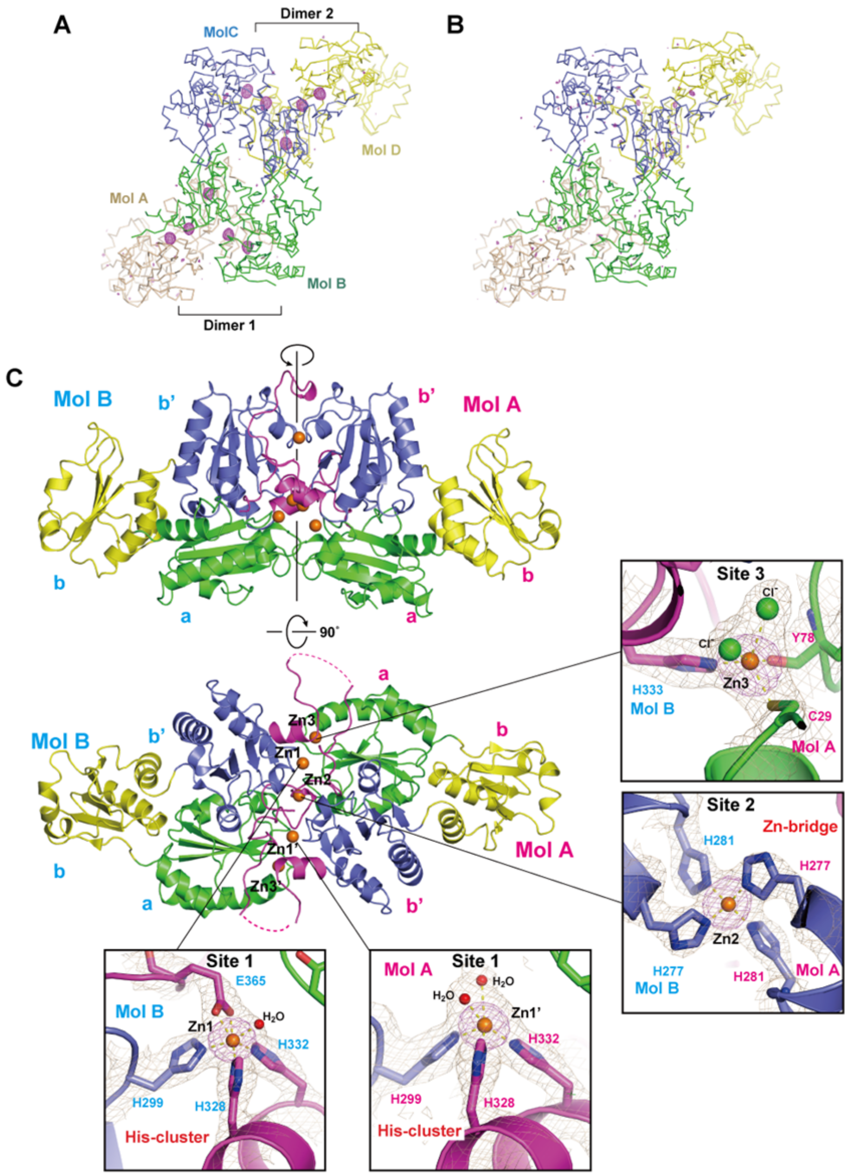

Attached Figure 2 Crystal structure of Zn^{2+} bound form of ERp44 ΔRDEL. (**A**, **B**) Anomalous difference Fourier map at 3.5σ in the crystallographic asymmetric units are shown in magenta. The maps are calculated using (A) the Zn^{2+} peak dataset collected at 1.2815Å wavelength or (B) the remote dataset collected at 1.300Å wavelength, respectively. (C) Top and side view of the overall structure of the Zn^{2+}-bound dimer of the ΔRDEL mutant. The right and lower insets display the close-up views of the three Zn^{2+} binding sites: site 1 (lower panels), site 2 (middle) and site 3 (top). The SAD-phased 2Fo-Fc maps at 1.5~2 σ and anomalous difference Fourier map at 15 σ are shown in brown and magenta, respectively.

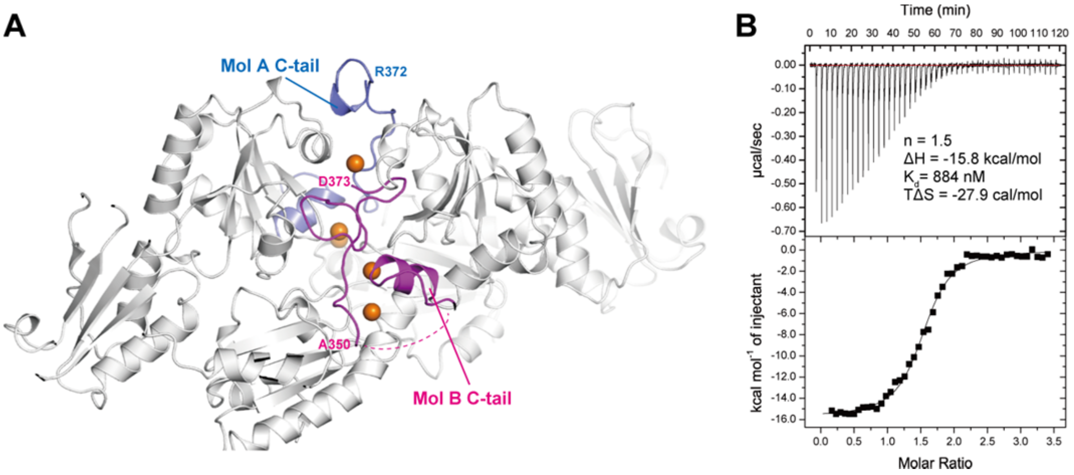

Attached Figure 3 Deletion of the RDEL motif alters the C-tail conformation and Zn^{2+} binding ability of ERp44.

(**A**) Highlighted view of the C-tail region (residues 326-373) of the two protomers in the Zn-bound ΔRDEL dimer. The C-tail regions are coloured in blue (mol A) and magenta (Mol B) respectively (**B**) ITC raw data (upper) and binding isotherm data (lower) for titration of $ZnCl_2$ into the ΔRDEL mutant (30 μM) at pH 7.0.

Comment 4-2: The authors have truncated their data for refinement at I/sigI = 5.2. This is presumably because of the statistics (CC1/2 0.582). These poor statistics may be because of the high redundancy of the data and the merging of data between multiple crystals. I understand this was done in order to access SAD phasing (which would have been facilitated by collecting at a more appropriate wavelength!), however a 'native' data set (perhaps from a single crystal) should be used for refinement and this would potentially yield a higher resolution structure.

Response: We agreed with the reviewer. The suggested approach is a conventional way of structure determination. Intriguingly, however, the merged data displayed better statistics than each native dataset (Please see supplementary Tables 5 and 6). For instance, each native dataset has worse CC1/2 value and lower I/sig at 2.45Å resolution than the merged data. As mentioned in our response to the previous comment, the merged data significantly improved electron density map, in particular for the C-tail region. Thus, we used the merged dataset not only for the SAD phasing but also for the final structure refinement at 2.45Å resolution. We added the supplemental explanations in Methods section (page 42, middle).

Comment 4-3.

I would have liked to have seen an anomalous difference map from a data set collected low energy of the Zn-edge. This would unequivocally identify all three metal atoms as Zn.

Response: According to this suggestion, we calculated anomalous difference Fourier map from a dataset collected at low energy of the Zn-edge (wavelength: 1.300Å) using crystals of the Zn^{2+}-bound ERp44 ΔRDEL (Attached Table I). As expected, ten strong anomalous peaks (>15σ) observed in the Zn peak dataset (attached Figure 2A) were significantly diminished in the remote dataset collected at 1.300Å (attached Figure 2B), supporting that the bound metal atoms are Zn.

Comment 4-4

The identity of the postulated Cl- ligands at site 3 need to be better justified. At a wavelength of 1.1 Å, the anomalous scattering coefficients of S and Cl are approximately equal (f' ~0.3 e-). Therefore, given the redundancy of the data, anomalous difference Fourier maps should show peaks at approximately the same sigma level for S (Cys, Met) and Cl- positions. This needs to be done to justify the current modelling of site 3 (and to unequivocally establish that the ligands are not water).

Response: Thank you for this useful advice. As suggested, some anomalous peaks of S atoms are actually observed in the anomalous difference Fourier map. Unfortunately, the anomalous scattering peaks of the putatively placed Cl^- overlapped with even stronger peaks of Zn3, and hence we could not precisely quantify the sigma level of the Cl^- ion from the anomalous difference Fourier map.

However, when water molecules were tentatively placed at these positions, positive peaks were still observed to significant level in the Fo-Fc map and the water molecules had unusually low B-factors. In contrast, placing Cl^- ions at these positions resulted in no extra electron density in the Fc-Fc map and gave reasonable B-factors for the ions. Altogether, we believe that Cl^- ions, rather than water molecules, coordinates the Zn3. This supplementary explanation has been added in the revised manuscript (page 43, lines 21- page 44 line 2).

Reviewer #3 (Remarks to the Author):

Overall I think this is a great work. Dr. Sitia, Dr. Inaba and their colleagues illustrated that intracellular zinc can cause the conformational change of the PDI chaperon ERp44, resulting in the changes of both the subcellular localization and activity of ERp44. This work identified a novel discovery that zinc can regulate protein quality through ERp44 at the ER-Golgi interface. However, several issues should be addressed and clarified as detailed below:

Response: We are grateful to the Reviewer 3 for this positive overall evaluation.

Comment 1

Please explain clearly how to measure the zinc binding affinity of ERp44 by ITC analysis. Will the binding affinity depend on the protein concentration? How do you determine the labile zinc concentrations in your buffer?

Response: The apparent Zn^{2+} binding affinity was estimated by fitting the ITC data with a 1:1 binding model using programs SEDPHAT and Origin, where fitting parameters are apparent binding affinity (K_a), enthalpy change (ΔH) and stoichiometry (n) (please see also our responses to Reviewer 4). We verified that the Zn^{2+} binding affinity of ERp44 did not largely change in the protein concentration range of 10-60μM (see attached Fig. 4). Since the ITC binding isotherm data for titration of $ZnCl_2$ (500 μM) into 30 μM of ERp44 is the best in terms of the S/N ratio and the C-value (a ratio of protein concentration to K_d for ligand), we only showed the data measured under this condition in the revised manuscript.

Considering that Zn^{2+} has a property to precipitate as $Zn(OH)_2$ at weakly alkaline pH, Zn^{2+} solution used for the ITC measurement was prepared by diluting a stock solution of 2M $ZnCl_2$ in ~20 mM HCl with indicated buffer. The stock solution and titrant solution were prepared just before measurements. We added this information in the Methods section of the revised manuscript (page 38 line 18-21).

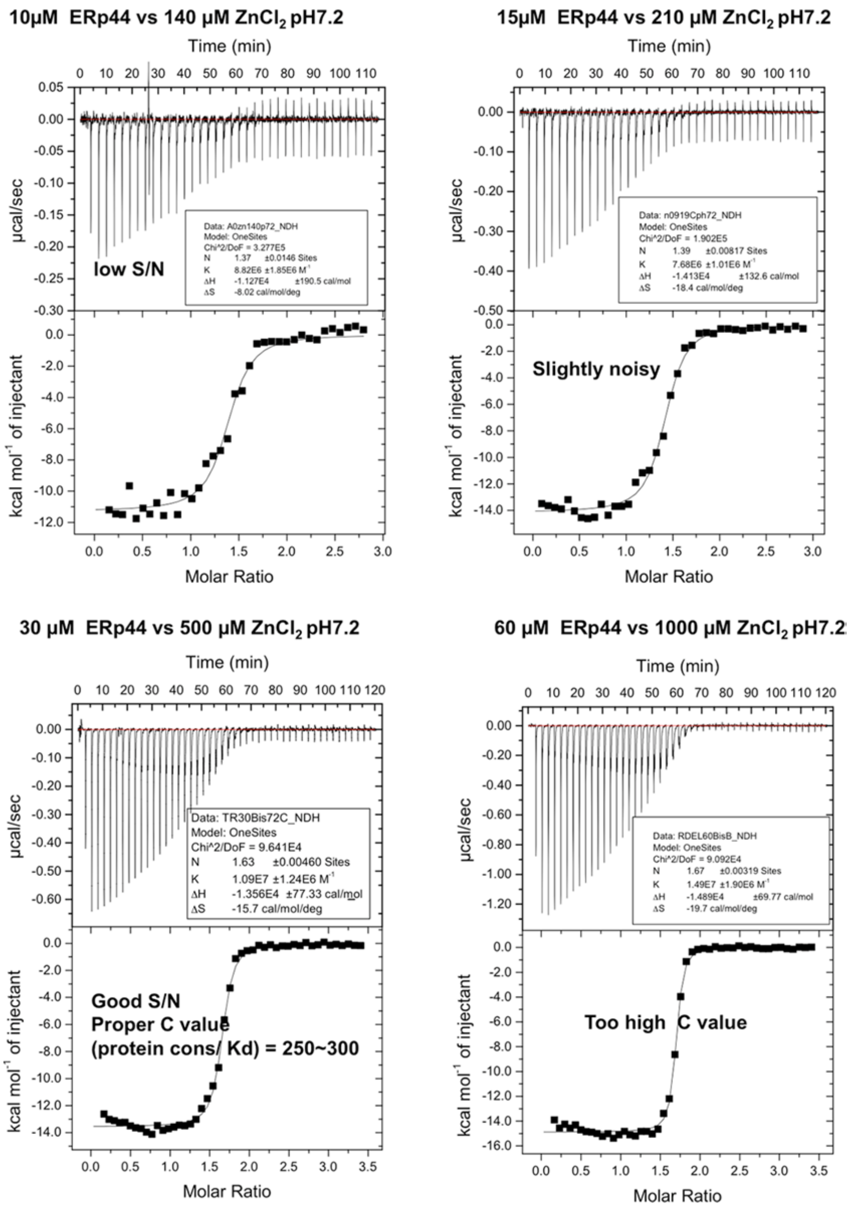

Attached Figure 4 ITC analysis for Zn^{2+} binding to different concentrations of ERp44

ITC raw data (upper) and binding isotherm data (lower) for titration of $ZnCl_2$ into 10, 15, 30 and 60 μM of ERp44 at pH 7.2. An equilibrium constant for Zn^{2+} binding to ERp44 is calculated to be (8.82±1.85) x 10^6 M^{-1} for 10 μM ERp44 vs 140 μM $ZnCl_2$, (7.68±1.01) x 10^6 M^{-1} for 15 μM ERp44 vs 210 μM $ZnCl_2$), (10.9±1.24) x 10^6 M^{-1} for 30 μM ERp44 vs 500 μM $ZnCl_2$, and (14.9±1.90) x 10^6 M^{-1} for 60 μM ERp44 vs 1000 μM $ZnCl_2$.

Comment 2

The results (Supplementary Fig.1A) demonstrated that the zinc binding affinity was reduced at lower pH, which suggests that zinc binding would be different at different pH. However, all the SEC, SEC-MALS and structural work were conducted at pH7.0. Will reducing pH affect zinc binding and dimerization of ERp44?

Response: We performed the SEC analyses for detection of the Zn^{2+}-induced ERp44 dimerization at pH 6.2 (Figure 4C, blue dotted line) and pH 6.7 (Supplementary Figure 8C). This information was not clearly described in the original manuscript, and therefore we added the information in the legends to those figures. Furthermore, to address this comment appropriately, we also performed additional SEC experiments at pH 7.2, 6.7 and 6.2, as shown in new Supplementary Figure 1B. The results clearly demonstrate that ERp44 forms a Zn^{2+}-mediated homodimer in a pH range of 6.2 to 7.2. This statement is added to the revised text (page 5, lines 15-16).

The Zn^{2+}-binding sites of ERp44 consist of three (site 1) and four (site 2) histidine residues.–In general, the protonation states of histidines depend on pH and control the metal binding affinity. Thus, reduced Zn^{2+}-binding affinity of ERp44 at lower pH (Supplementary Figure 1A) can be explained by higher-degree protonation of the histidines at lower pH. We added this explanation in the revised manuscript (page 20, lines 22)

Comment 3

The authors clearly demonstrated that changes in cellular zinc concentrations can shift the subcellular localization of ERp44. But the ER localization lacks co-localization marker. The subcellular distributions of ERp44 look more like vesicular compartments rather than ER (Fig.1E). Please utilize subcellular marker for ER, early endosomes and late endosomes to verify the subcellular localization of ERp44.

Response: We previously reported that a major fraction of ERp44 co-localizes with ERGIC53 in the vesicular-like compartments (Anelli et al., (2007) ***EMBO J***). According to this comment, we immunostained for ERp44, Calnexin (ER marker), GM130 (cis-Golgi marker), ERGIC53 (ERGIC marker), EEA1 (early endosome marker), and Rab7 (late endosome marker), and thereby verified that ERp44 co-distributes nicely with ERGIC53 and to a lesser extent with Calnexin and GM130.

No co-localization was observed with EEA1 or Rab7, excluding the possibility that ERp44 is localized in the early or late endosomes. We inserted this data in new Supplementary Figure 2 and added this information in the revised manuscript (page 6, lines 16-17).

Comment 4

The authors showed that zinc binding can expose the positive charged residues of the ERp44, inducing increased affinity for the client protein with a negative patch of residues. But how can excess zinc retain ERp44 in ER and how can zinc chelation cause the Golgi translocation for ERp44 ?

Response: Our crystal structures of Zn^{2+}-bound and -unbound forms of ERp44 revealed that Zn^{2+} binding induces striking conformational change of the C-tail as well as the exposure of the positively charged residues. Consequently, the C-terminal RDEL sequence is fully exposed to the solvent and hence could be readily recognized by KDEL Receptors (KDELR) at the Golgi. ERp44 is then transported to the ER with high efficiency. Thus, excess Zn^{2+} likely promotes the faster retrieval of ERp44, leading to its predominant localization in the ER. By contrast, Zn^{2+} chelation prevents the solvent exposure of the RDEL sequence by keeping the C-tail closed. ERp44 in this conformation will hardly be recognized by KDELR. Thus, Zn^{2+}-depletion likely prevents the association of ERp44 with KDELR, resulting in its accumulation in the Golgi. This supplementary explanation has been inserted to the revised manuscript (page 20, lines 7-13).

Comment 5.

What is the metal selectivity of ERp44? Will ERp44 bind other trace element such as Cu+?

Response: According to this suggestion, we examined Cu^{2+} and Mn^{2+} binding ability of ERp44 by ITC analysis (Supplementary Fig. 5A, B). Indeed, Cu transporters and Mn transporters are known to be localized in the secretory pathway (Polishchuk & Lutsenko ***Histochem Cell Biol***. 2013, He et al., ***Neurochem Res.*** 2012). The ITC analysis showed

that ERp44 binds Cu^{2+} ions, but with micromolar affinity (K_d =1.65 μM) (Supplementary Fig. 5A). Binding analysis of Cu^{+} was technically difficult due to its extremely high tendency to be oxidized to Cu^{2+}. Notably, copper chelation by tetraethylenepentamine affected only marginally the subcellular localization of ERp44 (Supplementary Fig. 5C, D, E), suggesting that copper is not involved in ERp44 regulation. No interactions were detected between ERp44 and Mn^{2+} (Supplementary Fig. 5B). These results corroborate that ERp44 is regulated specifically by Zn^{2+} under physiological conditions. We added this information in the revised text (page 8, bottom to page 9, top).

Comment 6-1.

Minor issues:

Abstract, line 3: “bind” should be changed to “binds”.

Response: Corrected.

Comment 6-2.

In Fig. 1C, majority of ERp44 with presence of 120 μM on the gel were monomer, not homodimer, which contradicted with what was stated in the text.

Response: SEC analysis in combination with non-reducing SDS-PAGE clearly demonstrates that ERp44 forms “noncovalent” homodimers (not disulfide-bridged homodimer) in the presence of 120 μM $ZnCl_2$. For clarity, the supplementary phrase has been added in the legend to Fig. 1C.

Comment 6-3.

Please write out the acronym when they were first appeared in the text such as PDI, CNX/CRT, COPII, etc.

Response:

According to this suggestion, we spelled out these acronyms in the revised manuscript (page 3, line 15; page 3, line 17).

Comment 6-4.

Zinc treatment is not consistent. The ZnSO4, ZnCl2, zinc/pyrithione have been used in separate experiments. Can you explain why?

Response: Since chloride ions are known to precipitate Fe^{3+}, an essential metal ion, at neutral pH, we ceased to add $ZnCl_2$ to cultured cells. In this context, we reexamined the effect of $ZnSO_4$ on the subcellular localization of ERp44 in HepG2 cells and replaced the original imaging data with the new one in new Supplementary Figure 3A. The usage of $ZnCl_2$ is much less problematic for *in vitro* experiments using purified proteins and buffers with limited metal ions such as Na^+. We used zinc/pyrithione to increase intracellular Zn^{2+} concentration in a short time as this compound could bypass the need for ZnT transporters. In live-cell imaging analyses, the observation time should be limited in order to minimize cellular damages by laser and other factors. This supplemental information has been inserted into the revised manuscript (page 44. Lines 19-20 and page 46, lines 19-20).

Comment 6-5

Supplementary Figure 1B legend, "in the presence or absence of Zn2+ ions (180 μM)" The text stated that 120 μM ZnCl2 were used here instead. Please clarify this.

Response: As mentioned in the Methods section, whereas SEC analyses were conducted at 277K in presence of 120 μM $ZnCl_2$, SEC-MALS was performed at room temperature with 180μM $ZnCl_2$. We thus found that ERp44 was eluted as a broader peak at room temperature, suggesting that Zn^{2+}-bridged ERp44 dimers partially disassemble under this condition. We could not conduct the SEC-MALS experiment at 277K because of instrument limitation. For the purpose of preventing the partial disassembly of the ERp44 dimers, we performed SEC-MALS analysis with higher concentration (180μM) of $ZnCl_2$. We added this supplementary explanation in the legend to new Supplementary Fig. 1C.

Comment 6-6.

Supplementary Fig 1C: 0.25 mg/ml ERp44 WT were used.

Please change the unit to μM to make sure it is consistent with other results.

Response: As requested, we changed the unit in the legend to new Supplementary Fig. 1D.

Comment 6-7.
Page 6. “Wild-type ERp44 localize in ERGIC and cis-Golgi” cited the reference 30 and 36. But these reference did not demonstrate the localization of ERp44. Please correct the references.

Response: We thank the referee for finding this imprecision, which likely originated during text editing with different computers. We corrected the citations (page 6, lines 10 and 16)

Comment 6-8.
8. Please specify the error bars (SEM or SD?) for each graph.

Response: We specified the error bars in Fig. 2B and 2D and modified their legends accordingly (Fig. 2B and 2D).

Comment 6-9.
Page 9, line 2: 3B should be changed to Figure. 3b.
Line 4: supplementary Fig.3A should be changed to supplementary Fig. 4A.

Response: Corrected (page 9, lines 14 and 16). Original supplementary Fig. 4A has been moved to supplementary Fig. 6A in the revised manuscript.

Comment 6-10.
Fig 10C right panel should be labeled with $ZnCl_2$ for clarification.

Response: Corrected.

Comment 6-11.
Page 19, line 3: the first “sites” should be changed to “site”.

Response: Corrected (page 20, line 20).

Comment 6-12.

Page 19: "metallothioneins that bind up to eight metal ions".

Metallothioneins bind up to 7 Zn2+ ions not 8.

Response: Corrected (page 21, line 16).

Comment 6-13.

13. Page 20: Zn-bound should be Zn2+-bound.

Response: Corrected (page 22, line 3).

Reviewer #4 (Remarks to the Author):

Here, Watanabe et al. Identify a new role of zinc in regulating the ER-Golgi secretory pathway via ERp44 control. The authors showed that Erp44 binds zinc via three bindings site (site 1,2 and 3) that regulate the sub-cellular localization of Erp44. Even though the authors suggest a molecular mechanism that offers a valuable insight into the protein quality control in the ER-Golgi pathway, some major revision should be addressed before publication in Nature Communications.

Response

We are grateful to Reviewer 4 for this positive overall evaluation.

Comment 1.

Major comments:

Figure 1 B and Supplementary Figure 1 A. The raw ITC data show a linear increase but the data analysis presents a quite sigmoidal trace. Could the use of NITPIC and SEDPHAT introduce some kind of artifact? Maybe it would be better use Origin software. Are the error bars of the lower panel SD of the two titrations or the error bars from SEDPHAT fitting?

Response: We thank the reviewer for the careful review of our ITC data. As suggested by this reviewer, we analyzed the ITC data using Origin, and found that the results were

similar to those obtained using SEDPHAT (see attached Figure 5A and supplementary Fig. 1A). It is therefore unlikely that the sigmoidal traces shown in the binding isotherm data (lower panels in Fig. 1B and supplementary Fig. 1A) are ascribed to artifact caused by the usage of NITPIC/SEDPHAT. As pointed out by this reviewer, the peak height in the ITC raw data showed an apparently linear decrease, whereas the integrated heat changes at each injection in the isotherm data did not decrease in the early stage of titration, yielding the quite sigmoidal curve. A close-up view of the ITC peaks (attached Figure 5B) revealed that the peaks became unusually broader as the titration proceeded during the first ~20 injections, leading to marginal changes in the integrated peak area in this titration range. The observed ITC raw data showing an apparent single-phase isotherm curve is characteristic of ligand binding with positive cooperativity (Tochtrop et al., ***PNAS*** 99, 1847-1852, 2002). The peak shape changes observed during the titration (attached Figure 5B) may reflect that the ratio of the Zn1 (Zn^{2+} at site 1) binding to the subsequent Zn2 (Zn^{2+} at site 2)-mediated dimerization decreases as the injection increases.

In the lower panels of Fig. 1B and Supplementary Fig. 1A, bars represent the errors in the peak integration for each titration estimated by NITPIC. In the global analysis by SEDPHAT, the program deals with these errors considering a weight for individual data points. In contrast, Origin does not estimate integration errors, and treats all data points equally. We thus decided to display the thermodynamic parameters K_a and ΔH determined by NITPIC and SEDPHAT in the manuscript. This information has been added in the legends to these figures.

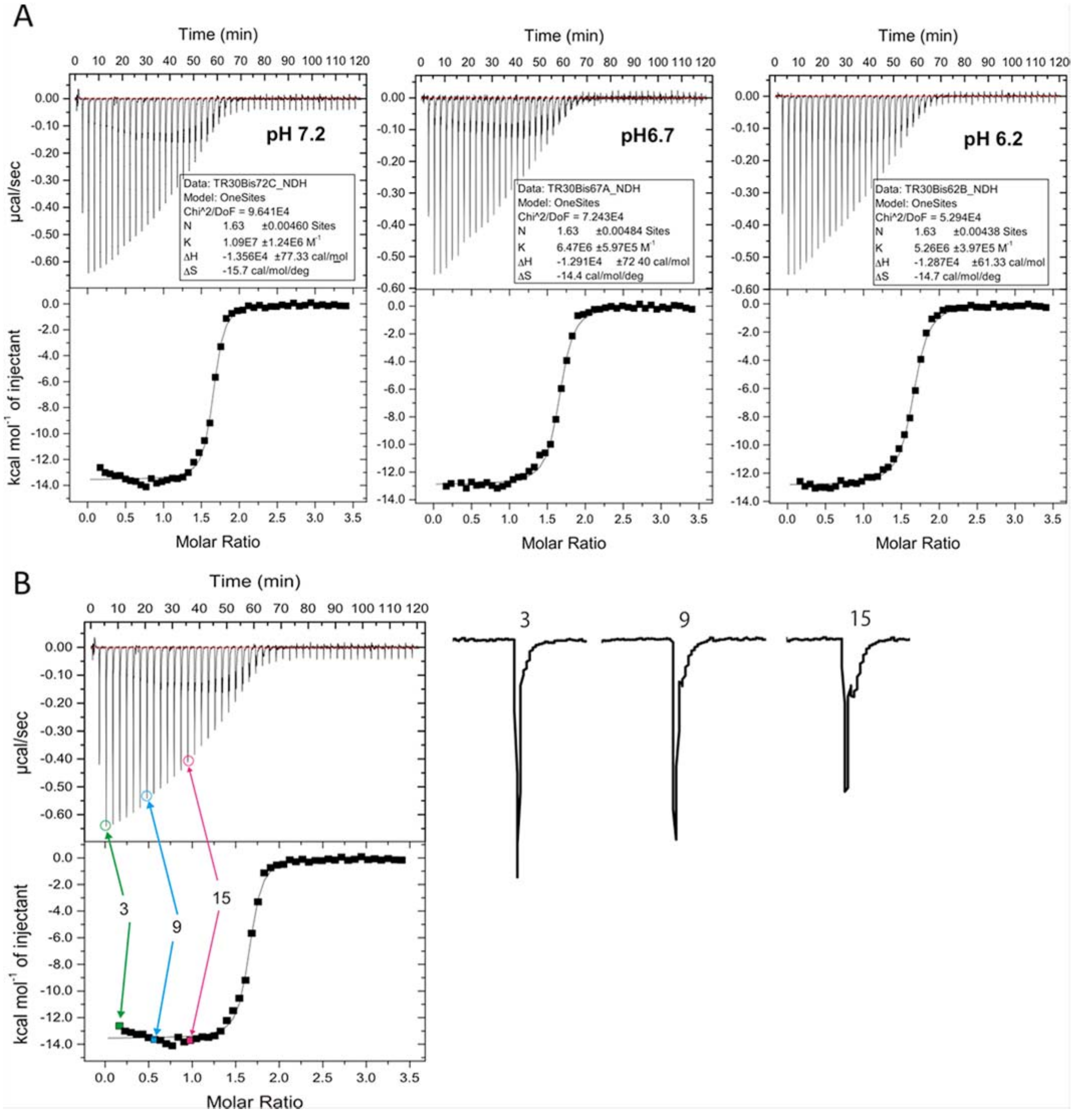

Attached Figure 5 ITC results analyzed with Origin Software

(A) The ITC data shown in Supplementary Figure 1A were analyzed with Origin. Note that the thermodynamic parameters shown in insets are similar to those with SEDPHAT.

(B) Comparison of the peak shapes at the 3rd, 9th and 15th injections in the ITC raw data shown in (A), left (pH 7.2) .

.

Comment 2.

The authors determined the number of zinc binding sites using a 1:1 model for the fitting in SEDPHAT, etc. Authors should explain in details the fitting of the ITC data and how they determined the number of binding sites. Moreover, the authors showed

that Zn promotes oligomerization of Erp44. How can authors distinguish in the ITC the heat of the binding from the heat of the oligomerization?

Response: Concentration correction factors (*r*) in SEDPHAT correspond to the *n* (stoichiometry) parameter in Origin. By analyzing the observed ITC data using a 1:1 binding model in SEDPHAT, we obtained stoichiometry (*n*), apparent binding affinity (K_a) and enthalpy change (ΔH) for Zn^{2+} binding to ERp44. This procedure is a conventional process as described in the previous literature (Brautigam et al., ***Nature protocol*** 2010 etc). We also tested to analyze the ITC data using a 1:1 binding model in Origin. Apparent stoichiometry (*n*) was determined by fitting three parameters *n*, ΔH and K_a to the observed ITC data. In the results, both the analyses with Origin and SEDPHAT reached the same conclusion that ERp44 binds 1.5 molar equivalent of Zn^{2+}. Our procedure of the data fitting has been additionally described in the Method section of the revised manuscript (Page 40, second paragraph).

As revealed by the present crystallographic study, Zn^{2+} binding to site 2 accompanies ERp44 dimerization concomitantly. This means that it is in principle impossible to distinguish the heat of dimerization from that of Zn^{2+} binding to site 2. Also, due to the presence of the positive cooperativity, it is theoretically difficult to determine enthalpy changes of Zn^{2+} binding to site 1 and site 2 separately (Tochtrop et al., ***PNAS*** 99, 1847-1852, 2002). As described in our response to the previous comment, however, the peak shape changes during the titration (attached Fig. 5B) may reflect the varying ratios of the Zn^{2+} binding to site 1 and the subsequent ERp44 dimerization. Although we do not mention about this idea in the revised manuscript due to weak evidence, we are ready to discuss this issue in the manuscript if requested by the editor and/or reviewers.

Comment 3.

The same could be applied to the ANS assay. The authors associate the increase in fluorescence to an increase in hydrophobicity due to a conformational change. That could be applied to the case of the experiments at different pHs, but in the presence of Zn, the authors should have into account the oligomerization.

Response: The referee is in principle right in stating that changes in ANS binding could reflect dimerization. However, as revealed by the present crystallographic study, Zn^{2+}-induced conformational changes further expose a hydrophobic patch near the boundary of the **b** and **b'** domains in ERp44 (Fig. 4B). This hydrophobic patch is not concealed in the dimer interface. Thus, the increase of ANS fluorescence intensity upon Zn^{2+}-mediated dimerization can be well explained by the present crystallographic data (Fig. 3A). This supplementary explanation has been inserted into the revised text (page 14, lines 13-14).

Comment 4.

Authors affirmed, "The Zn2+-bridged non-covalent homodimer is consistent with the results of the ITC and SEC analyses". From ITC data the authors can't deduce the oligomerization. Typical ITC oligomerization experiments should be done (injecting high concentration protein to buffer). These experiments will provide with the Kd of dimerization, lacking in the model.

Response: According to this suggestion, we performed the dissociation titration experiments using 10-100 μM ERp44 solution containing two-fold molar excess of Zn^{2+} (see new Supplementary Fig 8D). In the result, no significant heat changes were observed during the dilution titration. Thus, the Zn^{2+}-mediated ERp44 dimers seem to hardly disassemble at concentration of 0.1 μM (100-fold dilution of titrant), implying that the dissociation constant for ERp44 dimerization is below 0.1 μM. This information is added in the revised manuscript (page 13, lines 10-15, new Supplementary Fig 8D).

Comment 5.

Some SEC experiments were conducted at pH 7, they should have repeated at pH 6.2 in order to compare oligomerization in both conditions.

Response

As described in our response to comment 2 by Reviewer #3, we performed the SEC analysis for detection of the Zn^{2+}-induced ERp44 dimerization at pH 6.2 (Figure 4C, blue dotted line) and pH 6.7 (Supplementary Figure 8C). This information was not

clearly described in the original manuscript, and therefore we added the information in the legends to those figures. To address this comment appropriately, we also performed additional SEC experiments at pH 7.2, 6.7 and 6.2 (Supplementary Figure 1B in the revised manuscript). The results clearly demonstrate that ERp44 forms a Zn^{2+}-mediated homodimer in the pH range of 6.2 to 7.2. This information is added to the revised text (page 5, lines 15-16).

Comment 6.

The authors affirmed that Zn binding is sequential. Zn-binding ITC data analysis should be re-analyzed using this assumption.

Response

The sequential binding model in Origin Software forces us to enter an integral number as the number of ligand-binding sites per a molecule. This means that as far as we use this program, it is in principle impossible to analyze the ITC data with the sequential Zn^{2+}-binding model assuming a binding stoichiometry of 1:1.5. Instead, we performed a sequential fitting of the ITC data with a new fitting program AFFINImeter (https://www.affinimeter.com/). However, we could not obtain the reasonable parameters with good χ^2 values (attached figure 6). In the positively cooperative binding system, determination of a unique set of sequential binding parameters is generally believed to be quite difficult because the binding curve can be fitted with good χ^2 values by divergent sets of the parameters. An apparent single phase binding curve in the ITC data with positive cooperativity does not have sufficient information to deconvolute the first and second binding enthalpy changes (Tochtrop et al., ***PNAS*** 99, 1847-1852, 2002).

Instead, as described in our response to comment 1 by Reviewer 1, we performed a competition assay using the fluorimetric dye Mag-fura-2 (MF2) (attached figure 1). Moreover, we recalculated the Scatchard plots and the saturation curves based on the observed ITC data (Supplementary Fig. 8A, B, C), which strongly supports the positive cooperativity in sequential Zn^{2+}-binding to sites 1 and 2 of ERp44.

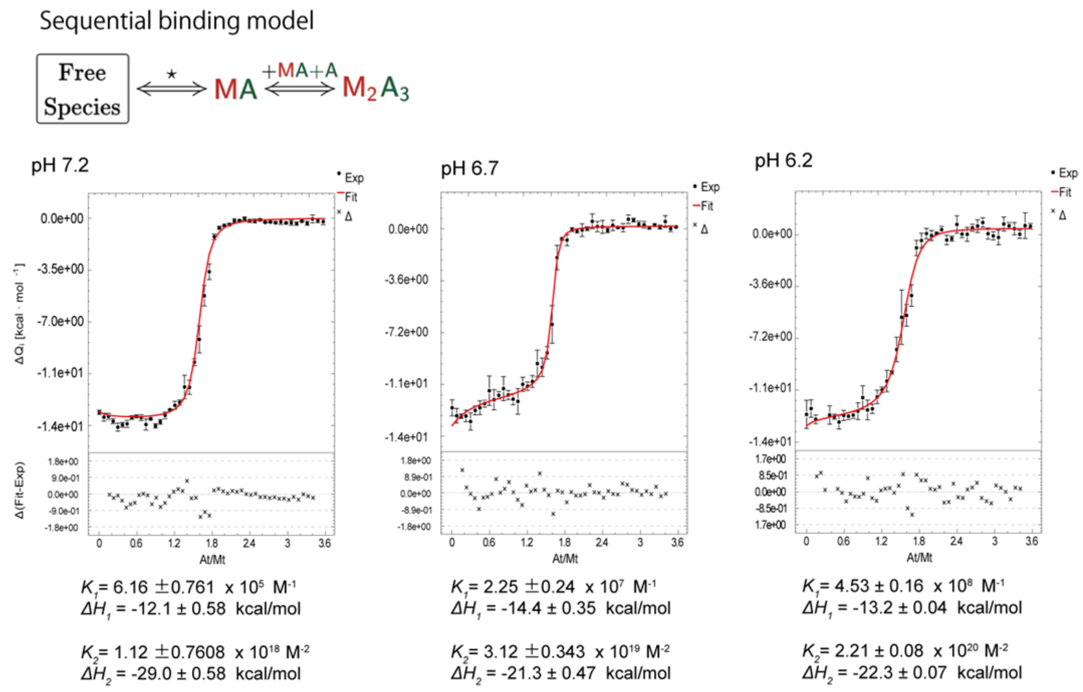

Attached Figure 6 Sequential fitting of the ITC data with Affinimeter program. Binding isotherm data measured at pH 7.2, 6.7 and 6.2 were fitted using the sequential binding model illustrated at the top of this figure.

Comment 7.

Erp44-Ero1a interaction in the presence of Zn was conducted in Zn concentration not saturating for Erp44. Furthermore, authors should have in mind the heat of oligomerization after injecting Erp44 into Ero1a and ZnCl2 cell. Additionally, experiments at pH=7.2 will help to strengthen the proposed model.

Response: The present SEC analysis (Fig 4E) indicated that in presence of Zn^{2+}, ERp44 more preferentially forms heterodimers with Ero1α than homodimers. If much excess Zn^{2+} over Ero1α exists in the ITC cell, ERp44 will form the homodimer after all Ero1α is bound to ERp44. In order to exclusively observe the heat change caused by the Zn^{2+} dependent interaction between ERp44 and Ero1α, Zn^{2+} equimolar to Ero1α was added in this experiment.

We observed that Zn^{2+} enhanced the interaction between ERp44 and Ero1α at pH7.2 as well as at pH 6.2 (see attached figure 7). This result may suggest that the ERp44-Ero1α complex is unlikely to resolve after returning to the ER. However, considering that ZnT proteins that conduct Zn^{2+} uptake are located in the Golgi, but not in the ER, the concentration of Zn^{2+} is likely lower in the ER than that in the Golgi. Without Zn^{2+}, affinity of ERp44 for Ero1α is much lower at pH7.2 (K_d = ~11 μM) than at pH6.2 (K_d = ~1.3 μM), as demonstrated by our previous study (Watanabe, et al. ***PNAS*** 2017). Thus, our proposed model of pH- and Zn^{2+}-dependent ERp44 regulation illustrated in Fig. 6 is well supported by a series of the present and previous data. Along this line of story, we discussed our model in Discussion section (page 21, second paragraph).

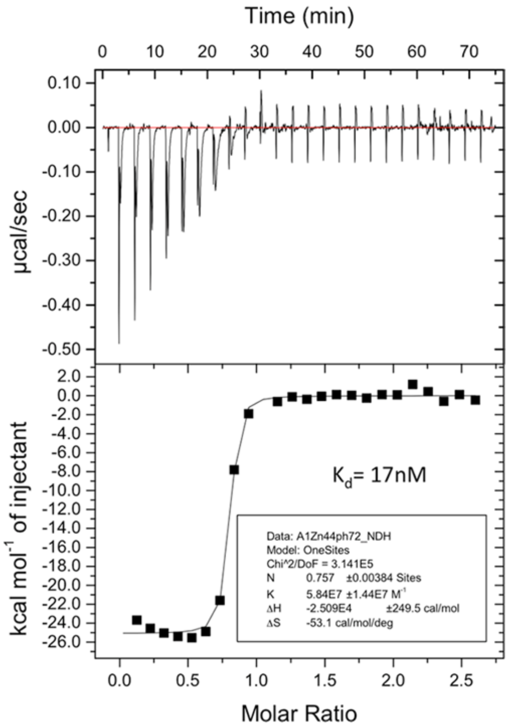

Attached Figure 7 ITC analysis for interaction between ERp44 and Ero1□ in the presence of Zn^{2+} at pH7.2

ITC raw data (upper) and binding isotherm data (lower) for titration of ERp44 into a mixture of Ero1α and Zn^{2+} at pH 7.2.

Comment 8-1.

Minor comments:

Authors should add the standard deviation of the ITC parameter values and some symbols are missing in the scale of supplementary figure 1 A.

Response: As described in the legend to Supplementary Fig. 1A, we employed SEDPHAT to perform the global analysis of the ITC data for Zn^{2+}-binding to ERp44 at pH7.2, 6.7 and 6.2. This software does not provide the standard deviation of the ITC parameters. Instead, we added the sentence "The calculated thermodynamic parameters are shown with 68.3 % confidence interval in brackets" in the legends to Fig. 1B, Fig. 4E and supplementary Fig. 1A, as did the previous paper (Brautigam et al., ***Nature Protocol*** 2010).

Comment 8-2.

In fig.2 the size of Ero1α looks higher in OUT. Authors should explain it.

Response: Ero1 contains two N-glycosylation sites (Bertoli et al., ***J. Biol. Chem.*** 2004). Its mobility decreases when the N-glycans are processed by Golgi resident enzymes (Otsu et al., ***Antiox. Redox Signal.*** 2006). TPEN could inhibit the activity of a galactosyltransferase, hence preventing the processing of N-glycoproteins. Also, we cannot exclude the possibility that the oxidase undergoes O-linked glycosylation. Complete Zn^{2+} depletion by TPEN may inactivate O-glycosylation enzymes in the Golgi, of which activity requires metal ions (Yingnan Zhang, et al., ***J. Biol. Chem.*** 2001). One more possibility is phospholylation of Ero1α, as reported recently (Zhang et al., ***EMBO J*** 37, 1-16, 2018). Whatever the reason, the mobility difference shows that Ero1α is actively secreted and not released by cell death. We briefly addressed this issue in the legend to Figure 2b.

Comment 8-3.

In Scatchard plot R(i)= X_{bound}(i) (number of bound ligand)/M_t(i) for multivalent ligand, as in this case.

Response:

In Scatchard analysis, B/L is plotted as a function of B, where B is the concentration of

bound ligand and L is the concentration of free ligand. Thus, a maximum concentration of bound ligand and $-1/K_d$ are provided on the X intercept and the slope of the plot, respectively. B is often replaced with R = B/Mt (a fraction of bound ligands per protein molecule). Plotting R/L as a function of R gives the apparent stoichiometry (n) on the X-intercept (Grant, ***Method, Enthoymol***, 2004). In accordance with this, we plotted $R(i) = X_{bound}(i) / M_t(i))$ against $R(i) / X_{free}(i)$. Consequently, the X-intercept provided the stoichiometry of 1.5 for Zn^{2+} binding to ERp44 as expected (supplementary Fig. 8a).

Comment 9.

In summary, the authors presented a model for the regulation of Erp44 by Zn. Taking into account that the model relies heavily on ITC results, I think that authors should address some of the above comments before publishing the manuscript in Nature Communications.

Response:

We appreciate the critical yet constructive comments on the ITC analysis by this reviewer. We carefully reconsidered and addressed all issues relevant to the ITC analysis and revised the manuscript accordingly, as described above.

REVIEWERS' COMMENTS:

Reviewer #2 (Remarks to the Author):

I am satisfied that all comments from my review of the original manuscript have been appropriately addressed. In my view, this manuscript is now suitable for publication.

Reviewer #3 (Remarks to the Author):

The authors have taken great efforts to address the previous issues. I would just request the authors to make two minor changes.
1. Based on the new supplemental data, the endogenous ERp44 mainly localizes on ERGIC, not ER. Please clarify in the text (last line of page 6) that excess Zn would re-localize the ERp44 to the ERGIC, not ER.
2. Please define the ERGIC when this acronym was first appeared in the text.

Reviewer #4 (Remarks to the Author):

After the revision and the additional experiments performed, the authors have clarified my main concerns about the manuscript. Even though the authors couldn't carry out successfully some of the ITC experiments requested by me in order to support their model, they solve this issue using others methods. I am aware of the limitation of the ITC method, as authors have pointed out in their response, but the authors have solved my doubts about some results using others approaches. I think that it should be enough in order the manuscript to be published in Nature Communications.

Manuscript No.; NCOMMS-18-22406A

Response to reviewers' comments

<u>Reviewer #3:</u>

The authors have taken great efforts to address the previous issues. I would just request the authors to make two minor changes.

Comment 1

Based on the new supplemental data, the endogenous ERp44 mainly localizes on ERGIC, not ER. Please clarify in the text (last line of page 6) that excess Zn would re-localize the ERp44 to the ERGIC, not ER.

Response: We agree that excess Zn^{2+} re-localized ERp44 mainly to the ERGIC. The corresponding sentence has been modified as follows (page 6 bottom to page 7 top).

Subsequent addition of excess $ZnSO_4$ was sufficient to re-localize endogenous ERp44 primarily to the ERGIC (Fig. 1e, right panels and 1f).

Comment 2

Please define the ERGIC when this acronym was first appeared in the text.

Response: The word "ERGIC" was defined as "ER-Golgi intermediate compartment" when it appeared first in the text (page 6, lines 9-10).
